# Supplementary material for: A Chromosome-Level Genome Assembly of the European Beech (Fagus sylvatica) Reveals Anomalies for Organelle DNA Integration, Repeat Content and Distribution of SNPs
Source: Front Genet. 2022 Feb 8;12:691058. doi: 10.3389/fgene.2021.691058 (PMC8862710; doi:10.3389/fgene.2021.691058)
Supplement: Supplementary file 2 [file Supplementary_file_6.pdf › Supplementary_file_6.pdf]

Supplementary file 6. Genotyping data. List of 4127 SNPs and their associated linkage groups based on R_qtl (second raw) and JoinMap (third raw) analyses.

SNP_ID	snp_B0029	snp_B0043	snp_B0050	snp_B0051	snp_B0067	snp_B0087	snp_B0099	snp_B0106	snp_B0160	snp_B0193	snp_B0203	snp_B0205	snp_B0249	snp_B0265	snp_B0302	snp_B0330	snp_B0349	snp_B0376	snp_B0381	snp_B0392	snp_B0417	snp_B0418
LG_Rqtl	1	1	1	1	1	1	1	1	1	1	1	1	1	1	1	1	1	1	1	1	1	1
LG_JoinMap	LG3	--	--	--	--	--	--	--	--	--	--	--	--	--	--	--	--	--	--	--	--	--
SSB002	AA	AB	AA	AB	AA	AA	AA	AA	AB	AB	AB	AA	AB	AB	AB	AB	AA	AB	AA	AA	AB	AA
SSB003	AB	AB	AB	AB	AB	AB	AB	AB	AB	AA	AB	AB	AA	AB	AB	AB	AB	AB	AB	AB	AB	AB
SSB007	AA	AB	AB	AB	AB	AA	AA	AA	AB	AA	AA	AB	AA	AA	AA	AB	AA	AB	AA	AB	AB	AA
SSB010	AA	AA	AA	AB	AA	AA	AB	AA	AA	AA	AA	AA	-	AA	AB	-	AA	-	-	AB	-	AB
SSB014	AB	AB	AB	AA	AB	AB	AA	AA	AB	AA	AA	AB	AA	AB	AA	AB	AA	AB	AA	AA	AB	AB
SSB016	AA	AA	-	-	-	AA	-	AB	AA	AB	AA	-	AA	AA	AA	-	-	AB	AA	AA	AA	-
SSB020	AA	AB	AA	AA	AB	AB	AB	AB	AA	AA	AB	AA	AA	AB	AA	AA	AB	AB	AA	AB	AA	AA
SSB022	AA	AB	AB	AB	AB	AB	AB	AA	AB	AB	AB	AB	AA	AB	AB	AB	AA	AA	AB	AA	AA	AA
SSB024	AA	AA	AA	AA	AA	AB	AA	AA	AA	AB	AB	AA	AB	AB	AB	AA	AA	AB	AA	AA	AA	AB
SSB030	AA	AA	AA	AA	AB	AB	AB	AA	AA	AB	AB	AA	AA	AA	AB	AA	AB	AB	AB	AB	AA	AA
SSB031	AA	AA	AA	AB	AB	AB	AB	AB	AA	AB	AB	AA	AB	AB	AB	AB	AB	AB	AB	AB	AB	AA
SSB033	AA	AB	AB	AB	AB	AB	AB	AA	AB	AB	AA	AA	AB	AB	AB	AB	AA	AB	AB	AB	AA	AA
SSB039	AA	AA	AA	AA	AA	AB	AA	AA	AA	AB	AB	AA	AA	AA	AA	AA	AA	AB	AA	AA	AA	AB
SSB041	AB	AB	AB	AA	AB	AB	AA	AA	AA	AB	AA	AA	AA	AA	AB	AA	AB	AB	AA	AB	AA	AA
SSB048	AA	AA	AA	AB	AB	AA	AB	AA	AA	AA	AA	AA	AB	AA	AB	AA	AB	AB	AB	AB	AA	AB
SSB049	AA	AB	AB	AB	AA	AA	AA	AA	AA	AB	AB	-	AA	AA	AB	AB	AA	AB	AB	AB	AA	AB
SSB052	AB	AA	AB	AB	AA	AB	AA	AA	AB	AA	AB	AB	AB	AA	AB	AB	AB	AA	AB	AB	AA	AB
SSB070	AB	AA	-	-	-	AA	-	AB	AB	AA	AA	-	-	AB	-	-	-	-	AB	AA	-	-
SSB075	AB	AB	AB	AB	AB	AB	AB	AB	AB	AB	AB	AB	AB	AB	AB	AB	AB	AB	AB	AB	AB	AB
SSB077	AA	AB	AB	AB	AB	AA	AB	AB	AB	AB	AB	AA	AA	AB	AB	AB	AA	AB	AB	AA	AA	AA
SSB080	AA	AB	AB	AB	AB	AB	AB	AA	AB	AB	AB	AA	AA	AB	AB	AB	AB	AB	AB	AB	AB	AA
SSB084	AB	AA	AA	AB	AB	AB	AA	AA	AA	AA	AB	AB	AB	AB	AB	AB	AB	AA	AB	AB	AA	AB
SSB086	AA	AB	-	-	-	AA	AA	AB	AA	-	AB	AA	-	AA	-	-	-	-	-	AB	AA	-
SSB097	AB	AB	AB	AB	AB	AB	AB	AA	AA	AA	AB	AB	AB	AB	AB	AB	AB	AA	AB	AB	AA	AB
SSB098	AB	AA	AA	AB	AA	AB	AA	AA	AA	AA	AB	AB	AA	AA	AB	AA	AB	AA	AA	AA	AA	AA
SSB110	AB	AB	AB	AB	AA	AB	AA	AB	AA	AB	AA	AB	AB	AA	AB	AB	AB	AA	AB	AA	AB	AA
SSB111	AB	AB	AB	AB	AB	AA	AB	AB	AA	AB	AA	AA	AB	AA	AB	AA	AB	AB	AB	AA	AB	AA
SSB119	AA	AB	AB	AB	AB	AA	AB	AB	AA	AB	AA	AA	AA	AA	AB	AB	AA	AB	AB	AA	AA	AA
SSB122	AA	AA	AB	AB	-	AA	AB	AA	AA	AB	AB	-	-	AA	AB	-	AB	AB	AB	AB	-	AB
SSB123	AB	AA	AB	-	-	AA	-	AB	AB	AA	AB	-	-	AB	-	-	-	-	-	AA	-	AB
SSB127	AB	AB	AA	AB	AA	AA	AB	AA	AA	AA	AA	AA	AB	AB	AA	AA	AB	AA	AA	AA	AA	AB
SSB129	AB	AB	AA	AB	AA	AA	AB	AA	AA	AA	AA	AB	AB	AA	AB	AB	AA	AA	AB	AA	AA	AB
SSB130	AA	AB	-	AB	AB	AA	AB	AB	AB	AA	AA	-	-	AB	AA	AB	-	AA	AB	AA	AA	-
SSB132	AB	AB	AB	AB	-	AB	AB	AB	AB	AA	AB	-	AB	AB	AB	-	-	AB	AB	AB	-	AB
SSB137	AA	AA	AA	AB	-	AA	AA	AA	AA	AB	AB	AA	-	AB	AB	-	AA	-	AA	AA	-	-
SSB145	AB	AA	AA	AB	AA	AB	AA	AB	AA	AB	AB	AA	AB	AA	AB	AA	AB	AB	AA	AB	-	AB
SSB148	AB	AA	AA	AB	AB	AB	AA	AB	AA	AB	AB	AB	AB	AB	AB	AA	AB	AB	AB	AB	AB	AB
SSB149	AA	AB	AA	AA	AB	AB	AB	AB	AA	AB	AB	AA	AA	AB	AA	AA	AB	AB	AA	AB	AB	AA

[illegible]

snp_B1351	snp_B1391	snp_B1426	snp_B1427	snp_B1434	snp_B1449	snp_B1454	snp_B1467	snp_B1499	snp_B1520	snp_B1571	snp_B1592	snp_B1597	snp_B1602	snp_B1610	snp_B1615	snp_B1621	snp_B1655	snp_B1675
1	1	1	1	1	1	1	1	1	1	1	1	1	1	1	1	1	1	1
--	--	--	--	--	LG3	--	--	--	LG3	--	--	--	--	--	LG3	--	--	--
AB	AA	AB	AA	AB	AB	AB	AB	AB	AB	AB	AB	AA	AA	AA	AB	AA	AB	AA
AB	AB	AA	AB	AB	AB	AB	AB	AB	AB	AB	AB	AB	AB	AB	AB	AB	AB	AB
AA	-	AA	AB	AA	AA	AA	AB	AB	AB	AB	AB	AB	AB	AB	AB	AB	AB	AB
AB	AB	-	-	AB	AA	AB	-	AB	AB	AA	-	AB	AB	-	AA	AA	AB	AA
AA	AB	AB	AA	AB	AB	AB	AB	AA	AA	AA	AB	AB	AA	AB	AA	AB	AB	AB
-	AB	-	-	AA	AA	AB	-	AA	AA	AB	-	AA	AA	AA	AA	AA	AA	-
AA	AB	AB	AB	AB	AA	AB	AA	AB	AB	AB	AA	AB	AB	AA	AA	AB	AA	AA
AA	AA	AA	AA	AA	AB	AA	AB	AA	AA	AA	AA	AA	AB	AB	AB	AA	AA	AB
AA	AA	AB	AA	AB	AA	AB	AA	AB	AA	AA	AA	AA	AA	AA	AB	AB	AA	AA
AB	AA	AB	AA	AB	AA	AB	AA	AB	AB	AA	AA	AA	AB	AA	AA	AA	AA	AB
AA	AB	AB	AA	AB	AA	AB	AA	AB	AB	AA	AA	AA	AA	AA	AA	AA	AA	AB
AB	AA	AB	AA	AB	AA	AB	AB	AA	AB	AA	AA	AA	AB	AB	AB	AB	AB	AA
AB	AA	AB	AA	AB	AB	AA	AB	AA	AB	AA	AA	AB	AA	AB	AB	AA	AA	AB
AB	AA	AB	AB	AB	AA	AB	AA	AB	AB	AA	AA	AB	AB	AB	AA	AB	AA	AB
AB	AA	AB	AA	AB	AA	AB	AA	AB	AB	AA	AA	AA	AB	AA	AA	AA	AA	AA
AB	AB	AB	AB	AA	AA	AA	AB	AB	AB	AB	AA	AB	AB	AB	AB	AB	AA	AB
AA	AA	AA	AA	AA	AB	AA	AB	AB	AB	AA	AB	AA	AA	AB	AB	AB	AB	AB
-	-	-	AB	AA	AB	AA	-	AA	AA	AB	-	AB	AA	AA	AA	-	AB	AB
AB	AB	AB	AB	AB	AB	AB	AB	AB	AB	AB	AB	AB	AB	AB	AB	AB	AB	AB
AB	AB	AB	AB	AB	AB	AA	AB	AB	AA	AB	AA	AB	AB	AB	AB	AB	AB	AB
AB	AA	AB	AB	AA	AB	AA	AB	AB	AB	AB	AA	AB	AB	AB	AB	AB	AB	AB
AB	AA	AA	AB	AA	AB	AA	AA	AB	AB	AA	AB	AB	AA	AA	AB	AB	AB	AB
AB	AA	-	-	AB	AA	-	-	-	AB	AA	-	AA	AB	-	AB	-	AA	AB
AA	AA	AA	AA	AB	AA	AB	AA	AB	AB	AA	AB	AB	AB	AA	AB	AB	AA	AB
AA	AA	AA	AB	AA	AA	AA	AA	AA	AB	AA	AA	AB	AA	AA	AB	AB	AA	AA
AB	AB	AA	AA	AB	AA	AB	AB	AA	AA	AB	AB	AB	AB	AB	AA	AA	AA	AB
AB	AB	AB	AB	AB	AA	AB	AB	AB	AB	AB	AA	AB	AB	AB	AA	AB	AA	AA
AB	AB	AA	AB	AB	AA	AB	AA	AA	AB	AB	AA	AB	AB	AB	AA	AB	AA	AA
AB	AA	AB	AA	AB	AA	-	AA	AB	AB	AB	AA	AB	AB	AB	AB	AB	AA	AB
AA	-	AA	-	AA	AB	-	AA	AB	AB	AB	-	AB	AB	AB	AA	-	AB	-
AA	AA	AA	AA	AA	AB	AA	AA	AA	AB	AA	AB	AA	AB	AA	AB	AB	AB	AB
AA	AA	AA	AA	AA	AB	AA	AA	AA	AA	AA	AB	AA	AB	AA	AB	AA	AB	AB

snp_B1682	snp_B1693	snp_B1710	snp_B1717	snp_B1723	snp_B1736	snp_B1755	snp_B1758	snp_B1769	snp_B1770	snp_B1771	snp_B1773	snp_B1790	snp_B1794	snp_B1818	snp_B1828	snp_B1835	snp_B1853	snp_B1883	snp_B1885	snp_B1906	snp_B1911	snp_B1926	snp_B1950	snp_B1965	snp_B1986	snp_B1992	snp_B2004	snp_B2009	snp_B2025	snp_B2031	snp_B2034
1	1	1	1	1	1	1	1	1	1	1	1	1	1	1	1	1	1	1	1	1	1	1	1	1	1	1	1	1	1	1	1
--	--	LG3	--	LG3	LG3	LG3	--	--	--	--	LG3	--	--	--	--	LG3	LG3	--	--	--	--	--	--	--	--	--	--	--	LG3	--	--
AA	AB	AA	AA	AA	AB	AB	AA	AA	AB	AA	AA	AB	AB	AA	AA	AA	AA	AA	AA	AB	AA	AB	AA	AB	AB	AA	AB	AB	AB	AA	AA
AB	AB	AB	AA	AB	AB	AB	AB	AB	AB	AB	AB	AB	AB	AB	AB	AB	AB	AB	AA	AB	AB	AB	AB	AB	AB	AB	AB	AB	AB	AB	AB
AB	AB	AB	AA	AB	AA	AA	AB	AB	AB	AB	AB	AA	AB	AB	AB	AA	AB	AB	AA	AB	AB	AB	AA	AB	AB	AB	AB	AB	AB	AB	AB
AB	AB	AA	AA	AB	AA	AA	-	AA	AA	AA	AB	AB	AB	AA	AA	AA	AB	AB	AA	AA	AA	AA	AA	AB	AB	AA	AB	AA	AB	AB	AA
AA	AA	AB	AB	AA	AB	AB	AB	AB	AB	AB	AA	AB	AA	AA	AA	AB	AB	AA	AB	AA	AA	AB	AB	AA	AA	AA	AA	AA	AA	AA	AB

snp_B2039	snp_B2049	snp_B2061	snp_B2066	snp_B2085	snp_B2086	snp_B2100	snp_B2103	snp_B2112	snp_B2113	snp_B2137	snp_B2145	snp_B2163	snp_B2173	snp_B2176	snp_B2181	snp_B2220	snp_B2225	snp_B2255	snp_B2258	snp_B2275	snp_B2282	snp_B2371	snp_B2377	snp_B2383	snp_B2400
1	1	1	1	1	1	1	1	1	1	1	1	1	1	1	1	1	1	1	1	1	1	1	1	1	1
--	--	--	--	--	--	--	--	--	--	--	--	--	--	--	--	--	LG3	LG3	--	--	LG3	--	--	--	--
AB	AB	AA	AA	AA	AB	AA	AA	AB	AA	AA	AA	AB	AA	AA	AA	AB	AA	AB	AA	AA	AA	AA	AB	AB	AB
AA	AB	AB	AB	AB	AB	AB	AA	AB	AB	AB	AB	AB	AA	AB	AB	AB	AB	AB	AA	AB	AB	AB	AB	AB	AB
AA	AB	AB	AB	AB	AB	AB	AA	AB	AB	AB	AB	AB	AA	AB	AB	AB	AA	AA	AB	AA	AB	AB	AB	AA	AB
AB	AA	AB	AA	AA	AA	AA	AB	AA	AB	AB	AB	AA	AB	AB	AA	AB	AA	AA	AB	AA	-	AA	AA	AB	AB
AA	AA	AA	AB	AB	AA	AA	AA	AA	AA	AA	AA	AA	AA	AA	AB	AA	AA	AB	AA	AB	AB	AB	AB	AB	AA

snp_B2407	snp_B2445	snp_B2510	snp_B2516	snp_B2531	snp_B2533	snp_B2541	snp_B2547	snp_B2549	snp_B2558	snp_B2559	snp_B2572	snp_B2601	snp_B2602	snp_B2613	snp_B2621	snp_B2622	snp_B2623	snp_B2625	snp_B2654	snp_B2670	snp_B2718	snp_B2772
1	1	1	1	1	1	1	1	1	1	1	1	1	1	1	1	1	1	1	1	1	1	1
--	--	--	--	--	--	--	--	--	--	LG3	--	--	--	--	--	LG3	--	--	LG3	--	--	--
AB	AA	AA	AA	AB	AA	AA	AB	AB	AB	AA	AB	AB	AA	AB	AB	AB	AA	AB	AB	AA	AA	AB
AB	AB	AB	AB	AB	AB	AB	AB	AB	AB	AB	AB	AB	AB	AB	AB	AB	AB	AB	AB	AB	AB	AB
AA	AA	AB	AB	AA	AB	AB	AB	AB	AA	AB	AA	AB	AB	AB	AA	AA	AB	AB	AB	AA	AB	AA
AA	AB	AA	AB	AA	AA	AA	AB	AA	AA	-	AA	AB	AA	AA	AB	AA	AB	AA	AA	-	AA	AA
AB	AA	AA	AB	AB	AA	AA	AA	AB	AB	AA	AB	AA	AA	AA	AB	AB	AA	AA	AA	AB	AA	AA
AA	-	AA	AB	AA	AB	AA	AA	AA	-	-	-	AA	AB	AA	AB	AB	AA	-	-	AA	AB	AA
AA	AB	AB	AB	AA	AA	AB	AB	AA	AA	AA	AA	AA	AA	AB	AB	AA	AB	AB	AA	AB	AB	AB
AB	AA	AB	AB	AA	AB	AB	AA	AB	AB	AA	AB	AA	AB	AB	AA	AB	AA	AA	AA	AB	AA	AB
AB	AA	AB	AB	AA	AA	AA	AA	AB	AB	AA	AA	AA	AB	AB	AB	AB	AA	AB	AB	AB	AA	AA
AA	AA	AA	AB	AA	AB	AB	AA	AA	AA	AB	AA	AB	AA	AB	AB	AA	AB	AB	AB	AA	AA	AA
AA	AB	AB	AA	AB	AA	AB	AB	AA	AA	AB	AB	AB	AA	AB	AB	AA	AB	AB	AA	AB	AA	AB
AB	AA	AB	AB	AB	AB	AB	AB	AB	AB	AB	AA	AB	AA	AB	AB	AA	AB	AB	AA	AA	AA	AB
AA	AA	AB	AB	AB	AB	AA	AA	AA	AA	AA	AB	AB	AA	AB	AB	AA	AA	AA	AA	AB	AA	AA
AA	AA	AB	AB	AA	AA	AA	AA	AA	AA	AB	AA	AB	AA	AA	AB	AA	AB	AB	AB	AA	AB	AA
AA	AB	AA	AB	AA	AB	AB	AB	AA	AA	AA	AA	AB	AB	AB	AA	AA	AB	AA	AB	AA	AA	AA
AA	AA	AB	AB	AA	AB	AB	AB	AA	AA	AB	AB	AB	AB	AB	AB	AA	AB	AA	AA	AB	AB	AB
AB	AA	AA	AA	AB	AA	AA	AA	AB	AB	AB	AB	AA	AA	AB	AA	AB	AB	AB	AB	AB	AA	AA
AB	-	AA	AB	AB	AB	AA	AB	AA	AB	-	-	AA	AB	AA	-	AB	-	AA	-	-	AB	AB
AB	AB	AB	AB	AB	AB	AB	AB	AB	AB	AB	AB	AB	AB	AB	AB	AB	AB	AB	AB	AB	AB	AB
AB	AA	AB	AB	AB	AB	AB	AB	AB	AB	AA	-	AA	AB	AA	AB	AA	AB	-	AA	AA	AB	AB
AB	AB	AB	AB	AA	AB	AB	AB	AB	AB	AB	AA	AB	AA	AB	AB	AA	AB	AB	AB	AA	AA	AB
AB	AB	AB	AB	AA	AB	AA	AB	AB	AB	AB	AB	AB	AA	AB	AA	AB	AB	AB	AB	AB	AA	AB
AA	-	AB	AB	AA	AA	AB	AB	AA	AA	AB	-	AB	AA	-	AB	AA	-	-	-	-	AA	AB
AB	AB	AA	AB	AB	AA	AA	AB	AB	AB	AB	AB	AB	AA	AB	AA	AB	AA	AB	AB	AB	AA	AB

snp_B2776	snp_B2793	snp_B2807	snp_B2808	snp_B2829	snp_B2838	snp_B2840	snp_B2844	snp_B2849	snp_B2854	snp_B2876	snp_B2890	snp_B2907	snp_B2914	snp_B2918	snp_B2923	snp_B2927	snp_B2932	snp_B2950	snp_B2984	snp_B2992	snp_B2999	snp_B3017	snp_B3044	snp_B3058	snp_B3071
1	1	1	1	1	1	1	1	1	1	1	1	1	1	1	1	1	1	1	1	1	1	1	1	1	1
--	LG3	--	LG3	--	--	--	--	--	--	--	--	LG3	--	--	LG3	--	--	--	--	--	--	--	--	--	--
AB	AA	AB	AA	AA	AB	AB	AB	AA	AA	AA	AA	AB	AA	AA	AB	AA	AA	AA	AB	AB	AB	AA	AB	AB	AB
AB	AB	AB	AB	AB	AB	AB	AB	AB	AB	AB	AB	AB	AB	AB	AB	AB	AB	AB	AB	AB	AB	AB	AB	AB	AB
AA	AA	AA	AA	AB	AA	AA	AA	AB	AA	AB	AB	AA	AB	AA	AB	AB	AA	AA	AA	AA	AA	AB	AA	AA	AB
AA	AA	AB	AA	AA	AB	-	AA	AB	AB	AA	-	AA	AA	AA	AB	AB	AA	AA	AA	-	AB	AB	AA	-	AB
AB	AB	AA	AB	AB	AA	AB	AB	AA	AA	AA	AA	AB	AA	AA	AA	AA	AB	AA	AB	AA	AB	AB	AB	AB	AA
AA	AA	AA	AA	AA	AA	-	AA	AA	AA	AA	-	AA	AA	-	-	AA	AA	AA	AA	-	AA	AB	AA	AA	-
AA	AA	AA	AA	AA	AB	AB	-	AA	AA	AA	AA	AA	AA	AA	AB	AB	AB	AB	AA	AB	AA	AB	AA	AA	AB
AA	AB	AA	AB	AB	AA	AA	AB	AB	AA	AB	AA	AA	AB	AB	AA	AB	AA	AB	AB	AA	AA	AA	AB	AB	AA
AB	AA	AB	AA	AA	AA	AB	AB	AB	AA	AB	AA	AA	AA	AA	AB	AA	AA	AB	AB	AA	AA	AB	AB	AA	AB
AA	AA	AA	AA	AA	AA	AB	AA	AA	AB	AB	AB	AA	AA	AA	AB	AB	AB	AA	AA	AA	AA	AA	AA	AA	AB
AA	AA	AB	AB	AA	AA	AB	AA	AA	AB	AA	AB	AA	AA	AA	AB	AA	AA	AA	AA	AA	AA	AB	AA	AA	AB
AB	AA	AB	AA	AB	AB	AB	AB	AB	AB	AA	AA	AB	AA	AA	AB	AB	AA	AB	AB	AA	AA	AA	AB	AB	AB
AA	AB	AA	AB	AA	AA	AB	AA	AB	AA	AA	AA	AB	AB	AA	AA	AB	AB	AA	AA	AA	AA	AB	AB	AA	AA
AB	AB	AA	AA	AB	AA	AB	AB	AB	AA	AA	AA	AA	AB	AA	AB	AA	AA	AA	AB	AA	AB	AA	AA	AA	AB
AA	AA	AB	AA	AA	AB	AB	AB	AB	AB	AB	AA	AA	AA	AA	AA	AB	AA	AA	AA	AB	AA	AB	AA	AA	AA
AB	AA	AA	AA	AB	AA	AA	AA	AA	AB	AB	AA	AA	AB	AA	AB	AB	AA	AA	AB	AA	AA	AB	AA	AA	AB
AB	AB	AB	AB	AB	AA	AA	AA	AB	AB	AB	AB	AB	AB	AB	AB	AA	AB	AB	AB	AA	AB	AA	AB	AB	AB
AB	AB	AA	AB	AA	AA	-	AB	AB	AB	AB	-	AB	AA	AA	-	AA	AB	AB	AA	-	-	-	AB	AB	AA
AB	AB	AB	AB	AB	AB	AB	AB	AB	AB	AB	AB	AB	AB	AB	AB	AB	AB	AB	AB	AB	AB	AB	AB	AB	AB
AA	AA	AA	AA	AB	AB	AB	AB	AA	AB	AA	AA	AA	AB	AA	AA	AB	AA	AA	AB	AB	AA	AB	AB	AB	AA
AB	AA	AA	AA	AB	AB	AB	AB	AA	AB	AB	AB	AB	AB	AA	AB	AB	AB	AB	AB	AB	AA	AB	AB	AB	AB
AB	AB	AB	AB	AA	AB	AA	AB	AA	AB	AB	AB	AB	AB	AB	AB	AA	AB	AB	AB	AB	AB	AA	AB	AB	AB
AB	AA	AA	AA	AB	AA	-	-	AA	AA	AA	-	AA	AB	-	AB	-	-	AA	AB	-	-	AB	AA	-	-
AB	AB	AB	AB	AA	AB	AA	AB	AA	AB	AB	AB	AB	AB	AB	AB	AA	AB	AB	AB	AB	AB	AA	AB	AB	AB

snp_B3085	snp_B3086	snp_B3092	snp_B3098	snp_B3111	snp_B3159	snp_B3162	snp_B3178	snp_B3179	snp_B3181	snp_B3202	snp_B3206	snp_B3244	snp_B3306	snp_B3319	snp_B3330	snp_B3380	snp_B3389	snp_B3393	snp_B3447	snp_B3470	snp_B3476	snp_B3499	snp_B3503	snp_B3522	snp_B3528	snp_B3536	snp_B3542
1	1	1	1	1	1	1	1	1	1	1	1	1	1	1	1	1	1	1	1	1	1	1	1	1	1	1	1
--	--	--	--	LG3	--	--	--	--	--	--	--	--	--	--	--	--	--	--	--	LG3	--	--	LG3	--	LG3	--	--
AB	AB	AA	AA	AB	AB	AA	AB	AA	AB	AB	AB	AA	AB	AA	AA	AA	AA	AB	AA	AB	AB	AA	AA	AA	AB	AA	AA
AB	AB	AB	AB	AB	AB	AB	AB	AB	AB	AB	AB	AB	AB	AB	AB	AB	AB	AB	AB	AB	AB	AB	AB	AB	AB	AB	AB
AB	AA	AA	AB	AA	AB	AA	AB	AB	AB	AA	AB	AB	AB	AB	AB	AB	AB	AB	AB	AB	AB	AA	AB	AA	AB	AB	AB
AA	AA	AB	AB	AA	AA	AA	AA	AA	AA	AB	AA	AA	AA	AB	-	-	AA	AB	AB	AB	AB	AA	AA	AA	AA	AB	AB
AB	AB	AA	AB	AB	AA	AB	AB	AB	AB	AA	AB	AA	AB	AA	AB	AB	AA	AB	AA	AA	AA	AB	AB	AB	AA	AA	AA
AA	AA	AA	AA	AA	-	-	AA	AA	AA	-	AA	AB	AA	AA	AA	-	AA	AA	AA	AA	AA	AA	AA	AB	AA	-	-
AA	AA	AB	AA	AA	AB	-	AA	AA	AA	AB	AA	AB	AB	AB	AA	AB	AB	AA	AB	AB	AB	AB	AB	AA	AA	AA	AB
AB	AB	AB	AA	AB	AB	AB	AB	AB	AB	AA	AA	AA	AB	AA	AB	AA	AB	AA	AB	AA	AB	AA	AB	AB	AB	AB	AA
AB	AA	AB	AA	AB	AB	AA	AA	AB	AA	AB	AA	AA	AB	AA	AA	AA	AB	AA	AA	AB	AB	AB	AA	AB	AB	AA	AA
AA	AA	AB	AA	AA	AB	AA	AA	AA	AA	AB	AA	AA	AB	AA	AA	AA	AB	AA	AA	AB	AB	AA	AB	AA	AB	AA	AB
AA	AA	AB	AB	AA	AA	AB	AA	AA	AB	AB	AB	AA	AB	AB	AA	AB	AB	AA	AA	AB	AB	AA	AA	AB	AA	AA	AB
AB	AB	AA	AB	AA	AB	AB	AB	AA	AB	AB	AB	AA	AA	AA	AB	AA	AA	AA	AB	AB	AB	AB	AB	AA	AB	AB	AA
AA	AA	AA	AB	AB	AB	AB	AB	AA	AA	AB	AB	AA	AA	AA	AA	AA	AB	AA	AA	AB	AA	AA	AB	AA	AB	AB	AA
AA	AA	AB	AB	AA	AA	AA	AA	AA	AA	AB	AA	AA	AB	AA	AB	AA	AB	AA	AB	AB	AA	AA	AB	AA	AA	AB	AB
AA	AA	AB	AB	AA	AB	AB	AA	AB	AA	AB	AA	AA	AB	AA	AA	AA	AB	AA	AA	AB	AB	AB	AB	AA	AA	AA	AB
AA	AA	AB	AB	AA	AB	AB	AA	AB	AA	AB	AA	AA	AB	AB	AB	AB	AB	AA	AA	AB	AB	AB	AB	AA	AB	AB	AB
AB	AB	AA	AA	AB	AB	AB	AB	AB	AB	AA	AB	AA	AB	AB	AB	AA	AB	AB	AA	AB	AB	AA	AA	AB	AB	AB	AB
-	AB	AA	AA	AB	-	-	AB	AB	-	-	AB	AB	AA	AB	AA	-	-	AB	AA	AA	AA	AA	AA	AA	AA	AA	-
AB	AB	AB	AB	AB	AB	AB	AB	AA	AB	AB	AB	AB	AB	AB	AB	AB	AB	AB	AB	AB	AB	AB	AB	AB	AB	AB	AB
AB	AB	AB	AB	AA	AA	-	AB	AA	AB	AA	AB	AB	AA	AA	AB	AB	AA	AA	AB	AA	AA	-	AB	AA	AA	AB	AA
AB	AB	AB	AB	AA	AB	AB	AB	AA	AB	AB	AB	AA	AB	AB	AB	AA	AB	AA	AB	AB	AB	AB	AB	AA	AB	AB	AB
AB	AB	AA	AB	AB	AB	AB	AB	AA	AA	AB	AB	AA	AB	AB	AA	AA	AB	AB	AB	AB	AB	AB	AB	AB	AB	AA	AB
-	AA	-	AA	AA	-	-	AB	AA	AA	-	AA	AA	AA	AB	-	-	-	AA	AB	AB	-	AA	-	AA	AB	AB	-
AB	AB	AB	AA	AB	AB	AB	AB	AB	AA	AB	AB	AA	AB	AB	AA	AA	AB	AB	AA	AB	AB	AA	AB	AB	AB	AA	AB

snp_B3546	snp_B3549	snp_B3564	snp_B3584	snp_B3610	snp_B3651	snp_B3658	snp_B3660	snp_B3663	snp_B3677	snp_B3680	snp_B3686	snp_B3689	snp_B3697	snp_B3709	snp_B3710	snp_B3732	snp_B3736	snp_B3740	snp_B3747	snp_B3748	snp_B3758	snp_B3784
1	1	1	1	1	1	1	1	1	1	1	1	1	1	1	1	1	1	1	1	1	1	1
--	--	--	LG3	LG3	LG3	--	--	--	--	--	--	--	--	--	--	--	--	--	--	--	--	--
AB	AA	AA	AA	AA	AB	AB	AA	AB	AA	AB	AB	AA	AA	AB	AA	AB	AB	AA	AB	AA	AA	AA
AB	AB	AB	AB	AB	AB	AB	AB	AB	AB	AB	AB	AB	AB	AB	AB	AB	AB	AB	AB	AB	AB	AB
AB	AA	AA	AA	AA	AA	AB	AA	AA	AB	AB	AA	AB	AA	AB	AB	AB	AA	AA	AB	AB	AA	AB
AA	AA	AA	AA	AA	AA	AB	AA	AA	AB	AB	AA	AB	-	AA	AB	AA	AB	AA	AB	AB	AA	AA
AA	AA	AB	AB	AB	AA	AB	AB	AB	AA	AA	AB	AA	AA	AA	AB	AA	AA	AB	AB	AA	AB	AA
AB	-	-	AA	AA	-	AA	AB	AA	AA	AA	-	-	AB	AB	AB	AA	AB	AA	AA	AA	AA	AB
AA	AA	AB	AA	AA	AA	AB	AB	AA	AB	AA	AA	AB	AA	AA	AA	AB	AB	AB	AA	AB	AA	AB
AB	AB	AA	AB	AB	AB	AB	AA	AB	AB	AA	AB	AB	AA	AB	AB	AB	AB	AB	AA	AA	AB	AB
AB	AB	AB	AA	AB	AB	AB	AA	AB	AA	AB	AB	AA	AA	AA	AA	AB	AB	AA	AB	AB	AA	AA
AB	AA	AA	AA	AA	AA	AB	AA	AA	AB	AB	AA	AB	AA	AA	AB	AB	AB	AA	AA	AA	AA	AB
AB	AB	AA	AB	AA	AB	AB	AB	AA	AA	AB	AB	AB	AA	AA	AA	AA	AB	AB	AB	AB	AA	AA
AA	AB	AA	AA	AA	AB	AB	AA	AA	AB	AB	AB	AB	AA	AA	AB	AA	AB	AB	AA	AB	AA	AB
AA	AB	AA	AB	AB	AB	AA	AA	AB	AA	AA	AA	AA	AA	AA	AA	AB	AB	AA	AB	AA	AA	AA
AA	AB	AB	AB	AA	AB	AA	AB	AA	AB	AA	AA	AA	AA	AA	AB	AA	AA	AA	AB	AB	AA	AB
AB	AA	AA	AB	AA	AA	AB	AB	AA	AB	AA	AA	AA	AB	AB	AA	AA	AA	AB	AA	AB	AA	AB
AB	AA	AB	AA	AA	AB	AB	AA	AA	AA	AB	AA	AB	AA	AA	AB	AB	AB	AB	AA	AA	AB	AB
AA	AB	AB	AB	AB	AB	AB	AA	AB	AA	AB	AB	AA	AA	AB	AB	AB	AA	AA	AB	AA	AB	AA
AA	-	AB	AA	-	-	AB	AA	AB	AA	AA	-	-	-	AB	AA	AA	AA	-	AA	-	AB	AA
AB	AB	AB	AB	AB	AB	AB	AB	AB	AB	AB	AB	AB	AB	AB	AB	AB	AB	AB	AB	AB	AB	AB
AB	AA	AA	AA	AA	AA	AA	AB	AA	AB	AA	AB	AB	AB	AB	AB	AA	AB	AB	AA	AA	AA	AB
AB	AB	AA	AB	AA	AB	AB	AA	AA	AB	AB	AB	AB	AA	AA	AB	AB	AA	AB	AA	AA	AA	AA
AA	AB	AB	AB	AB	AB	AA	AA	AB	AB	AB	AB	AA	AA	AA	AB	AB	AA	AB	AA	AB	AB	AB
AA	AA	-	AA	AA	-	AB	AB	AA	AB	AB	-	-	-	AA	AA	AB	AB	AB	AB	AA	AA	AB
AA	AB	AB	AB	AB	AB	AB	AA	AB	AB	AB	AB	AB	AA	AA	AB	AB	AA	AB	AB	AB	AB	AB
AA	AB	AB	AA	AA	AB	AA	AA	AA	AB	AA	AB	AA	AA	AA	AB	AA	AB	AB	AB	AA	AA	AB
AB	AB	AA	AB	AA	AB	AA	AA	AB	AA	AA	AA	AB	AA	AB	AA	AA	AB	AB	AA	AA	AA	AB
AB	AA	AB	AA	AA	AB	AB	AB	AA	AA	AA	AA	AB	AB	AA	AB	AA	AB	AA	AA	AB	AA	AB
AB	AA	AA	AB	AA	AB	AB	AA	AA	AA	AA	AA	AB	AB	AA	AB	AA	AB	AA	AA	AB	AA	AB
AB	AA	AB	AB	AA	AB	AA	AA	AA	AB	AB	-	AB	AB	AA	AB	AB	AB	AB	AA	AB	AA	AB
AB	-	AB	AB	AA	-	AA	AA	AB	AB	AA	-	-	AA	AB	AB	AB	AA	-	AB	AB	AB	AB
AA	AB	AB	AB	AB	AB	AA	AA	AB	AA	AB	AA	AB	AA	AB	AA	AA	AA	AA	AB	AA	AB	AB
AA	AB	AA	AB	AB	AB	AA	AA	AB	AA	AA	AB	AB	AA	AB	AA	AA	AA	AB	AB	AA	AB	AB
AB	AA	-	AA	AA	AA	AA	AB	AA	AA	AA	-	AB	-	AA	AA	AA	-	AA	AB	AA	AA	AA
AB	-	AB	AB	AB	AB	AB	AB	AB	AA	AB	-	AB	AB	AA	AB	AB	-	AB	AB	AB	AB	AB
AB	-	-	AA	AA	AA	AA	AA	AA	-	AA	-	-	-	AB	AA	AA	AB	AB	AA	AA	AA	AA
AB	AB	AB	AB	AB	AB	AB	AB	AB	AB	AB	AA	AA	AB	AB	AB	AA	AB	AB	AB	AB	AB	AB
AB	AB	AB	AB	AB	AB	AB	AB	AB	AB	AB	AB	AA	AB	AB	AA	AB	AB	AB	AB	AB	AB	AA
AB	AA	AB	AA	AA	AA	AB	AB	AA	AB	AB	AA	AB	AB	AB	AA	AB	AB	AB	AA	AB	AB	AB
AB	AB	AB	AB	AB	AB	AB	AB	AB	AB	AB	AA	AA	AB	AB	AA	AB	AB	AB	AB	AB	AB	AA
AA	AB	AA	AA	AB	AA	AB	AB	AB	AA	AA	-	-	-	AA	AB	AA	AB	AB	AB	AB	AA	AA
AA	AA	AA	AA	AA	AA	AA	AA	AA	AA	AA	AB	AB	AA	AA	AB	AA	AA	AB	AA	AA	AA	AB
AB	AB	AB	AB	AB	AB	AB	AA	AB	AB	AB	AB	AA	AB	AB	AB	AB	AB	AB	AB	AB	AB	AA
AA	AA	AA	AA	AB	AA	AB	AA	AA	AB	AA	AA	AB	AA	AB	AA	AA	AA	AB	AB	AB	AA	AB
AA	AA	AB	AA	AA	AA	AB	AA	AA	AB	AB	AB	AA	AA	AA	AB	AB	AB	AB	AA	AA	AA	AB
AA	AA	AA	AB	AB	AB	AA	AA	AA	AB	AA	AB	AB	AA	AB	AB	AB	AA	AA	AB	AB	AB	AB
AB	AA	AA	AA	AA	AB	AA	AB	AB	AA	AA	AA	AB	AA	AB	AB	AA	AB	AB	AA	AB	AA	AB
AA	AB	AB	AA	AB	AB	AA	AB	AB	AA	AB	AA	AB	AA	AB	AB	AA	AA	AA	AA	AA	AB	AA
AA	AB	AA	AA	AB	AB	AA	AA	AB	AA	AB	AB	AB	AA	AA	AA	AA	AB	AA	AB	AB	AA	AA
AA	AA	AB	AA	AA	AB	AB	AA	AB	AA	AB	AA	AB	AA	AB	AA	AB	AB	AA	AA	AB	AA	AB
AB	AA	AB	AA	AA	AB	AA	AB	AB	AA	AA	AB	AB	AA	AB	AA	AB	AB	AB	AA	AA	AB	AB

snp_B4512	snp_B4520	snp_B4530	snp_B4538	snp_B4548	snp_B4563	snp_B4568	snp_B4577	snp_B4583	snp_B4590	snp_B4602	snp_B4609	snp_B4624	snp_B4646	snp_B4653	snp_B4671	snp_B4683	snp_B4697	snp_B4706	snp_B4732	snp_B4756	snp_B4763	snp_B4785	snp_B4790	snp_B4793	snp_B4808	snp_B4819	snp_B4848	snp_B4868	snp_B4871	snp_B4880	snp_B4887	snp_B4909	snp_B4914	snp_B4924	snp_B4948	snp_B4972	snp_B4981	snp_B4992	snp_B5016	snp_B5079	snp_B5111	snp_B5131	snp_B5141	snp_B5145	snp_B5170	snp_B5179	snp_B5209	snp_B5215	snp_B5231	snp_B5235	snp_B5254	snp_B5269	snp_B5278	snp_B5304	snp_B5323	snp_B5336	snp_B5344	snp_B5418	snp_B5464	snp_B5466	snp_B5478	snp_B5486	snp_B5530	snp_B5544	snp_B5584	snp_B5585	snp_B5592	snp_B5597	snp_B5599	snp_B5610	snp_B5616	snp_B5618	snp_B5659	snp_B5684	snp_B5701	snp_B5710
1	1	1	1	1	1	1	1	1	1	1	1	1	1	1	1	1	1	1	1	1	1	1	1	1	1	1	1	1	1	1	1	1	1	1	1	1	1	1	1	1	1	1	1	1	1	1	1	1	1	1	1	1	1	1	1	1	1	1	1	1	1	1	1	1	1	1	1	1	1	1	1	1	1	1	1	1
--	--	LG3	LG3	--	--	--	--	--	--	--	--	--	--	--	--	--	--	--	--	--	--	--	--	--	--	--	--	--	--	--	LG3	--	--	--	--	--	LG3	--	--	--	--	--	--	--	--	--	--	--	--	--	--	LG3	--	LG3	--	--	--	LG3	--	--	--	--	--	--	--	--	--	--	--	LG3	--	--	--	LG3	--	--
AA	AB	AB	AA	AA	AB	AB	AA	AB	AA	AA	AB	AA	AA	AA	AA	AB	AB	AA	AB	AA	AB	AA	AA	AA	AA	AB	AB	AB	AB	AB	AA	AA	AB	AB	AB	AB	AB	AB	AA	AA	AB	AA	AA	AB	AA	AB	AA	AA	AB	AA	AA	AA	AB	AB	AA	AA	AB	AA	AB	AA	AB	AB	AA	AB	AA	AB	AA	AB	AB	AB	AB	AA	AA	AB	AB	AB

snp_B5746	snp_B5755	snp_B5785	snp_B5787	snp_B5803	snp_B5833	snp_B5842	snp_B5844	snp_B5847	snp_B5867	snp_B5876	snp_B5880	snp_B5881	snp_B5882	snp_B5941	snp_B5943	snp_B5945	snp_B5962	snp_B5981	snp_B6015	snp_B6027
1	1	1	1	1	1	1	1	1	1	1	1	1	1	1	1	1	1	1	1	1
--	--	LG3	--	--	--	--	--	--	--	LG3	--	--	--	LG3	--	--	--	--	--	--
[illegible]	[illegible]	[illegible]	[illegible]	[illegible]	[illegible]	[illegible]	[illegible]	[illegible]	[illegible]	[illegible]	[illegible]	[illegible]	[illegible]	[illegible]	[illegible]	[illegible]	[illegible]	[illegible]	[illegible]	[illegible]

snp_B6151	snp_B6153	snp_B6166	snp_B6208	snp_B6243	snp_B6360	snp_B0026	snp_B0042	snp_B0055	snp_B0066	snp_B0071	snp_B0074	snp_B0104	snp_B0135	snp_B0157	snp_B0183	snp_B0207	snp_B0208	snp_B0258
1	1	1	1	1	1	2	2	2	2	2	2	2	2	2	2	2	2	2
--	--	--	--	--	--	--	--	--	--	--	--	LG1	--	--	LG1	--	--	--
[illegible]	[illegible]	[illegible]	[illegible]	[illegible]	[illegible]	[illegible]	[illegible]	[illegible]	[illegible]	[illegible]	[illegible]	[illegible]	[illegible]	[illegible]	[illegible]	[illegible]	[illegible]	[illegible]

snp_B0270	snp_B0272	snp_B0274	snp_B0280	snp_B0309	snp_B0315	snp_B0332	snp_B0341	snp_B0366	snp_B0375	snp_B0379	snp_B0395	snp_B0400	snp_B0421	snp_B0451	snp_B0460	snp_B0472	snp_B0479	snp_B0496
2	2	2	2	2	2	2	2	2	2	2	2	2	2	2	2	2	2	2
--	--	--	--	--	--	--	--	--	--	--	--	--	--	--	--	--	--	--
[illegible]	[illegible]	[illegible]	[illegible]	[illegible]	[illegible]	[illegible]	[illegible]	[illegible]	[illegible]	[illegible]	[illegible]	[illegible]	[illegible]	[illegible]	[illegible]	[illegible]	[illegible]	[illegible]

snp_B0507	snp_B0534	snp_B0549	snp_B0564	snp_B0594	snp_B0608	snp_B0639	snp_B0642	snp_B0652	snp_B0660	snp_B0681	snp_B0703	snp_B0716	snp_B0775	snp_B0780	snp_B0795	snp_B0807	snp_B0825
2	2	2	2	2	2	2	2	2	2	2	2	2	2	2	2	2	2
--	--	--	--	--	--	--	--	--	--	--	--	LG1	--	--	--	LG1	--
[illegible]	[illegible]	[illegible]	[illegible]	[illegible]	[illegible]	[illegible]	[illegible]	[illegible]	[illegible]	[illegible]	[illegible]	[illegible]	[illegible]	[illegible]	[illegible]	[illegible]	[illegible]

snp_B0826	snp_B0859	snp_B0881	snp_B0891	snp_B0897	snp_B0904	snp_B0925	snp_B0929	snp_B0953	snp_B0965	snp_B0972	snp_B0973	snp_B0974	snp_B0983	snp_B0988	snp_B0998	snp_B1001	snp_B1010	snp_B1021	snp_B1022	snp_B1023	snp_B1027	snp_B1028	snp_B1030	snp_B1067	snp_B1071	snp_B1079	snp_B1086	snp_B1135	snp_B1166	snp_B1168	snp_B1173	snp_B1178	snp_B1201	snp_B1207	snp_B1210	snp_B1285	snp_B1290	snp_B1305	snp_B1311	snp_B1321	snp_B1327	snp_B1337	snp_B1341	snp_B1355	snp_B1364	snp_B1370	snp_B1393	snp_B1412	snp_B1418	snp_B1422	snp_B1441	snp_B1459	snp_B1466	snp_B1471	snp_B1472	snp_B1489	snp_B1516	snp_B1552	snp_B1559	snp_B1562	snp_B1563	snp_B1622	snp_B1624	snp_B1626	snp_B1636	snp_B1640	snp_B1659	snp_B1687	snp_B1709	snp_B1713	snp_B1724	snp_B1742	snp_B1746	snp_B1756	snp_B1777	snp_B1801
2	2	2	2	2	2	2	2	2	2	2	2	2	2	2	2	2	2	2	2	2	2	2	2	2	2	2	2	2	2	2	2	2	2	2	2	2	2	2	2	2	2	2	2	2	2	2	2	2	2	2	2	2	2	2	2	2	2	2	2	2	2	2	2	2	2	2	2	2	2	2	2	2	2	2	2	2
--	--	--	--	--	--	--	--	--	--	--	--	--	--	--	--	LG1	--	--	--	--	LG1	--	LG1	--	--	--	--	LG1	--	--	--	--	LG1	LG1	--	--	--	--	--	--	--	--	--	--	--	--	--	--	--	--	--	--	--	--	--	--	--	--	LG1	LG1	--	--	LG1	--	--	--	--	--	--	--	--	--	--	--	--	--

snp_B1803	snp_B1809	snp_B1816	snp_B1862	snp_B1865	snp_B1867	snp_B1887	snp_B1897	snp_B1917	snp_B1919	snp_B1924	snp_B1931	snp_B1935	snp_B1948	snp_B1952	snp_B1955	snp_B1967	snp_B1984	snp_B1990	snp_B2005	snp_B2006	snp_B2035	snp_B2036
2	2	2	2	2	2	2	2	2	2	2	2	2	2	2	2	2	2	2	2	2	2	2
--	--	--	--	LG1	--	--	--	--	--	--	--	--	LG1	--	--	--	--	--	LG1	--	--	--
AB	AB	AB	AB	AA	AB	AB	AB	AB	AB	AB	AB	AB	AA	AB	AA	AB	AA	AB	AA	AB	AA	AB
AA	AA	AA	AA	AA	AB	AA	AA	AB	AA	AA	AA	AA	AB	AA	AA	AB	AB	AA	AA	AA	AB	AA
AA	AA	AA	AB	AA	AB	AA	AA	AA	AB	AA	AB	AB	AB	AA	AB	AA	AB	AA	AA	AA	AA	AB
AA	AB	AA	AB	-	AA	AA	AA	AA	AA	AA	AA	AB	AA	AA	AA	AA	-	AA	AB	AA	AA	AB
AB	AB	AB	AB	AB	AB	AA	AB	AB	AA	AA	AB	AB	AB	AA	AB	AB	AB	AB	AA	AB	AB	AA
AA	AA	AA	AA	-	-	AA	AA	AA	AA	AA	AA	-	AA	AA	AA	AA	-	-	AA	AA	-	AA
AB	AB	AB	AA	AB	AB	AB	AA	AB	AA	AA	AA	AA	AA	AB	AA	AA	AB	AB	AB	AB	AB	AB
AA	AA	AB	AA	AA	AB	AB	AA	AA	AB	AB	AA	AA	AB	AB	AB	AB	AA	AB	AA	AA	AA	AA
AA	AA	AB	AA	AA	AA	AB	AA	AA	AB	AB	AA	AA	AB	AB	AB	AA	AB	AA	AA	AA	AB	AA
AA	AA	AB	AA	AA	AA	AA	AB	AA	AA	AB	AA	AA	AB	AA	AB	AA	AA	AB	AA	AA	AB	AA
AA	AA	AB	AA	AA	AB	AB	AA	AA	AB	AB	AB	AB	AA	AB	AB	AA	AA	AB	AA	AA	AB	AB
AA	AA	AB	AA	AA	AA	AA	AA	AA	AB	AA	AA	AA	AA	AA	AB	AA	AB	AA	AA	AA	AA	AB
AB	AB	AA	AB	AA	AA	AA	AB	AA	AA	AA	AB	AB	AA	AA	AA	AB	AA	AB	AA	AB	AA	AA
AB	AA	AB	AA	AB	AB	AA	AA	AB	AA	AA	AB	AA	AA	AA	AA	AB	AB	AB	AA	AA	AA	AB
AA	AA	AB	AB	AA	AB	AB	AB	AA	AB	AB	AB	AB	AB	AB	AB	AA	AB	AB	AA	AA	AB	AA
AB	AA	AB	AA	AA	AB	AA	AB	AA	AA	AB	AA	AA	AA	AA	AB	AA	AA	AB	AA	AB	AB	AA
AB	AB	AB	AB	AB	AB	AA	AB	AB	AB	AB	AB	AB	AB	AA	AB	AB	AB	AB	AB	AB	AB	AB
AB	AB	AA	AB	AB	-	AA	AB	AB	AA	AA	-	-	AA	-	AA	AB	-	-	AB	AB	-	-
AB	AB	AB	AB	AB	AB	AB	AB	AB	AB	AB	AB	AB	AB	AB	AB	AB	AB	AB	AB	AB	AB	AB
AB	AB	AB	AB	AB	AB	AB	AA	AB	AB	AB	AB	AB	AB	AA	AB	AB	AB	AB	AB	AB	AB	AB
AA	AA	AB	AA	AA	AA	AB	AA	AB	AB	AB	AA	AB	AB	AB	AB	AA	AA	AB	AA	AA	AB	AA
AA	AB	AB	AB	AB	AA	AB	AA	AA	AB	AA	AA	AB	AA	AB	AA	AA	AB	AA	AB	AA	AA	AB
AB	AB	AA	-	-	AB	AA	AB	-	AA	AB	-	AA	AB	-	-	AA	-	-	AB	AB	AB	-
AA	AA	AA	AA	AA	AA	AA	AA	AA	AA	AA	AA	AA	AA	AA	AA	AA	AA	AA	AA	AA	AA	AA
AB	AA	AA	AA	AA	AB	AB	AB	AA	AA	AA	AB	AA	AB	AB	AA	AA	AA	AB	AB	AB	AB	AB
AA	AA	AA	AA	AA	AB	AA	AA	AA	AA	AA	AA	AA	AA	AA	AA	AA	AA	AA	AA	AA	AA	AA
AA	AB	AA	AA	AB	AA	AB	AA	AA	AB	AA	AA	AA	AA	AB	AA	AA	AB	AA	AA	AA	AA	AB
AB	AA	AA	AB	AA	AB	AB	AA	AA	AB	AB	AB	AB	AB	AB	AB	AB	AA	AA	AA	AA	AB	AA
AA	AA	AA	AB	AA	AB	AB	AA	AA	AB	AA	AB	AB	AB	AB	AB	AB	AB	AA	AA	AA	AB	AA
AB	AB	AB	AB	-	-	AB	-	AB	AA	AB	-	AB	-	-	-	AB	-	AB	AA	AB	-	AA
AB	AB	AB	AA	AB	AB	AA	AA	AB	AA	AA	AA	AA	AA	AA	AA	AA	AB	AB	AB	AB	AB	AB
AA	AB	AA	AB	AA	AB	AB	AB	AA	AA	AA	AB	AB	AB	AB	AA	AB	AA	AA	AA	AA	AA	AA
AA	AB	AA	-	-	AB	AA	AA	AA	AA	AB	AA	AB	AA	AA	AB	AA	-	-	AA	AA	AA	AA
AB	AB	AB	-	AA	AB	AA	AB	-	AB	AB	AB	AB	AB	AB	AB	AB	AA	-	AA	AB	AB	AA
AA	AB	AA	AB	-	AA	AA	AA	AB	AA	AA	-	-	AB	-	AA	AB	AB	AA	AB	-	AA	AB
AB	AB	AA	AB	AA	AB	AA	AB	AB	AA	AA	AB	AB	AB	AA	AA	AB	AB	-	AA	AB	AB	AA
AA	AA	AA	AA	AA	AA	AB	AA	AA	AA	AA	AA	AA	AA	AB	AA	AA	AA	AA	AA	AA	AA	AA
AB	AA	AA	AB	AA	AB	AB	AB	AB	AB	AB	AB	AB	AB	AB	AB	AB	AA	AB	AA	AB	AA	AA
AB	AB	AB	AB	AB	AB	AB	AB	AB	AB	AB	AB	AB	AB	AB	AB	AB	AB	AB	AB	AB	AB	AB
AB	AA	AA	AA	AA	AA	AB	AB	AB	AB	-	-	AA	AB	AB	AA	AA	-	-	AB	AB	AA	AB
AB	AA	AB	AA	AA	AB	AB	AB	AA	AB	AB	AA	AA	AA	AB	AB	AA	AA	AB	AA	AB	AB	AA
AA	AA	AA	AB	AA	AA	AB	AB	AA	AB	AB	AB	AB	AB	AB	AB	AB	AA	AA	AA	AA	AA	AA
AB	AA	AA	AA	AA	AA	AB	AA	AB	AA	AA	AA	AA	AA	AB	AA	AA	AA	AB	AA	AB	AB	AA
AB	AB	AA	AB	-	AB	AA	AB	-	AA	AA	AB	AB	AB	AA	AB	AB	AB	AB	AA	AB	AB	AA
AB	AA	AA	AA	AB	AA	AA	AA	AA	AA	AA	AB	AB	AA	AB	AA	AA	AA	AA	AB	AA	AA	AA
AB	AB	AA	AB	AA	AB	AB	AB	AB	AB	AB	AB	AB	AB	AB	AB	AB	AB	AB	AA	AB	AB	AA
AB	AB	AB	AB	AB	AB	AB	AB	AB	AB	AB	AB	AB	AB	AB	AB	AB	AB	AB	AB	AB	AB	AB
AB	AB	AB	AB	AB	AB	AB	AB	AA	AB	AB	AB	AA	AB	AB	AB	AA	AA	AB	AB	AB	AB	AB
AB	AB	AA	AB	AB	AB	AA	AB	AA	AB	AB	AB	AB	AB	AB	AB	AA	AB	AB	AB	AB	AB	AB
AB	AA	AB	AA	AA	AB	AB	AB	AA	AB	AB	AB	AB	AB	AB	AB	AB	AA	AB	AB	AB	AB	AA
AA	AA	AA	AA	AA	AA	AA	AA	AA	AA	AA	AA	AA	AA	AA	AA	AA	AA	AA	AB	AA	AA	AA
AA	AA	AA	AA	AB	AA	AB	AB	AA	AB	AB	AA	AA	AA	AB	AB	AB	-	AB	AA	AA	AA	AA
AB	AB	AB	AB	AB	AB	AB	AB	AB	AA	AA	AB	AB	AB	AB	AB	AB	AB	AB	AB	AB	AB	AB
AA	AB	AB	AA	AB	AB	AB	AA	AA	AA	AA	AA	AA	AA	AA	AA	AA	AB	AA	AB	AA	AB	AB
[illegible]	[illegible]	[illegible]	[illegible]	[illegible]	[illegible]	[illegible]	[illegible]	[illegible]	[illegible]	[illegible]	[illegible]	[illegible]	[illegible]	[illegible]	[illegible]	[illegible]	[illegible]	[illegible]	[illegible]	[illegible]	[illegible]	[illegible]

snp_B2048	snp_B2056	snp_B2065	snp_B2077	snp_B2123	snp_B2130	snp_B2132	snp_B2144	snp_B2166	snp_B2180	snp_B2193	snp_B2203	snp_B2205	snp_B2211	snp_B2242	snp_B2247	snp_B2261	snp_B2276	snp_B2311	snp_B2348	snp_B2359	snp_B2367	snp_B2375	snp_B2424	snp_B2428	snp_B2434	snp_B2471
2	2	2	2	2	2	2	2	2	2	2	2	2	2	2	2	2	2	2	2	2	2	2	2	2	2	2
--	--	--	--	--	--	--	--	--	--	--	--	--	--	--	--	--	--	--	--	--	--	--	--	--	--	--
[illegible]	[illegible]	[illegible]	[illegible]	[illegible]	[illegible]	[illegible]	[illegible]	[illegible]	[illegible]	[illegible]	[illegible]	[illegible]	[illegible]	[illegible]	[illegible]	[illegible]	[illegible]	[illegible]	[illegible]	[illegible]	[illegible]	[illegible]	[illegible]	[illegible]	[illegible]	[illegible]

snp_B2515	snp_B2537	snp_B2542	snp_B2593	snp_B2638	snp_B2649	snp_B2677	snp_B2680	snp_B2683	snp_B2688	snp_B2690	snp_B2692	snp_B2712	snp_B2735	snp_B2765	snp_B2797	snp_B2800	snp_B2802	snp_B2825	snp_B2845	snp_B2846	snp_B2857	snp_B2877	snp_B2882	snp_B2899	snp_B2904	snp_B2906
2	2	2	2	2	2	2	2	2	2	2	2	2	2	2	2	2	2	2	2	2	2	2	2	2	2	2
LG1	--	--	--	--	--	--	LG1	--	--	--	--	--	--	--	--	--	--	--	--	--	--	LG1	--	--	--	--
[illegible]	[illegible]	[illegible]	[illegible]	[illegible]	[illegible]	[illegible]	[illegible]	[illegible]	[illegible]	[illegible]	[illegible]	[illegible]	[illegible]	[illegible]	[illegible]	[illegible]	[illegible]	[illegible]	[illegible]	[illegible]	[illegible]	[illegible]	[illegible]	[illegible]	[illegible]	[illegible]

snp_B2922	snp_B2939	snp_B2957	snp_B3000	snp_B3010	snp_B3016	snp_B3025	snp_B3027	snp_B3049	snp_B3096	snp_B3101	snp_B3115	snp_B3135	snp_B3140	snp_B3144	snp_B3209	snp_B3233
2	2	2	2	2	2	2	2	2	2	2	2	2	2	2	2	2
--	--	--	--	--	--	--	--	--	--	--	--	--	--	--	--	--
AB	AA	AA	AB	AA	AA	AA	AB	AA	AB	AA	AA	AA	AA	AA	AB	AB
AB	AA	AA	AB	AA	AA	AA	AB	AA	AA	AB	AA	AB	AA	AA	AB	AB
AA	AA	AB	AB	AB	AA	AB	AA	AA	AB	AB	AA	AB	AA	AA	AB	AB
AA	AA	AA	AA	-	AA	AB	AA	AA	AB	-	-	AA	AA	AA	AA	AA
AB	AB	AB	AA	AA	AB	AB	AB	AB	AB	AB	AA	AB	AA	AB	AB	AB
-	AA	-	AA	-	AA	-	AA	AA	AA	-	-	AA	AA	AA	AA	AA
AB	AA	AA	AA	AB	AB	AB	AA	AB	AB	AA	AB	AB	AB	AB	AA	AB
AB	AA	AB	AB	AB	AA	AA	AA	AB	AA	AA	AA	AA	AB	AA	AB	AA
AA	AA	AB	AA	AA	AB	AB	AB	AA	AA	AB	AA	AB	AA	AB	AB	AA
AA	AB	AB	AB	AB	AB	AB	AB	AB	AA	AB	AA	AB	AB	AB	AB	AB
AA	AA	AB	AB	AA	AA	AB	AB	AB	AA	AB	AA	AB	AB	AA	AB	AA
AA	AB	AB	AB	AB	AA	AB	AA	AB	AA	AA	AA	AB	AA	AA	AB	AA
AB	AA	AA	AA	AA	AB	AA	AA	AA	AB	AA	AA	AA	AA	AA	AA	AA
AB	AA	AA	AB	AA	AB	AB	AA	AB	AB	AA	AA	AA	AA	AA	AA	AB
AA	AA	AB	AB	AA	AB	AA	AB	AB	AA	AB	AA	AA	AA	AA	AB	AA
AA	AB	AA	AB	AB	AA	AB	AB	AA	AA	AB	AA	AB	AA	AB	AB	AA
AB	AB	AB	AB	AB	AB	AB	AB	AA	AB	AA	AB	AB	AA	AB	AB	AB
AB	AA	AA	AA	-	-	AA	AB	AB	AB	AB	-	AA	AB	AB	AA	AB
AB	AB	AB	AB	AB	AB	AB	AB	AB	AB	AB	AB	AB	AB	AB	AB	AB
AB	AB	AB	AB	-	AB	AB	AB	AB	AB	AB	AB	AB	AB	AB	AB	AB
AA	AB	AB	AB	AB	AB	AB	AB	AB	AA	AB	AA	AB	AB	AB	AB	AB
AA	AB	AA	AA	AA	AA	AB	AA	AA	AA	AB	AB	AA	AA	AA	AB	AA
AB	AA	-	AB	-	-	AA	-	AA	AB	-	-	AA	AA	AA	AA	AA
AA	AA	AA	AA	AA	AA	AA	AA	AA	AA	AB	AA	AA	AA	AA	AA	AA
AB	AB	AA	AA	AA	AA	AA	AA	AA	AB	AA	AB	AA	AA	AA	AA	AB
AA	AA	AA	AA	AA	AA	AA	AA	AA	AA	AB	AA	AA	AA	AA	AA	AA
AA	AA	AA	AA	AB	AA	AA	AA	AA	AB	AA	AA	AA	AA	AA	AA	AA
AB	AB	AB	AB	AB	AA	AB	AA	AA	AA	AB	AA	AB	AA	AB	AB	AB
AA	AA	-	AB	AB	AB	-	AB	AA	AB	-	AA	AA	AA	AA	AB	AA
-	AB	AB	AB	-	-	AB	AB	AA	AB	-	-	AB	AA	AB	AB	AB
AB	AA	AA	AA	AB	AB	AB	AA	AB	AB	AA	AB	AB	AB	AB	AA	AB
AA	AA	AA	AA	AA	AA	AB	AB	AA	AA	AB	AA	AA	AA	AA	AA	AA
AA	AA	AB	AB	-	AA	AB	AA	AB	AA	AB	-	AA	AB	AA	AB	AB
AB	AA	AB	AB	-	-	AA	AB	AA	AB	-	AA	AB	AA	AB	AA	AB
-	AA	AB	-	-	AB	AA	AA	AB	AB	AA	-	AA	AB	AA	AA	AB
AB	AA	AA	AA	AA	AB	AB	AB	AB	AB	AA	AA	AB	AA	AB	AB	AB
AA	AA	AA	AA	AA	AA	AA	AA	AA	AA	AB	AA	AA	AA	AA	AA	AA
AB	AB	AB	AB	AA	AB	AA	AB	AA	AA	AB	AA	AB	AA	AB	AB	AB
AB	AB	AB	AB	AB	AB	AB	AB	AB	AB	AB	AB	AB	AB	AB	AB	AB
AB	AA	AA	AA	-	-	AA	AA	AA	AB	AA	-	AA	AB	AA	AB	AB
AA	AB	AA	AB	AB	AA	AB	AB	AA	AA	AB	AA	AB	AA	AB	AB	AA
AA	AB	AB	AB	AA	AA	AA	AB	AA	AA	AB	AA	AB	AA	AA	AB	AA
AB	AA	AA	AA	AA	AB	AA	AB	AA	AB	AB	AA	AB	AA	AA	AB	AA
AB	AA	-	AA	AA	AB	AB	AB	AA	AB	AB	AA	AB	AA	AB	AB	AB
AA	AA	AA	AA	AB	AA	AA	AA	AB	AB	AB	AB	AB	AB	AA	AA	AB
AB	AB	AB	AB	AB	AB	AB	AB	AA	AB	AB	AA	AB	AA	AB	AB	AB
AB	AB	AB	AB	AB	AB	AB	AB	AB	AB	AB	AB	AB	AB	AB	AB	AB
AB	AB	AA	AB	AB	AB	AA	AB	AB	AB	AA	AB	AB	AB	AA	AA	AB
AB	AB	AB	AB	AB	AB	AB	AB	AB	AB	AB	AB	AB	AB	AB	AB	AB
AA	AB	AA	AB	AB	AA	AB	AB	AA	AB	AB	AA	AB	AA	AB	AB	AA
AA	AA	AA	AA	AA	AA	AA	AA	AA	AA	AA	AB	AA	AA	AA	AA	AA
AB	AB	AB	AB	AB	AB	AB	AB	AB	AB	-	-	AA	AB	AB	AB	AA
AB	AB	AB	AA	AA	AB	AB	AB	AB	AB	AB	AA	AB	AA	AB	AB	AB
[illegible]	[illegible]	[illegible]	[illegible]	[illegible]	[illegible]	[illegible]	[illegible]	[illegible]	[illegible]	[illegible]	[illegible]	[illegible]	[illegible]	[illegible]	[illegible]	[illegible]

snp_B3241	snp_B3288	snp_B3289	snp_B3290	snp_B3305	snp_B3310	snp_B3371	snp_B3372	snp_B3381	snp_B3391	snp_B3404	snp_B3407	snp_B3416	snp_B3421	snp_B3445	snp_B3452	snp_B3480	snp_B3519	snp_B3529
2	2	2	2	2	2	2	2	2	2	2	2	2	2	2	2	2	2	2
--	--	--	--	--	--	--	--	--	--	LG1	--	--	--	--	--	--	--	--
[illegible]	[illegible]	[illegible]	[illegible]	[illegible]	[illegible]	[illegible]	[illegible]	[illegible]	[illegible]	[illegible]	[illegible]	[illegible]	[illegible]	[illegible]	[illegible]	[illegible]	[illegible]	[illegible]

snp_B3533	snp_B3539	snp_B3547	snp_B3555	snp_B3566	snp_B3570	snp_B3572	snp_B3580	snp_B3593	snp_B3603	snp_B3625	snp_B3629	snp_B3630	snp_B3637	snp_B3652	snp_B3685	snp_B3726	snp_B3727	snp_B3755	snp_B3764	snp_B3766	snp_B3769	snp_B3783
2	2	2	2	2	2	2	2	2	2	2	2	2	2	2	2	2	2	2	2	2	2	2
--	--	--	--	LG1	--	--	--	--	--	--	LG1	--	--	--	--	--	--	--	--	--	--	LG1
[illegible]	[illegible]	[illegible]	[illegible]	[illegible]	[illegible]	[illegible]	[illegible]	[illegible]	[illegible]	[illegible]	[illegible]	[illegible]	[illegible]	[illegible]	[illegible]	[illegible]	[illegible]	[illegible]	[illegible]	[illegible]	[illegible]	[illegible]

snp_B3785	snp_B3787	snp_B3818	snp_B3834	snp_B3844	snp_B3863	snp_B3910	snp_B3915	snp_B3920	snp_B3929	snp_B3955	snp_B3968	snp_B3980	snp_B3984	snp_B3997	snp_B4048	snp_B4059	snp_B4082
2	2	2	2	2	2	2	2	2	2	2	2	2	2	2	2	2	2
--	--	--	--	--	--	LG1	--	--	--	LG1	--	--	--	--	--	--	--
[illegible]	[illegible]	[illegible]	[illegible]	[illegible]	[illegible]	[illegible]	[illegible]	[illegible]	[illegible]	[illegible]	[illegible]	[illegible]	[illegible]	[illegible]	[illegible]	[illegible]	[illegible]

snp_B4100	snp_B4101	snp_B4169	snp_B4185	snp_B4192	snp_B4194	snp_B4197	snp_B4199	snp_B4202	snp_B4203	snp_B4214	snp_B4221	snp_B4272	snp_B4281	snp_B4303	snp_B4314	snp_B4323	snp_B4356	snp_B4364	snp_B4415	snp_B4435	snp_B4463	snp_B4480	snp_B4509	snp_B4516	snp_B4527	snp_B4529	snp_B4531
2	2	2	2	2	2	2	2	2	2	2	2	2	2	2	2	2	2	2	2	2	2	2	2	2	2	2	2
--	LG1	--	LG1	--	--	--	--	--	--	--	--	--	--	--	--	--	--	--	--	--	--	--	--	--	--	--	--
AB	AA	AA	AA	AA	AB	AA	AA	AA	AB	AA	AA	AB	AB	AB	AB	AA	AB	AB	AA	AB	AB	AA	AB	AA	AB	AB	AB
AA	AA	AA	AB	AB	AA	AA	AA	AA	AA	AA	AA	AA	AA	AA	AA	AA	AA	AA	AB	AA	AA	AA	AA	AA	AA	AA	AA
AA	AA	AA	AA	AB	AB	AB	AB	AA	AA	AA	AB	AB	AB	AB	AA	AA	AA	AA	AB	AA	AA	AB	AA	AA	AA	AB	AA
AA	AB	AB	AB	AB	AA	AA	-	AB	AB	AB	AB	-	AA	AB	AB	AA	AB	AB	AA	AB	AB	AB	-	AA	AA	AA	-
AA	AB	AB	AA	AB	AB	AB	AA	AB	AB	AB	AA	AB	AA	AB	AA	AB	AB	AB	AB	AB	AB	AB	AB	AA	AB	AB	AA

snp_B4594	snp_B4601	snp_B4614	snp_B4625	snp_B4631	snp_B4640	snp_B4658	snp_B4688	snp_B4727	snp_B4729	snp_B4747	snp_B4765	snp_B4771	snp_B4777	snp_B4778	snp_B4783	snp_B4787	snp_B4799	snp_B4805	snp_B4825	snp_B4827	snp_B4835
2	2	2	2	2	2	2	2	2	2	2	2	2	2	2	2	2	2	2	2	2	2
--	--	--	--	--	--	--	LG1	--	--	LG1	--	LG1	--	--	--	--	--	--	--	--	--
AB	AB	AB	AB	AB	AA	AB	AA	AA	AB	AA	AB	AB	AA	AA	AB	AA	AB	AA	AA	AA	AB
AA	AA	AA	AB	AA	AA	AA	AB	AA	AB	AB	AB	AB	AA	AA	AB	AB	AA	AA	AA	AA	AB
AA	AB	AB	AB	AB	AB	AA	AB	AA	AA	AA	AA	AA	AA	AA	AB	AB	AA	AB	AB	AA	AB
AA	AA	AA	AA	AA	AB	AB	AA	AB	AB	AB	AA	AB	AA	AB	AA	-	AB	AB	AA	AB	AA
AB	AB	AB	AB	AB	AA	AB	AB	AB	AA	AA	AB	AA	AA	AB	AB	AB	AA	AA	AA	AB	AB

snp_B4836	snp_B4855	snp_B4867	snp_B4869	snp_B4885	snp_B4898	snp_B4903	snp_B4908	snp_B4942	snp_B4957	snp_B4967	snp_B4996	snp_B4999	snp_B5012	snp_B5027	snp_B5116	snp_B5139	snp_B5140	snp_B5159	snp_B5168	snp_B5185	snp_B5206	snp_B5223	snp_B5233	snp_B5267	snp_B5272	snp_B5286
2	2	2	2	2	2	2	2	2	2	2	2	2	2	2	2	2	2	2	2	2	2	2	2	2	2	2
--	--	LG1	--	--	--	--	--	--	--	--	--	--	--	--	--	--	--	--	--	--	--	--	--	--	--	--
AB	AB	AA	AB	AB	AB	AA	AB	AA	AB	AB	AA	AB	AA	AB	AA	AA	AB	AB	AA	AB	AA	AB	AB	AB	AB	AA
AA	AB	AB	AA	AA	AA	AA	AA	AA	AB	AB	AA	AA	AA	AB	AA	AA	AA	AB	AB	AB	AA	AB	AA	AB	AB	AA
AB	AA	AB	AA	AA	AA	AA	AB	AB	AA	AA	AA	AA	AB	AA	AB	AA	AA	AA	AB	AB	AA	AB	AA	AA	AB	AB
AA	AA	AA	AA	AA	AB	AB	AB	AB	AB	AA	AA	AA	AA	AB	AB	AB	AA	AA	AA	AA	AA	AB	AB	AA	AA	-
AA	AB	AB	AB	AB	AB	AB	AB	AB	AB	AB	AA	AB	AB	AA	AA	AB	AA	AB	AB	AA	AB	AB	AA	AB	AB	AA

snp_B5289	snp_B5299	snp_B5318	snp_B5325	snp_B5415	snp_B5430	snp_B5451	snp_B5460	snp_B5475	snp_B5502	snp_B5522	snp_B5536	snp_B5545	snp_B5569	snp_B5590	snp_B5604	snp_B5614
2	2	2	2	2	2	2	2	2	2	2	2	2	2	2	2	2
--	--	--	--	--	--	--	--	--	--	--	--	--	--	--	--	--
AA	AA	AB	AA	AB	AB	AB	AB	AA	AB	AA	AB	AB	AA	AB	AA	AA
AA	AA	AA	AA	AA	AA	AA	AA	AA	AA	AA	AA	AA	AA	AA	AB	AA
AA	AA	AA	AA	AB	AA	AB	AB	AB	AA	AB	AA	AA	AA	AB	AB	AA
AB	AB	AB	AA	AB	AB	AA	AB	AB	AA	AB	AA	AB	AB	AB	AA	AB
AA	AB	AB	AA	AB	AB	AB	AB	AA	AB	AB	AB	AA	AA	AB	AB	AA
AA	-	-	AA	AA	AA	-	AA	AA	AA	AA	AA	AA	AA	AA	AA	AA
AB	AB	AA	AA	AA	AB	AA	AB	AA	AB	AA	AB	AB	AB	AA	AA	AB
AA	AB	AA	AB	AB	AA	AB	AA	AA	AA	AA	AA	AA	AA	AB	AA	AA
AA	AB	AA	AB	AA	AA	AB	AA	AB	AA	AB	AA	AA	AA	AA	AB	AA
AB	AB	AA	AA	AA	AB	AB	AA	AB	AA	AB	AA	AB	AA	AA	AB	AA
AA	AA	AA	AA	AB	AA	AB	AA	AA	AA	AB	AA	AA	AA	AA	AB	AA
AB	AA	AB	AB	AA	AB	AB	AA	AA	AB	AB	AA	AA	AA	AA	AA	AA
AA	AB	AB	AA	AB	AA	AA	AB	AA	AB	AA	AB	AA	AA	AB	AA	AA
AA	AB	AB	AB	AB	AB	AB	AB	AA	AB	AB	AB	AB	AA	AB	AA	AA
AA	AA	AA	AB	AA	AA	AA	AA	AA	AA	AA	AA	AA	AA	AA	AB	AA
AB	AA	AA	AB	AB	AA	AA	AA	AA	AA	AB	AA	AA	AA	AA	AB	AA
AB	AB	AB	AB	AB	AA	AB	AB	AA	AB	AB	AB	AB	AB	AB	AB	AB
AB	-	-	AB	-	AB	-	AB	AA	AA	AA	AB	AB	AB	AB	AB	AB
AB	AB	AA	AB	AB	AB	AB	AB	AB	AB	AB	AB	AB	AB	AB	AB	AB
AB	AB	AA	AB	AB	AB	AB	AB	AB	AB	AB	AB	AB	AB	AB	AB	AB
AB	AB	AA	AA	AA	AB	AB	AA	AB	AA	AB	AA	AB	AA	AA	AB	AA
AB	AA	AA	AB	AA	AB	AA	AB	AB	AA	AA	AB	AB	AB	AA	AA	AB
AB	AA	AB	AB	-	AB	-	AA	AB	AA	AA	AB	AB	AB	AB	AB	AB
AA	AA	AA	AA	AA	AA	AA	AA	AA	AA	AA	AA	AA	AA	AA	AA	AA
AB	AA	AB	AB	AB	AA	AA	AA	AA	AB	AA	AB	AB	AB	AB	AB	AA
AA	AA	AA	AA	AA	AA	AA	AA	AA	AA	AA	AA	AA	AA	AA	AA	AA
AB	AA	AB	AA	AB	AA	AB	AB	AA	AB	AA	AA	AA	AA	AB	AA	AA
AB	AA	AA	AB	AA	AB	AB	AA	AA	AA	AB	AA	AB	AA	AA	AB	AA
AA	AA	AA	AA	AA	AA	AB	AB	AB	AA	AA	AA	AA	AA	AA	AB	AA
AB	AB	AB	AB	AB	AB	-	AB	AB	AA	-	AB	AB	AA	AB	AB	AA
AB	AB	AA	AA	AA	AB	AA	AB	AB	AB	AA	AB	AB	AB	AA	AA	AB

snp_B5650	snp_B5655	snp_B5658	snp_B5661	snp_B5673	snp_B5678	snp_B5679	snp_B5681	snp_B5690	snp_B5695	snp_B5702	snp_B5707	snp_B5731	snp_B5799	snp_B5821	snp_B5826	snp_B5857	snp_B5873	snp_B5900	snp_B5977
2	2	2	2	2	2	2	2	2	2	2	2	2	2	2	2	2	2	2	2
--	LG1	--	--	LG1	--	LG1	--	--	--	--	LG1	LG1	--	--	--	--	--	--	--

snp_B5985	snp_B5991	snp_B5993	snp_B5994	snp_B6006	snp_B6010	snp_B6019	snp_B6041	snp_B6156	snp_B6157	snp_B6161	snp_B6269	snp_B6354	snp_B6358	snp_B0005	snp_B0016	snp_B0022	snp_B0045	snp_B0047	snp_B0069	snp_B0118
2	2	2	2	2	2	2	2	2	2	2	2	2	2	3	3	3	3	3	3	3
--	--	--	--	LG1	--	--	--	--	--	--	--	--	--	--	LG9	--	LG9	--	--	--

snp_B0144	snp_B0194	snp_B0198	snp_B0250	snp_B0297	snp_B0301	snp_B0304	snp_B0316	snp_B0335	snp_B0345	snp_B0347	snp_B0353	snp_B0355	snp_B0374	snp_B0408	snp_B0416	snp_B0475	snp_B0491	snp_B0518
3	3	3	3	3	3	3	3	3	3	3	3	3	3	3	3	3	3	3
--	--	--	--	--	LG9	--	--	--	--	--	--	--	--	LG9	LG9	--	--	--

snp_B0524	snp_B0539	snp_B0562	snp_B0575	snp_B0580	snp_B0636	snp_B0654	snp_B0677	snp_B0684	snp_B0707	snp_B0735	snp_B0772	snp_B0790	snp_B0796	snp_B0816	snp_B0828	snp_B0832	snp_B0850	snp_B0861	snp_B0880	snp_B0900	snp_B0961	snp_B098[illegible]
3	3	3	3	3	3	3	3	3	3	3	3	3	3	3	3	3	3	3	3	3	3	3
--	--	--	--	--	--	--	--	--	--	--	--	--	LG9	--	--	--	--	--	--	--	--	--
AA	AB	AB	AA	AA	AB	AB	AA	AB	AA	AA	AA	AB	AA	AA	AB	AA	AA	AA	AB	AB	AB	AA
AB	AA	AA	AB	AB	AA	AB	AA	AA	AB	AA	AA	AA	AA	AA	AA	AA	AA	AA	AA	AB	AA	AB
AA	AB	AB	AA	AA	AB	AB	AB	AA	AB	AA	AA	AB	AA	AA	AB	AB	AA	AA	AA	AB	AB	AB
AB	AA	AA	AA	AA	AA	AA	AA	AA	AA	AA	AA	AA	AB	AB	AA	AA	AA	AB	AA	AA	AA	AA
AB	AB	AB	AA	AA	AB	AB	AA	AB	AA	AB	AB	AB	AA	AB	AA	AA	AA	AB	AB	AA	AA	AB
AA	AB	AB	AA	-	AB	AB	AB	-	AA	AA	AB	AB	AA	AA	AB	AB	AB	AB	-	AA	-	AB
AB	AA	AA	AA	-	AA	AA	AA	AA	AA	AA	AA	AA	AA	AA	AA	AA	AA	AA	AA	AA	AA	AA
AB	AB	AB	AB	AB	AB	AB	AB	AB	AB	AB	AB	AB	AB	AB	AB	AB	AB	AB	AB	AB	AB	AB
AA	AB	AB	AA	AA	AB	AB	AA	AA	AB	AA	AB	AB	AB	AA	AB	AA	AA	AB	AA	AA	AB	AB
AA	AA	AA	AA	AA	AB	AB	AA	AA	AB	AA	AA	AA	AA	AA	AB	AA	AA	AA	AA	AA	AB	AA
AA	AA	AB	AB	AA	AB	AA	AA	AA	AB	AB	AA	AA	AB	AA	AB	AA	AA	AB	AB	AA	AA	AB
AA	AB	AB	AA	AB	AB	AB	AB	AA	AA	AA	AB	AB	AA	AA	AB	AA	AA	AA	AA	AA	AB	AB
AB	AA	AA	AA	AA	AA	AA	AB	AA	AA	AA	AA	AA	AA	AA	AA	AB	AA	AA	AA	AA	AA	AA
AB	AA	AA	AB	AB	AA	AA	AA	AB	AB	AB	AA	AA	AB	AB	AA	AB	AB	AA	AA	AA	AA	AA
AA	AB	AA	AA	AA	AB	AB	AB	AA	AB	AB	AB	AB	AA	AA	AB	AB	AA	AA	AA	AB	AB	AB
AB	AA	AB	AB	AB	AB	AB	AB	AA	AB	AB	AB	AB	AB	AB	AB	AB	AB	AB	AB	AB	AB	AB
AA	AB	AB	AB	AB	AB	AB	AA	AA	AB	AA	AB	AB	AB	AA	AB	AA	AB	AA	AB	AA	AB	AB
AA	-	-	AA	-	-	AB	AA	AB	AA	-	-	AA	-	-	AB	-	AA	AB	-	AA	-	AA
AA	AB	AA	AB	AB	AA	AB	AA	AA	AB	AA	AA	AA	AA	AA	AB	AA	AB	AA	AA	AA	AB	AB
AB	AA	AB	AB	AA	AA	AB	AA	AB	AB	AA	AB	AB	-	AA	AB	AB	AB	AA	AB	AB	AA	AB
AA	AB	AB	AB	AB	AB	AA	AA	AB	AB	AB	AA	AB	AB	AB	AB	AA	AB	AB	AB	AA	AB	AA
AB	AB	AB	AA	AB	AB	AB	AB	AB	AB	AB	AB	AB	AA	AB	AB	AB	AB	AB	AB	AA	AB	AB
AB	-	-	AB	-	-	-	AB	-	AB	-	-	AA	-	-	AA	-	AA	AA	AA	AA	-	AB
AA	AA	AA	AA	AA	AA	AA	AA	AA	AA	AA	AA	AA	AA	AA	AA	AB	AB	AA	AA	AB	AA	AA
AA	AB	AA	AB	AB	AA	AA	AA	AB	AB	AB	AA	AA	AB	AB	AA	AA	AA	AA	AB	AA	AA	AA
AA	AB	AA	AB	AA	AA	AA	AA	AA	AB	AA	AB	AA	AB	AB	AA	AA	AB	AB	AA	AA	AA	AB
AA	AB	AB	AA	AB	AB	AA	AA	AA	AB	AA	AA	AA	AA	AB	AB	AA	AA	AB	AA	AA	AB	AA
AA	AB	AA	AA	AA	AA	AA	AB	AB	AA	AA	AA	AA	-	AA	AA	AA	AB	AA	AA	AA	AA	AA
AA	AA	AB	AA	AA	AB	AB	AA	AA	AA	AA	AA	AB	-	AB	AB	AA	AA	AB	AB	AB	AB	AB
AA	AB	-	AA	-	-	AA	AB	AA	AA	AA	AA	AB	-	AA	AA	AA	AA	AA	AA	AA	-	AA
AA	AA	-	AA	AA	AA	AA	AA	AB	AA	AB	AA	AA	AA	AB	AA	AA	AA	AB	AA	AA	AB	AB
AA	AB	AB	AA	AB	AB	AB	AB	AB	AB	AA	AB	AB	AA	AA	AB	AB	AB	AA	AB	AB	AA	AA
AA	-	AA	AA	-	AA	AA	AA	AA	AA	AA	AA	AA	AA	AA	AA	AA	AA	AA	AA	AB	AA	AA
AB	AA	AA	AA	-	AA	AA	AA	AB	AB	AA	AB	AA	-	-	AA	AA	AB	AB	AB	AB	AA	AA
AB	AA	AB	AA	AA	AB	AB	AA	AA	AA	-	AB	AB	AA	AB	AA	-	AA	AB	AA	AA	-	AB
AB	AA	AA	AB	AB	-	AB	AB	AA	AB	AA	AA	AA	AB	-	AB	AB	AB	AA	-	AB	AB	AB
AA	AA	AB	AA	AA	AA	AA	AB	AB	AA	AB	AA	AB	AA	AA	AA	AA	AA	AA	AB	AB	AA	AA
AB	AB	AB	AB	AB	AB	AB	AB	AB	AB	AB	AB	AB	AB	AB	AB	AB	AB	AB	AB	AB	AB	AB
AB	AA	AA	AB	AA	AA	AA	AA	AA	AB	AB	AA	AA	AB	AB	AA	AB	AB	AB	AB	AB	AA	AA
AB	AB	AB	AA	AA	AA	AA	AA	AB	AA	AA	AA	AB	-	-	AA	AA	AA	AA	AA	AA	AA	AA
AA	AA	AB	AB	AB	AB	AB	AB	AB	AA	AB	AA	AB	AB	AA	AA	AB	AA	AB	AB	AB	AB	AA
AA	AB	AB	AA	AB	AB	AB	AA	AB	AB	AA	AB	AB	AA	AA	AB	AA	AA	AA	AA	AA	AA	AA
AB	AA	AA	AB	AA	AA	AA	AB	AA	AA	AA	AA	AA	AB	AB	AA	AB	AA	AA	AA	AA	AB	AA
AA	AB	AA	AA	AA	AA	AA	AB	AA	AA	AA	AA	AA	AA	-	AA	AB	AA	AA	AA	AB	AB	AA
AA	AB	AB	AA	AA	AB	AB	AB	AA	AB	AA	AB	AA	AA	AA	AB	AA	AA	AA	AA	AA	AB	AA
AB	AB	AA	AA	AA	AA	AA	AB	AA	AA	AA	AA	AB	AA	AA	AA	AA	AA	AA	AB	AB	AA	AB
AA	AA	AB	AB	AB	AB	AB	AB	AB	AB	AB	AB	AB	AB	AB	AB	AB	AB	AB	AB	AB	AB	AB
AB	AA	AA	AB	AB	AA	AA	AB	AB	AB	AB	AB	AA	AB	AB	AA	AB	AB	AA	AB	AA	AA	AA
AA	AA	AB	AB	AA	AA	AB	AA	AB	AA	AB	AA	AB	AA	AA	AA	AB	AA	AB	AB	AB	AA	AB
AA	AB	AB	AB	AB	AB	AB	AB	AA	AB	AB	AB	AB	AB	AA	AB	AA	AB	AB	AB	AB	AB	AA
AA	-	AB	AA	AA	AB	AA	AA	AA	AA	AB	AA	AB	AA	AA	AB	AA	AA	AA	AB	AB	AB	AA
AB	AB	AA	AA	-	-	AB	AA	AB	AA	AA	AA	AB	-	AA	AB	AA	AA	AB	AB	AA	AB	AB
AB	AA	AA	AB	AA	AA	AA	AB	AA	AB	AA	AB	AA	AB	AB	AA	AA	AA	AA	AB	AA	AB	AB

snp_B2001	snp_B2016	snp_B2019	snp_B2020	snp_B2021	snp_B2024	snp_B2033	snp_B2060	snp_B2091	snp_B2097	snp_B2099	snp_B2116	snp_B2125	snp_B2197	snp_B2199	snp_B2200	snp_B2202	snp_B2219	snp_B2222	snp_B2226	snp_B2230	snp_B2233	snp_B2245	snp_B2284	snp_B2295	snp_B2305
3	3	3	3	3	3	3	3	3	3	3	3	3	3	3	3	3	3	3	3	3	3	3	3	3	3
--	--	LG9	--	--	LG9	--	--	--	--	LG9	--	--	--	--	--	--	--	--	--	--	--	--	--	--	--

snp_B2318	snp_B2325	snp_B2337	snp_B2347	snp_B2354	snp_B2366	snp_B2373	snp_B2387	snp_B2393	snp_B2411	snp_B2412	snp_B2416	snp_B2418	snp_B2431	snp_B2450	snp_B2453	snp_B2458	snp_B2467	snp_B2486	snp_B2519	snp_B2521	snp_B2544	snp_B2550	snp_B2564	snp_B2571	snp_B2591
3	3	3	3	3	3	3	3	3	3	3	3	3	3	3	3	3	3	3	3	3	3	3	3	3	3
--	--	--	--	--	--	--	--	--	--	LG9	--	--	--	--	--	--	--	--	--	--	--	--	--	--	--

snp_B2600	snp_B2608	snp_B2626	snp_B2707	snp_B2727	snp_B2729	snp_B2736	snp_B2757	snp_B2759	snp_B2810	snp_B2819	snp_B2820	snp_B2830	snp_B2847	snp_B2851	snp_B2858	snp_B2872	snp_B2875	snp_B2893	snp_B2903	snp_B2912	snp_B2917	snp_B2951	snp_B2953	snp_B2989
3	3	3	3	3	3	3	3	3	3	3	3	3	3	3	3	3	3	3	3	3	3	3	3	3
--	--	--	--	--	--	--	--	--	--	--	--	--	--	--	--	--	--	--	--	--	--	--	--	--

snp_B3005	snp_B3052	snp_B3065	snp_B3070	snp_B3081	snp_B3087	snp_B3100	snp_B3133	snp_B3139	snp_B3143	snp_B3168	snp_B3169	snp_B3213	snp_B3218	snp_B3268	snp_B3270	snp_B3301
3	3	3	3	3	3	3	3	3	3	3	3	3	3	3	3	3
--	LG9	--	--	--	LG9	--	--	--	--	--	--	--	--	--	LG9	--
AB	AA	AB	AB	AA	AA	AB	AA	AA	AA	AB	AB	AB	AA	AB	AA	AB
AB	AB	AA	AA	AA	AA	AA	AA	AB	AB	AA	AB	AB	AA	AB	AB	AB
AA	AA	AB	AB	AB	AA	AB	AA	AB	AB	AA	AB	AB	AA	AA	AB	AA
-	AB	AA	AA	AB	AB	AA	AB	AB	AA	AA	AA	AA	AB	AB	AB	AA
AA	AB	AA	AB	AB	AA	AB	AA	AB	AB	AB	AA	AB	AA	AA	AB	AA
-	AA	AB	AB	AB	-	-	-	AA	-	-	AA	AA	-	AB	AA	AB
AA	AA	AA	AA	AA	AA	AA	AB	AA	-	AA	AA	AA	AA	AA	AA	AA
AB	AB	AB	AB	AB	AB	AB	AB	AB	AB	AB	AB	AB	AB	AB	AB	AB
AB	AA	AB	AB	AA	AA	AB	AA	AB	AB	AA	AA	AB	AB	AB	AB	AA
AA	AA	AB	AB	AA	AA	AB	AB	AA	AB	AA	AB	AB	AA	AB	AA	AB
AA	AA	AB	AB	AA	AB	AB	AB	AB	AA	AB	AB	AA	AB	AA	AB	AB
AB	AA	AA	AB	AB	AA	AB	AA	AA	AB	AA	AB	AB	AA	AB	AA	AB
AA	AA	AA	AA	AA	AA	AA	AB	AA	AA	AB	AA	AA	AB	AA	AA	AA
AB	AB	AA	AA	AB	AB	AA	AB	AB	AB	AA	AA	AA	AB	AA	AB	AB
AA	AA	AA	AB	AA	AA	AB	AA	AA	AB	AA	AB	AB	AB	AB	AA	AB
AB	AB	AB	AB	AB	AB	AB	AB	AB	AB	AB	AB	AB	AB	AB	AB	AB
AB	AA	AB	AB	AA	AB	AB	AB	AB	AA	AB	AA	AB	AB	AB	AB	AB
-	AB	-	AA	AA	-	-	-	-	-	AA	-	AA	-	AB	AB	AA
AB	AA	AB	AB	AA	AA	AB	AB	AA	AA	AA	AA	AA	AB	AA	AA	AB
AA	AA	AA	AA	AA	AB	AA	AB	AA	AB	AB	AB	AB	AB	AA	AA	AA
AB	AB	AB	AB	AB	AB	AB	AB	AB	AB	AB	AB	AB	AB	AB	AB	AB
AA	AB	AA	AB	AB	AA	AB	AA	AB	AA	AA	AA	AA	AA	AA	AB	AB
-	AB	AA	AA	AA	-	AA	AB	AA	-	AA	AB	AB	AB	AA	AA	AA
AB	AA	AA	AA	AA	AA	AA	AB	AA	AB	AB	AB	AB	AB	AB	AA	AA
AB	AB	AA	AA	AA	AB	AA	AB	AB	AA	AA	AA	AA	AB	AB	AB	AA
AB	AA	AA	AA	AB	AB	AA	AB	AB	AA	AB	AA	AA	AB	AB	AB	AA
AB	AA	AB	AB	AA	AB	AB	AA	AA	AA	AA	AA	AA	AB	AB	AA	AA
AA	AA	AA	AA	AA	AA	AA	AA	AA	AA	AB	AB	AB	AA	AA	AA	AA
AB	AA	AB	AB	AA	AA	AB	-	AA	AB	AA	AB	AB	AB	AB	AA	AB
-	AA	AA	AA	AA	-	-	-	AA	-	AA	AB	AB	AB	AB	AA	AA
AA	AB	AA	AA	AB	AA	AA	AB	AA	AB	AA	AA	AA	AA	AA	AA	AA
AB	AA	AB	AB	AA	AA	AB	AA	AB	AB	AB	AB	AB	AA	AB	AB	AB
-	AA	AA	AA	AA	-	AA	AA	AA	-	AA	AB	AB	AA	AA	AA	-
-	AB	AA	AA	AB	AA	AA	-	AA	-	AA	AB	AB	AB	AA	AA	AA
-	AB	AA	AB	AB	AA	-	AA	AB	AA	AA	AA	AA	AB	AA	AB	AA
AB	AA	AB	AB	AA	AB	AB	AB	AA	-	AA	AB	AB	AB	AB	AA	AB
AA	AA	AA	AA	AA	AA	AB	AB	AA	AB	AB	AB	AB	AB	AA	AA	AB
AB	AB	AB	AB	AB	-	AB	AB	AB	AB	AB	AB	AB	AB	AB	AB	AA
AA	AB	AB	AA	AA	AB	AA	AB	AB	AA	AB	AA	AA	AB	AA	AB	AA
AA	AA	AA	AA	AA	-	AA	-	-	-	AB	AA	AA	AA	AA	AA	AA
AB	AA	AA	AB	AA	AB	AB	AB	AA	AB	AA	AA	AB	AB	AB	AA	AA
AB	AA	AB	AB	AA	AA	AB	AA	AB	AB	AA	AA	AB	AA	AB	AB	AB
AA	AA	AA	AA	AA	AB	AA	AB	AA	AA	AA	AB	AA	AB	AA	AA	AB
AA	AA	AA	AA	AA	AA	AA	AA	AA	AB	AB	AB	AB	AA	AA	AA	AA
AB	AA	AB	AB	AA	AA	AB	AA	AB	AB	AB	AB	AA	AA	AB	AB	AB

snp_B3959	snp_B3962	snp_B3966	snp_B3979	snp_B3989	snp_B4014	snp_B4051	snp_B4104	snp_B4122	snp_B4129	snp_B4136	snp_B4143	snp_B4150	snp_B4160	snp_B4175	snp_B4179	snp_B4239	snp_B4279	snp_B4293	snp_B4295	snp_B4315	snp_B4321	snp_B4327	snp_B4362
3	3	3	3	3	3	3	3	3	3	3	3	3	3	3	3	3	3	3	3	3	3	3	3
--	--	--	--	--	--	--	--	--	--	--	LG9	--	--	--	--	--	--	--	--	--	--	--	--
AB	AB	AB	AA	AB	AB	AB	AB	AB	AA	AB	AB	AA	AB	AA	AB	AB	AB	AB	AB	AA	AB	AB	AA
AA	AB	AA	AA	AA	AB	AA	AA	AB	AB	AA	AA	AA	AA	AB	AA	AB	AA	AA	AB	AA	AA	AA	AA
AA	AA	AB	AA	AB	AA	AB	AA	AB	AA	AB	AB	AA	AB	AA	AB	AA	AB	AA	AB	AA	AB	AB	AA
AA	AA	AA	AA	AA	AB	AA	AA	AA	AA	AA	AA	-	AA	AA	-	AB	-	AA	AA	AB	-	AA	AA
AB	AA	AA	AB	AA	AA	AB	AB	AB	AA	AB	AA	AB	AA	AA	AA	AA	AA	AA	AA	AA	AA	AA	AB
AB	AB	AB	AB	AA	AB	AB	AA	AA	-	AA	AA	-	AA	-	AB	AB	-	AB	-	AB	-	AA	AA
AA	AA	AA	AA	AA	AA	AA	AA	AA	AA	AA	AA	AA	AA	AA	AA	AA	AA	AA	AA	AA	AA	AA	AA
AB	AB	AB	AB	AB	AB	AB	AB	AB	AB	AB	AA	AB	AB	AB	AB	AB	AB	AB	AB	AB	AB	AB	AB

snp_B4372	snp_B4374	snp_B4376	snp_B4379	snp_B4384	snp_B4391	snp_B4404	snp_B4421	snp_B4458	snp_B4475	snp_B4488	snp_B4491	snp_B4503	snp_B4540	snp_B4553	snp_B4565	snp_B4576	snp_B4598	snp_B4622	snp_B4629	snp_B4642	snp_B4657	snp_B4726	snp_B4755	snp_B4768	snp_B4810	snp_B4811	snp_B4815	snp_B4826
3	3	3	3	3	3	3	3	3	3	3	3	3	3	3	3	3	3	3	3	3	3	3	3	3	3	3	3	3
--	--	LG9	--	--	--	--	--	--	--	--	--	--	--	--	--	--	--	--	--	--	--	--	--	LG9	--	--	--	--
AA	AB	AA	AB	AB	AA	AB	AA	AA	AB	AB	AA	AA	AA	AA	AA	AB	AB	AB	AA	AB	AB	AA	AA	AA	AB	AB	AA	AB
AB	AA	AB	AB	AB	AA	AA	AA	AB	AA	AA	AA	AB	AA	AA	AA	AA	AA	AA	AA	AB	AA	AB	AA	AA	AA	AA	AB	AA
AB	AA	AA	AB	AA	AA	AB	AA	AA	AB	AA	AA	AB	AA	AA	AA	AB	AB	AA	AA	AB	AA	AB	AA	AA	AA	AA	AA	AA
AA	AA	AB	AA	AB	AB	-	AB	AA	AB	AA	AA	-	AA	AB	AA	AA	AB	AB	AB	AA	AB	AA	AB	AB	AA	AA	AA	AA
AB	AA	AB	AB	AA	AB	AB	AA	AA	AA	AA	AB	AB	AB	AA	AB	AB	AB	AB	AB	AA	AB	AA	AB	AB	AB	AB	AA	AB
AB	-	AA	AA	AB	AB	-	AB	AA	AA	-	AB	-	AB	-	-	AB	AB	-	AA	AB	-	AA	AB	AA	AA	AB	AA	AA
AA	AA	AA	AA	AA	AA	AA	AA	AA	AA	AA	AA	AA	AA	AA	AA	AA	AA	AA	AA	AA	AA	AA	AA	AA	AA	AA	AA	AA
AB	AB	AB	AB	AB	AB	AB	AB	AB	AB	AB	AB	AB	AB	AB	AB	AB	AB	AB	AB	AB	AB	AB	AB	AB	AB	AB	AB	AB

snp_B4849	snp_B4854	snp_B4899	snp_B4902	snp_B4920	snp_B4921	snp_B4949	snp_B4955	snp_B4961	snp_B4995	snp_B5002	snp_B5010	snp_B5011	snp_B5020	snp_B5030	snp_B5053	snp_B5058	snp_B5062	snp_B5068	snp_B5071	snp_B5073	snp_B5081	snp_B5108	snp_B5117
3	3	3	3	3	3	3	3	3	3	3	3	3	3	3	3	3	3	3	3	3	3	3	3
LG9	--	--	--	--	--	--	--	--	--	--	--	--	--	--	--	--	LG9	--	--	LG9	--	--	--
AA	AA	AB	AA	AA	AA	AA	AB	AB	AB	AB	AB	AB	AB	AA	AB	AB	AA	AA	AA	AA	AA	AB	AB
AA	AA	AB	AA	AB	AA	AB	AA	AA	AB	AA	AA	AB	AA	AA	AA	AA	AB	AA	AB	AB	AB	AA	AB
AA	AB	AA	AA	AA	AA	AB	AB	AA	AA	AB	AB	AB	AA	AA	AB	AB	AB	AA	AA	AA	AA	AA	AB
AB	AA	AB	AB	AB	-	AA	AA	AA	AA	AA	AA	AA	AA	AA	AA	AB	AB	AA	AA	AB	AA	AA	AA
AB	AA	AA	AA	AA	AB	AB	AA	AA	AB	AA	AA	AB	AA	AB	AB	AA	AB	AB	AB	AB	AA	AB	AA
AA	AA	AB	AA	AA	AA	AA	AA	AB	AA	AA	AA	AA	AB	-	AA	AB	AA	AB	AB	-	AB	AB	AA
AA	AA	AA	AA	AB	AA	AA	AA	AA	AA	AA	AA	AA	AA	AA	AA	AA	AA	AA	AA	AA	AA	AA	AA
AB	AB	AB	AB	AA	AB	AB	AB	AB	AB	AB	AB	AB	AB	AB	AB	AB	AB	AB	AB	AB	AB	AB	AB

snp_B51503	snp_B51523	snp_B51533	snp_B51783	snp_B51983	snp_B52003	snp_B52103	snp_B52163	snp_B52213	snp_B52473	snp_B52483	snp_B52633	snp_B52683	snp_B52923	snp_B53003	snp_B53083	snp_B53393	snp_B53473	snp_B53883	snp_B5400
--	--	--	--	--	--	--	--	--	--	--	--	--	--	--	--	--	--	--	--
AB	AB	AB	AB	AB	AB	AB	AA	AB	AA	AB	AA	AB	AA	AA	AB	AA	AA	AA	AA
AB	AA	AA	AB	AA	AB	AB	AB	AB	AB	AA	AA	AB	AA	AB	AA	AB	AA	AB	AA
AB	AA	AB	AB	AB	AB	AB	AA	AB	AA	AB	AA	AB	AB	AA	AA	AA	AA	AA	AA
AA	AA	AA	AA	AA	AA	AA	AA	AA	AA	AA	AB	AA	AA	AB	AA	AA	AA	AB	AA
AA	AB	AB	AA	AA	AA	AB	AA	AA	AB	AB	AB	AA	AA	AB	AA	AA	AA	AB	AB
-	AB	AB	AA	AA	AA	AA	AA	AA	AA	AB	AA	-	AB	AA	AB	-	AB	AB	AA
AA	AA	AA	AA	AA	AA	AA	AA	AA	AA	AA	AA	AA	AA	AB	AA	AA	AA	AA	AA
AB	AB	AB	AB	AB	AB	AB	AB	AB	AB	AB	AB	AB	AB	AA	AB	AB	AB	AB	AB
AA	AA	AB	AA	AB	AA	AB	AA	AA	AA	AB	AA	AA	AA	AA	AB	AA	AB	AA	AA
AB	AA	AA	AA	AB	AA	AB	AA	AA	AA	AB	AA	AB	AA	AA	AB	AA	AA	AB	AA
AA	AB	AB	AB	AB	AB	AB	AB	AA	AB	AA	AB	AB	AB	AB	AA	AA	AA	AB	AB
AB	AB	AB	AB	AB	AA	AB	AB	AA	AB	AA	AB	AA	AB	AB	AA	AA	AB	AA	AB
AA	AA	AA	AA	AA	AA	AA	AA	AA	AA	AA	AA	AA	AB	AB	AB	AA	AB	AA	AA
AB	AA	AA	AA	AA	AA	AA	AA	AA	AB	AB	AB	AA	AA	AA	AA	AA	AB	AA	AB
AB	AB	AB	AB	AB	AB	AA	AA	AB	AB	AB	AA	AB	AB	AA	AA	AB	AB	AB	AA
AB	AB	AB	AB	AA	AB	AB	AB	AB	AB	AB	AB	AB	AB	AB	AB	AB	AB	AB	AB
AA	AA	AB	AA	AA	AA	AB	AB	AA	AA	AB	AA	AA	AA	AA	AB	AB	AA	AB	AA
AA	AB	AB	AA	AB	AA	AB	AA	AA	-	AB	AB	AA	AA	AA	-	-	AB	AA	AA
AA	AA	AA	AA	AB	AB	AA	AB	AA	AA	AA	AA	AB	AB	AB	AA	AB	AB	AA	AA
AB	AB	AA	AB	AB	AB	AB	AB	AB	AB	AA	AA	AB	AB	AB	AA	AB	AB	AA	AA
AB	AB	AB	AA	AA	AA	AB	AB	AA	AB	AB	AB	AA	AB	AA	AB	AB	AB	AB	AB
AA	AB	AB	AA	AB	AA	AA	AA	AA	AA	AB	AB	AA	AB	AA	AB	AA	AB	AB	AB
AA	AB	AA	-	AA	AB	-	AA	AB	AA	AA	AB	AB	AB	AA	AB	AA	AA	AA	-
AB	AA	AA	AB	AB	AB	AB	AA	AB	AB	AA	AA	AB	AB	AA	AB	AA	AB	AB	AA
AA	AA	AA	AA	AA	AA	AA	AB	AA	AA	AA	AB	AA	AA	AA	AA	AB	AA	AB	AB
AA	AA	AB	AA	AB	AA	AA	AA	AA	AA	AA	AA	AA	AA	AB	AB	AA	AB	AB	AB
AA	AB	AA	AA	AB	AB	AA	AA	AA	AA	AA	AB	AB	AA	AB	AA	AA	AB	AB	AB
AA	AA	AA	AB	AB	AB	AB	AA	AB	AA	AA	AB	AB	AB	AA	AB	AA	AB	AA	AA
AB	AB	AB	AB	AB	AB	AB	AA	AB	AA	AA	AB	AB	AA	AA	AB	AA	AA	AB	AB
AB	AB	AA	AB	AA	AB	AB	AA	AB	AA	AA	AA	-	AB	AB	AA	-	AB	AB	AA
AA	AB	AA	AA	AA	AA	AA	AA	AA	AB	AA	AB	AA	AA	AB	AB	AA	AA	AA	AB
AB	AA	AB	AB	AB	AB	AB	AA	AB	AB	AB	AA	AB	AB	AB	AB	AA	AB	AA	AA
AB	AA	AA	AB	AB	AB	AB	AA	AB	AB	AA	AA	AB	AA	AA	AA	-	AA	AA	AA
AB	AA	AA	AB	AA	AB	AB	AA	AB	AA	AA	AA	AB	AA	AB	AB	AA	AA	AA	AA
AA	AB	AB	AA	AA	AA	AA	AA	AA	AA	AB	AB	AA	AA	AB	AA	AA	AA	AA	AB
AB	AA	AA	AB	AB	AA	AA	AB	AB	AB	AA	AA	AB	AB	AB	AB	AB	AB	AB	AA
AB	AB	AA	AB	AB	AB	AB	AA	AB	AB	AA	AB	AB	AB	AA	AB	AA	AB	AA	AA
AB	AB	AB	AB	AA	AB	AB	AB	AB	AB	AB	AB	AB	AA	AB	AA	AA	AB	AB	AB
AA	AA	AA	AA	AA	AA	AA	AB	AA	AB	AB	AB	AB	AA	AB	AB	AB	AB	AB	AA
AA	AB	AA	-	AA	AA	AB	AA	AA	AA	AA	AB	AA	AA	AA	AB	AA	AA	AA	AA
AB	AB	AA	AB	AB	AB	AB	AB	AB	AB	AB	AB	AB	AB	AB	AB	AB	AA	AA	AA
AA	AA	AB	AA	AA	AA	AA	AB	AA	AB	AB	AA	AA	AA	AB	AA	AB	AA	AA	AA
AA	AA	AA	AA	AA	AA	AA	AB	AA	AA	AA	AA	AA	AA	AB	AA	AB	AA	AB	AA
AB	AA	AA	AB	AA	AB	AB	AB	AB	AB	AA	AA	AB	AB	AA	AB	AA	AA	AA	AA
AA	AB	AB	AB	AB	AB	AB	AB	AB	AB	AA	AB	AB	AB	AB	AA	AB	AA	AA	AA
AB	AA	AA	AA	AA	AA	AB	AA	AA	AB	AA	AA	AA	AA	AB	AA	AB	AA	AB	AA
AB	AB	AB	AB	AB	AB	AB	AB	AB	AB	AB	AB	AB	AB	AB	AB	AB	AB	AB	AB
AA	AA	AA	AA	AB	AA	AA	AB	AA	AA	AA	AB	AA	AA	AB	AB	AB	AB	AA	AB
AB	AB	AA	AB	AB	AB	AB	AA	AB	AB	AA	AB	AB	AB	AA	AA	AA	AB	AA	AA
AB	AB	AA	AB	AB	AB	AB	AB	AB	AB	AB	AB	AB	AB	AB	AA	AA	AB	AB	AA
AB	AB	AB	AB	AB	AB	AB	AB	AB	AB	AA	AB	AA	AB	AA	AA	AA	AB	AB	AB
-	AA	AB	AA	AB	AB	AB	AB	AA	AB	AA	AB	AA	AA	AB	AA	AB	AA	AA	AA

snp_B0531	snp_B0563	snp_B0567	snp_B0579	snp_B0591	snp_B0607	snp_B0644	snp_B0651	snp_B0701	snp_B0759	snp_B0767	snp_B0769	snp_B0797	snp_B0799	snp_B0820	snp_B0835	snp_B0846	snp_B0852	snp_B0853	snp_B0870	snp_B0879	snp_B0885	snp_B0894	snp_B0924
4	4	4	4	4	4	4	4	4	4	4	4	4	4	4	4	4	4	4	4	4	4	4	4
--	--	--	--	--	--	--	--	--	LG6	--	--	--	--	--	LG6	--	--	--	--	--	--	--	--
AA	AA	AA	AB	AA	AB	AB	AB	AA	AA	AA	AB	AA	AA	AB	AB	AB	AB	AA	AA	AB	AA	AB	AA
AB	AB	AA	AB	-	AA	AA	AA	AA	AB	AB	AB	AA	AA	AB	AB	AB	AB	AA	AA	AB	AA	AB	AA
AA	AA	AB	AB	AB	AB	AB	AB	AB	AB	AA	AA	AA	AA	AB	AB	AB	AB	AA	AA	AB	AB	AB	AA
AB	AB	AA	AB	AB	AA	AB	AA	AA	AB	AA	AB	AA	AB	AB	AB	AB	AB	AB	AB	AB	AA	AB	AB
AA	AA	AB	AA	AA	AB	AA	AA	AA	AA	AA	AA	AA	AA	AA	AB	AB	AB	AA	AA	AA	AA	AA	AA
-	AA	AB	AA	AA	AB	AA	-	AB	AA	AA	AA	-	AA	AA	-	AB	AA	-	AA	AA	AB	AA	AB
AB	AB	AB	AA	AB	AA	AA	AA	AA	AB	-	AA	AB	AB	AA	AA	AA	AA	AB	AB	AA	AB	-	AB
AA	AB	AA	AB	AB	AA	AB	AB	AA	AA	AB	AB	AA	AB	AB	AB	AB	AB	AA	AA	AB	AA	AB	AA
AA	AB	AB	AB	AA	AB	AB	AB	AA	AA	AA	AA	AA	AA	AB	AA	AB	AB	AA	AB	AB	AA	AB	AB
AB	AB	AB	AA	AB	AA	AA	AA	AB	AA	AB	AB	AA	AB	AA	AA	AA	AA	AB	AB	AA	AB	-	AB
AB	AB	AB	AA	AB	AA	AA	AA	AA	AB	AA	AA	AB	AB	AB	AB	AB	AA	AB	AB	AB	AA	AA	AB
AA	AB	AB	AB	AA	AA	AB	AB	AB	AA	AA	AA	AB	AB	AB	AB	AB	AA	AB	AA	AB	AB	AA	AB
AB	AA	AB	AA	AA	AB	AA	AB	AB	AB	AB	AB	AB	AA	AA	AB	AA	AA	AB	AB	AA	AB	AB	AB
AB	AB	AB	AA	AA	AB	AA	AB	AA	AB	AB	AA	AA	AB	AB	AB	AA	AA	AB	AA	AB	AA	AB	AB
AB	AB	AA	AA	AA	AB	AA	AA	AA	AA	AA	AB	AB	AB	AB	AB	AA	AA	AB	AA	AB	AA	AB	AA
AB	AB	AB	AB	AB	AA	AB	AA	AA	AB	AB	AB	AB	AB	AB	AA	AB	AB	AB	AB	AB	AA	AB	AB
AA	AA	AA	AA	AB	AA	AA	AA	AA	AB	AB	AA	AB	AB	AB	AA	AA	AA	AB	AB	AA	AA	AA	AA
-	AA	AB	-	AB	AA	AB	-	AA	AA	AB	AA	-	AA	AB	AA	-	AB	AA	-	AB	AB	AB	AA
AA	AB	AA	AB	AB	AA	AA	AA	AA	AB	AA	AB	AA	AA	AB	AB	AA	AA	AA	AB	AB	AA	AB	AA
AA	AB	AA	-	AB	AA	AB	AB	AA	AB	AA	AB	AA	AB	AB	AB	AB	AB	AA	AB	AB	AA	-	AA
AB	AB	AB	AB	AB	AB	AB	AB	AB	AB	AA	AB	AB	AB	AB	AA	AA	AB	AB	AB	AB	AB	AB	AA
AB	AA	AA	AA	AB	AA	AA	AA	AA	AB	AB	AA	AB	AB	AA	AA	AA	AA	AA	AB	AA	AA	AB	AA
-	AA	AA	-	-	AB	AB	-	AA	AA	AA	AB	-	AA	AB	AA	AA	AB	AA	AA	AB	AA	AB	AA
AA	AA	AA	AA	AB	AA	AA	AB	AA	AB	AA	AA	AA	AB	AB	AB	AA	AB	AA	AA	AB	AA	AA	AB
AB	AB	AA	AB	AB	AA	AB	AA	AA	AB	AB	AB	AA	AB	AB	AB	AB	AA	AA	AB	AB	AA	AB	AA
AB	AA	AB	AA	AA	AA	AB	AA	AA	AA	AA	AA	AA	AA	AA	AB	AB	AA	AA	AB	AB	AA	AA	AA
AA	AB	AA	AB	AB	AA	AB	AB	AA	AB	AA	AB	AB	AB	AB	AB	AB	AB	AA	AB	AB	AA	AB	AA
AA	AB	AB	AA	AA	AB	AA	AA	AB	AB	AB	AB	AA	AB	AA	AA	AA	AA	AB	AB	AA	AB	AA	AB
AA	AB	AA	AB	-	AB	AB	AB	AB	AB	AB	AB	AA	AB	AB	AB	AB	AB	AA	AB	AB	AA	AA	AB
AB	AB	-	-	-	AA	AB	-	AA	AA	AA	AA	-	AA	AB	AB	-	AB	AA	AA	AB	AB	-	AB
AB	AA	AB	AB	AA	AB	AA	AB	AB	AA	AB	AA	AA	AA	AB	AB	AB	AA	AB	AA	AA	AB	AB	AB
AB	AB	AB	AB	AB	AB	AB	AB	AB	AB	AB	AB	AB	AB	AB	AB	AB	AB	AB	AB	AB	AB	AB	AB
AA	AA	AB	AA	-	AB	AA	AA	AB	AA	AA	AA	-	AB	AA	AB	AB	AA	AB	-	AA	AB	AA	AB
-	AB	-	AA	AA	AA	AA	-	AA	AB	AB	AA	AA	AB	AA	AA	-	AA	-	AB	AA	AA	-	AB
-	AA	AB	-	AA	AA	AA	-	AA	AA	AA	AA	AA	AA	AA	-	AB	AA	AB	AA	AA	AA	AA	AB
AB	AB	AB	AB	AB	AB	AB	AB	AB	AB	AB	AB	AB	AB	AB	AB	AB	AB	-	AB	AB	AB	AB	AB
AA	AB	AA	AB	AB	AA	AB	AB	AA	AB	AB	AB	AB	AB	AB	AB	AB	AA	AA	AB	AB	AA	AA	AA
AB	AB	AB	AA	AB	AB	AB	AA	AB	AB	AB	AA	AB	AB	AA	AA	AA	AB	AB	AB	AA	AB	AB	AB
AA	AA	AA	AA	AB	AB	AA	AA	AB	AB	AB	AB	AA	AB	AA	AA	AA	AB	AB	AB	AA	AB	AB	AB
AA	AA	-	AA	AA	AB	AB	-	AA	AB	AA	AA	-	AB	AB	AA	AA	AB	AA	AB	AB	AA	AB	AA
AA	AB	AA	AB	AA	AB	AB	AB	AB	AA	AA	AA	AA	AA	AB	AA	AB	AA	AA	AB	AB	AB	AB	AA
AB	AB	AB	AA	AB	AA	AA	AB	AA	AB	AA	AB	AA	AA	AA	AB	AA	AA	AB	AB	AA	AB	AB	AA
AB	AB	AA	AA	-	AA	AA	AB	AA	AB	AB	AB	AB	AB	AA	AB	AA	AA	AB	AB	AA	AB	AB	AA
AB	AB	AB	AB	AB	AB	AB	AB	AB	AA	AA	AA	AB	AA	AB	AA	-	AB	-	AA	AB	AB	AB	AA
AA	AA	AA	AA	AB	AA	AA	AA	AB	AB	AA	AA	AB	AB	AA	AA	AA	AB	AB	AA	AB	AA	AB	AB
AB	AA	AB	AA	AA	AB	AA	AA	AB	AA	AA	AA	AA	AA	AA	AB	AB	AB	AB	AA	AA	AB	AA	AB
AA	AB	AA	AB	AA	AB	AB	AB	AB	AA	AA	AA	AB	AA	AB	AB	AB	AB	AB	AA	AA	AB	AA	AB
AA	AA	AB	AA	AA	AB	AB	AB	AB	AA	AA	AA	AB	AA	AB	AA	AB	AB	AB	AA	AA	AB	AA	AB
AB	AA	AB	AA	AA	AB	AA	AB	AB	AA	AA	AA	AA	AA	AA	AB	AB	AA	AB	AA	AA	AB	AB	AB
AB	AA	AB	AA	AB	AB	AA	AB	AB	AA	AB	AB	AB	AA	AA	AB	AB	AA	AB	AA	AA	AB	AB	AB
AA	AA	AB	AA	AA	AB	AA	AA	AB	AB	AA	AB	AB	AB	AB	AA	AB	AB	AB	AB	AA	AB	AA	AB
AB	AB	AB	AB	-	AA	AA	AB	AA	AB	AA	AA	-	AA	AB	AB	AA	AA	AB	-	AB	AA	AB	AA
AB	AA	AB	AB	-	AB	AA	AB	AB	AB	AB	AA	AB	AA	AB	AB	AA	AA	AB	AA	AA	AB	AB	AB
AB	AA	AB	AB	-	AB	AA	AA	AB	AB	AA	AB	AA	AA	AB	AB	AB	AA	AB	AA	AA	AB	AB	AB

snp_B1647	snp_B1667	snp_B1674	snp_B1689	snp_B1705	snp_B1743	snp_B1747	snp_B1762	snp_B1767	snp_B1814	snp_B1848	snp_B1849	snp_B1854	snp_B1882	snp_B1923	snp_B1938	snp_B2026	snp_B2042	snp_B2052	snp_B2055	snp_B2076	snp_B2108
4	4	4	4	4	4	4	4	4	4	4	4	4	4	4	4	4	4	4	4	4	4
--	--	--	--	LG6	--	--	--	--	--	--	--	--	--	--	--	--	--	--	--	LG6	--
AB	AA	AB	AB	AA	AA	AB	AA	AB	AA	AB	AA	AB	AA	AA	AA	AB	AA	AB	AA	AA	AA
AA	AB	AB	AB	AB	AB	AA	AB	AB	AA	AB	AB	AB	AB	AB	AB	AA	AB	AA	AA	AB	AB
AB	AA	AB	AA	AA	AB	AB	AB	AA	AA	AB	AB	AB	AA	AB	AB	AA	AB	AB	AB	AA	AA
AB	AB	AB	AB	AB	AB	AA	AB	AA	AA	AB	AB	AB	-	AB	AB	AA	-	AA	-	AA	AB
AA	AA	AA	AA	AA	AA	AB	AA	AA	AA	AA	AA	AA	AA	AA	AA	AA	AA	AA	AA	AA	AA
-	AB	AA	AA	AA	-	AB	AA	AA	-	AA	-	AA	-	AA	AA	AB	-	AB	-	AA	AA
AA	AB	AA	-	AB	AA	AA	AB	AB	AB	AA	AA	AA	AB	AA	AB	AA	AB	AA	AA	AA	AB
AB	AA	AB	AB	AA	AB	AA	AB	AA	AA	AB	AA	AB	AA	AA	AA	AA	AB	AA	AB	AA	AA
AB	AA	AB	AA	AA	AB	AA	AB	AA	AA	AB	AB	AB	AA	AA	AA	AB	AB	AB	AB	AA	AA
AB	AA	AB	AB	AB	AB	AA	AB	AA	AA	AB	AA	AA	AB	AA	AB	AA	AB	AB	AA	AB	AB
AA	AB	AA	AA	AB	AA	AA	AB	AB	AB	AA	AA	AA	AB	AB	AA	AA	AB	AB	AA	AB	AB
AB	AB	AB	AA	AA	AA	AA	AA	AA	AB	AB	AB	AB	AA	AB	AB	AA	AB	AB	AB	AB	AB
AA	AB	AA	AA	AB	AA	AB	AB	AB	AB	AA	AB	AB	AB	AB	AA	AB	AA	AB	AA	AA	AB
AA	AB	AA	AB	AB	AB	AB	AB	AB	AB	AA	AB	AA	AB	AA	AB	AB	AA	AB	AB	AA	AB
AA	AB	AA	AB	AA	AB	AA	AB	AA	AB	AA	AB	AA	AB	AB	AB	AB	AA	AB	AB	AB	AA
AB	AB	AB	AB	AB	AB	AA	AB	AB	AB	AB	AA	AB	AB	AB	AB	AB	AB	AA	AA	AA	AB
AA	AA	AA	AB	AB	AA	AA	AB	AB	AB	AA	AA	AA	AB	AA	AB	AA	AB	AA	AA	AA	AB
AA	AA	AA	-	AA	-	AB	AA	AB	AB	-	AA	AB	-	AB	AA	AB	-	AB	-	-	AA
AA	AA	AB	AB	AB	AB	AA	AB	AB	AA	AB	AB	AB	AB	AB	AB	AA	AB	AA	AB	AA	AB
AB	AA	AB	AB	AB	AB	AB	AB	AB	AB	AB	AA	AB	AB	AB	AB	AB	AB	AB	AB	AB	AB
AB	AB	AB	AB	AB	AB	AA	AB	AB	AB	AB	AA	AB	AB	AB	AB	AB	AB	AB	AB	AB	AB
AA	AA	AA	AA	AB	AA	AA	AB	AB	AA	AA	AB	AA	AB	AB	AA	AA	AA	AA	AA	AB	AB
-	-	AB	-	AA	AB	-	-	AA	-	-	-	AB	AA	AA	AA	AA	-	AA	-	AA	AA
AB	AA	AB	AA	AB	AA	AA	AB	AA	AB	AA	AA	AB	AA	AB	AA	AA	AA	AB	AB	AB	AB
AB	AA	AB	AB	AB	AB	AA	AB	AB	AA	AB	AB	AB	AB	AB	AB	AA	AB	AA	AB	AB	AB
AB	AB	AA	AA	AA	AB	AA	AA	AB	AB	AA	AA	AA	AA	AB	AA	AA	AB	AB	AA	AB	AB
AB	AA	AB	AB	AB	AB	AA	AB	AB	AB	AB	AA	AB	AB	AB	AB	AA	AB	AB	AB	AB	AB
AA	AB	AB	AB	AB	AB	AB	AB	AA	AA	AA	AA	AA	AB	AA	AB	AA	AA	AB	AB	AB	AB
-	AA	AB	AA	AA	AA	AB	AA	AA	AA	-	-	AB	AA	AB	AA	AB	AA	AB	AB	AA	AB
-	AA	AB	-	AA	-	AA	-	AA	AB	AB	-	AB	AA	AB	AB	AA	-	AA	-	AA	AB
AA	AB	AB	AA	AA	AB	AB	AA	AA	AA	AB	AA	AB	AA	AB	AA	AB	AA	AB	AB	AA	AA
AB	AB	AB	AB	AB	AB	AB	AB	AB	AB	AB	AB	AB	AB	AB	AB	AB	AB	AB	AB	AA	AB
AA	AB	AA	AA	AA	AA	AB	AA	AA	AA	-	AA	AA	AA	AA	AA	AB	-	AB	AA	AA	AA
-	AB	AA	AA	AA	AA	AA	AA	AA	AA	AA	AB	AA	AB	AB	AA	AB	AA	AA	-	-	AB
AA	AB	AA	-	AA	-	AA	AA	AA	AA	AA	AA	AA	-	AA	AA	AB	-	AB	-	AA	AA
AB	AB	AB	AB	AB	AB	AB	AB	AB	AB	-	AB	AB	AB	AB	AB	AB	AB	AB	AB	AB	AB
AB	AA	AB	AB	AB	AB	AB	AB	AB	AB	AB	AA	AA	AA	AB	AB	AA	AB	AB	AB	AB	AB
AB	AB	AA	AB	AB	AA	AB	AB	AB	AB	AA	AA	AA	AB	AA	AB	AA	AB	AB	AB	AA	AB
AB	AB	AA	AB	AB	AB	AB	AB	AB	AB	AA	AA	AA	AB	AB	AA	AB	AA	AB	AA	AA	AA
-	AA	AA	-	AB	AB	AB	AB	AB	AB	AA	AA	AA	AB	AB	AB	AA	AA	AA	-	AA	AA
AB	AA	AB	AB	AA	AA	AB	AA	AA	AA	AB	AB	AB	AA	AB	AA	AB	AB	AA	AB	AA	AB
AA	AB	AA	AB	AB	AA	AA	AB	AA	AA	AA	AA	AA	AB	AB	AB	AA	AB	AB	AB	AB	AB
AA	AB	AA	AB	AB	AB	AA	AB	AB	AA	AA	AB	AB	AB	AA	AB	AA	AB	AB	AB	AB	AB
AB	AB	AB	-	AA	AB	AB	AA	AB	AB	-	AA	AB	AB	AA	AB	AB	-	AB	AB	AB	AB
AA	AB	AA	AB	AB	AA	AB	AB	AB	AA	AA	AB	AB	AA	AA	AA	AB	AA	AB	AA	AB	AB
AA	AB	AA	AA	AA	AA	AB	AA	AA	AB	AA	AA	AB	AA	AA	AA	AB	AA	AA	AA	AA	AA
AA	AB	AB	AA	AA	AA	AB	AA	AA	AB	AB	AB	AB	AA	AB	AA	AB	AB	AB	AA	AA	AB
AB	AB	AB	AA	AA	AA	AB	AA	AA	AB	AA	AA	AB	AA	AB	AA	AB	AA	AB	AB	AA	AB
AA	AB	AA	AA	AA	AA	AB	AA	AA	AB	AB	AB	AB	AA	AB	AA	AB	AB	AA	AB	AA	AA
AB	AB	AB	AB	AB	AB	AB	AB	AB	AB	AB	AB	AB	AB	AB	AB	AB	AB	AB	AB	AB	AB
AA	AB	AA	AA	AA	AA	AB	AA	AB	AB	AA	AA	AB	AB	AB	AB	AA	AB	AB	AA	AA	AB
AA	AB	AB	-	AA	AA	AA	AA	AB	AA	AB	AB	AA	AB	AB	AA	AA	AA	AA	-	AB	AA
AA	AB	AB	AA	AA	AB	AB	AA	AB	AA	AB	AB	AA	AB	AA	AB	AA	AA	AB	AA	AA	AB

snp_B3109	snp_B3128	snp_B3141	snp_B3146	snp_B3154	snp_B3167	snp_B3175	snp_B3207	snp_B3216	snp_B3225	snp_B3227	snp_B3253	snp_B3259	snp_B3265	snp_B3304	snp_B3333	snp_B3341	snp_B3348	snp_B3365	snp_B3370	snp_B3387
4	4	4	4	4	4	4	4	4	4	4	4	4	4	4	4	4	4	4	4	4
--	--	--	--	--	--	--	--	--	--	--	--	--	--	--	LG6	--	--	--	--	--
AA	AA	AA	AB	AB	AB	AA	AB	AB	AB	AA	AB	AB	AA	AA	AA	AB	AA	AB	AB	AA
AA	AB	AB	AA	AA	AA	AB	AB	AA	AB	AB	AB	AB	AB	AB	AB	AA	AB	AA	AB	AB
AB	AB	AA	AA	AB	AB	AA	AA	AB	AB	AB	AA	AB	AB	AB	AA	AB	AB	AB	AB	AB
AA	AB	AB	AA	AA	AB	AB	-	AB	AB	AB	AB	AB	AB	AA	AB	AA	AA	AA	AA	AA
AB	AA	AB	AA	AB	AB	AA	AA	AA	AA	AA	AB	AA	AA	AA	AA	AB	AA	AB	AA	AB
AB	AA	AB	AA	-	-	AA	-	AA	-	AB	-	AA	AA	-	AA	AB	AA	AB	AA	-
AA	-	AB	AB	AA	AA	AA	-	AA	AA	AB	AB	AA	AB	AB	AB	AB	AA	AB	-	AB
AA	AB	AA	AB	AB	AB	AA	AB	AB	AA	AA	AA	AB	AB	AA	AA	AB	AB	AA	AB	AA
AB	AB	AA	AA	AB	AB	AA	AA	AB	AB	AB	AB	AB	AB	AB	AA	AB	AB	AA	AB	AB
AB	AA	AB	AA	AA	AA	AB	AB	AB	AA	AB	AB	AB	AA	AB	AB	AA	AA	AA	AA	AB
AA	AA	AB	AB	AA	AA	AB	AB	AA	AA	AA	AB	AB	AB	AB	AB	AB	AB	AB	AA	AB
AB	AA	AA	AB	AB	AB	AA	AA	AB	AA	AB	AA	AB	AA	AB	AA	AB	AB	AB	AB	AB
AB	AB	AB	AB	AB	AB	AB	AA	AA	AB	AB	AB	AB	AA	AB	AB	AB	AB	AB	AB	AB
AA	AA	AB	AA	AA	AA	AB	AB	AA	AA	AB	AB	AA	AA	AB	AB	AA	AB	AA	AA	AA
AA	AA	AB	AA	AA	AA	AB	AA	AA	AA	AA	AB	AB	AB	AA	AA	AB	AA	AA	AA	AA
AA	AB	AB	AA	AA	AA	AB	AB	AB	AA	AB	AB	AA	AB	AB	AB	AA	AA	AB	AA	AB
AA	AA	AA	AB	AA	AA	AB	AA	AA	AA	AA	AA	AA	AB	AA	AB	AA	AA	AA	AA	AA
AB	-	AA	AB	-	AB	AA	-	AA	-	AB	-	AB	-	AA	-	AA	AB	AB	AB	AA
AA	AB	AA	AA	AB	AB	AA	AB	AA	AA	AA	AA	AB	AB	AA	AB	AA	AB	AA	AB	AA
AA	AB	AA	AA	AA	AB	AB	AB	AB	AA	AA	AA	AB	AB	AA	AB	AB	AA	AA	AA	AA
AB	AB	AB	AB	AB	AA	AB	AB	AB	AB	AB	AB	AB	AB	AB	AB	AB	AB	AA	AB	AB
AA	AA	AA	AB	AA	AA	AA	AB	AA	AB	AA	AA	AA	AB	AA	AB	AB	AA	AA	AA	AA
AB	AB	AA	AB	AB	-	AA	-	AB	AB	AA	-	AA	AA	AA	-	AA	AB	AA	-	-
AA	AA	AA	AA	AA	AB	AB	AB	AB	AA	AA	AA	AB	AB	AA	AB	AB	AB	AA	AB	AA
AA	AB	AA	AA	AA	AB	AA	AB	AB	AB	AA	AA	AB	AB	AA	AB	AB	AB	AA	AB	AA
AA	AB	AA	AA	AA	AB	AA	AA	AB	AA	AB	AB	AA	AB	AB	AA	AA	AB	AB	AA	AB
AA	AB	AA	AB	AA	AB	AA	AB	AA	AB	AA	AA	AB	AB	AA	AB	AA	AB	AA	AB	AA
AB	AB	AB	AA	AB	AA	AA	AB	AA	AB	AB	AB	AA	AB	AB	AB	AB	AA	AB	AA	AB
AB	AB	AA	AB	AB	AB	AA	AB	AB	AB	AB	AA	AB	AA	-	AA	AA	AB	AB	AB	AB
AA	-	AA	AB	AA	-	AA	-	AA	-	AB	-	AB	AA	-	AA	AB	AB	AB	AB	AB
AB	AB	AB	AA	AA	AA	AA	AB	AA	AB	AB	AB	AB	AA	AB	AA	AB	AB	AB	AB	AB
AB	AB	AB	AB	AB	AB	AA	AB	AB	AB	AB	AB	AB	AB	AB	AB	AB	AB	AB	AB	AB
AB	AA	AB	AA	AB	-	AA	-	AA	AA	AA	AB	AA	AA	AB	AA	AA	AA	AB	AA	AB
AA	AA	AB	AA	AA	AA	AB	-	AA	AA	AB	-	AA	AA	-	AA	AB	AA	AA	-	AA
AA	AA	AB	AA	AA	AB	AB	-	AA	AA	AB	AB	AA	AA	AB	-	AA	AA	AB	AA	AB
AB	AB	AB	AB	AB	AB	AB	AB	AB	AB	AB	AB	AB	AB	-	AB	AB	AB	AB	AB	AB
AA	AB	AA	AB	AB	AA	AB	AB	AB	AA	AA	AA	AA	AB	AA	AB	AA	AB	AA	AA	AA
AB	AA	AB	AB	AB	AA	AB	AA	AB	AB	AB	-	AA	AB	AB	AB	AB	AA	AB	AA	AB
AB	AB	AB	AA	AB	AA	AB	AB	AB	AA	AA	AB	AA	AB	AA	AB	AB	AA	AB	AA	AB
AB	AA	AB	AA	AB	AA	AA	-	AB	AA	AA	AB	AA	AB	-	AB	AB	AA	AB	-	AB
AB	AB	AA	AB	AB	AB	AB	AA	AB	AB	AA	AA	AB	AA	AA	AA	AB	AB	AA	AB	AA
AA	AA	AB	AA	AA	AA	AB	AA	AA	AA	AB	AB	AB	AA	AB	AB	AB	AB	AA	AB	AB
AB	AA	AB	AB	AA	AB	AA	AA	AA	AB	AB	AB	AB	AB	AA	AB	AB	AB	AA	AB	AA
AA	AB	AA	AB	AA	AA	AA	AA	AB	AB	AB	AA	AB	AA	AA	AB	AB	AB	AB	-	AB
AB	AA	AA	AB	AA	AB	AA	AB	AA	AA	AA	AB	AA	AB	AA	AB	AB	AB	AB	AA	AB
AB	AA	AB	AA	AB	AB	AB	AA	AA	AB	AB	AB	AA	AA	AB	AA	AB	AA	AB	AA	AB
AB	AA	AB	AA	AB	AB	AB	AA	AA	AA	AB	AB	AB	AA	AB	AA	AA	AB	AA	AB	AB
AB	AB	AB	AA	AB	AB	AA	AA	AB	AA	AB	AB	AB	AA	AB	AA	AB	AB	AB	AB	AB
AB	AA	AA	AA	AB	AA	AB	AA	AB	AA	AA	AA	AB	AA	AA	AB	AB	AB	AB	AB	AB
AB	AB	AB	AA	-	-	AB	-	AA	AA	AB	-	AA	AA	AB	AA	AA	AB	AB	AB	AB
AB	AB	AB	AB	AB	AA	AB	AB	AA	AB	AB	AB	AA	AB	AB	AA	AB	AA	AB	AA	AB
AB	AB	AB	AB	AA	AB	AB	AB	AA	AB	AB	AB	AB	AA	AB	AA	AB	AB	AB	AA	AB
AA	-	AA	AA	AA	AA	AB	AB	AB	AA	AA	AB	AA	AA	AA	AA	AB	AB	AB	AB	AA
AB	AB	AB	AB	AB	AA	AA	AB	AB	AB	AB	AB	AB	AB	AB	AB	AB	AB	AB	AB	AB
AA	AA	AB	AB	AB	AA	AA	AB	AB	AA	AB	AA	AB	AB	AA	AB	AB	AB	AB	AB	AB
AB	AA	AB	AA	AB	AB	AA	AA	AA	AB	AB	AB	AB	AA	AB	AA	AB	AB	AB	AB	AB
AA	AB	AB	AB	AB	AB	AA	AB	AA	AB	AB	AA	AB	AA	AB	AB	AB	AB	AB	AA	AB
AA	AA	AB	AA	AA	AB	AA	AA	AA	AB	AA	AB	AA	AB	AA	AB	AA	AB	AA	AB	AB
AB	AB	AB	AB	AB	AB	AA	AB	AB	AB	AA	AA	AB	AB	AB	AA	AB	AB	AA	AB	AA
AB	AB	AB	AB	AB	AB	AA	AA	AB	AB	AB	AA	AB	AA	AB	AA	AB	AB	AB	AB	AB
AB	AA	AB	AB	AB	AB	AB	AB	AB	AB	AB	AB	AB	AA	AB	AB	AB	AA	AB	AB	AB
AB	-	AB	AB	AB	-	AB	AA	AA	AB	AB	-	AA	AA	AB	AA	AB	AA	AB	AA	AB
AB	AB	AB	AA	AB	AA	AB	AB	AB	AB	AB	AB	AA	AB	AB	AB	AB	AA	AB	AA	AB
AA	AA	AB	AA	AB	AB	AB	AA	AB	AB	AB	AB	AA	AB	AB	AB	AB	AA	AB	AA	AB
AB	AA	AA	AB	AB	AA	AB	AB	AB	AB	AA	AA	AB	AA	AA	AA	AA	AB	AA	AB	AA
AB	-	AB	AB	AA	AB	AB	AA	AA	AB	AB	-	AB	AA	AA	AA	AB	AB	AB	AB	AB
AB	AA	AB	AB	AA	AB	AB	AA	AA	AA	AB	AB	AA	AA	AB	AA	AB	AA	AB	AB	AB
AB	AA	AB	AB	AA	AB	AB	AA	AA	AA	AB	AB	AA	AA	AB	AA	AB	AA	AB	AA	AB
AB	AA	AB	AB	AB	AB	AA	AA	AB	AB	AB	AB	AA	AA	AB	AA	AB	AA	AB	AA	AB

snp_B4158	snp_B4163	snp_B4174	snp_B4177	snp_B4190	snp_B4209	snp_B4211	snp_B4216	snp_B4217	snp_B4219	snp_B4222	snp_B4231	snp_B4232	snp_B4234	snp_B4248	snp_B4263	snp_B4270
4	4	4	4	4	4	4	4	4	4	4	4	4	4	4	4	4
--	--	LG6	--	--	--	--	--	--	--	--	--	--	--	--	--	--
AA	AB	AA	AA	AB	AB	AB	AB	AB	AB	AA	AB	AB	AA	AA	AB	AB
AA	AB	AB	AB	AB	AB	AA	AB	AA	AB	AB	AA	AA	AA	AA	AA	AB
AA	AA	AA	AA	AA	AA	AA	AA	AB	AB	AA	AA	AA	AA	AB	AB	AA
AB	AB	AB	AB	AB	AB	AA	AB	AA	AB	AB	AA	AA	-	AA	AA	AB
AA	AA	AA	AA	AA	AA	AA	AA	AA	AA	AA	AB	AA	AA	AA	AB	AA
AA	AA	AA	AA	AA	-	AA	AA	-	AA	AA	AB	-	AB	AB	AB	-
AB	AB	AB	AB	AB	AB	AB	AB	AA	AA	AB	AA	AA	AB	AB	AA	AA
AA	AB	AA	AA	AA	AB	AA	AB	AB	AB	AB	AA	AA	AA	AA	AA	AA
AA	AB	AA	AA	AA	AA	AA	AA	AB	AB	AB	AB	AB	AA	AB	AB	AB
AB	AB	AB	AB	AB	AB	AB	AB	AA	AA	AA	AA	AA	AB	AA	AA	AB
AB	AB	AB	AB	AB	AB	AB	AB	AA	AA	AB	AA	AA	AA	AB	AA	AB
AB	AA	AA	AB	AB	AB	AA	AA	AB	AB	AB	AA	AB	AB	AB	AA	AA
AA	AB	AB	AB	AB	AB	AB	AB	AB	AA	AB	AB	AB	AB	AA	AB	AB
AB	AB	AB	AB	AB	AB	AA	AB	AB	AA	AB	AB	AA	AB	AA	AB	AA
AB	AB	AA	AA	AA	AB	AA	AA	AA	AA	AB	AB	AA	AA	AA	AA	AA
AB	AB	AB	AB	AB	AA	AB	AB	AA	AB	AB	AB	AA	AA	AB	AA	AA
AB	AB	AB	AB	AB	AB	AB	AB	AA	AA	AB	AA	AA	AA	AA	AA	AA
AA	AB	AA	AA	AA	-	AA	AA	-	AB	AA	AB	-	AB	AA	AB	-
AA	AB	AB	AB	AB	AB	AB	AB	AA	AB	AB	AA	AA	AA	AA	AA	AA
AB	AB	AB	AB	AB	AB	AA	AB	AB	AB	AB	AA	AA	AA	AA	AA	AA
AA	AB	AB	AB	AA	AB	AB	AB	AB	AB	AB	AB	AB	AB	AA	AB	AB
AB	AA	AB	AA	AB	AA	AB	AB	AB	AA	AA	AA	AB	AA	AA	AA	AB
-	AA	AA	AB	AA	-	AA	AA	-	AB	AA	AA	-	-	AA	AA	AB
AB	AB	AB	AB	AA	AB	AA	AA	AA	AA	AA	AA	AA	AA	AA	AA	AA
AB	AB	AB	AB	AB	AB	AB	AB	AA	AB	AB	AA	AB	AA	AA	AA	AB
AB	AA	AA	AA	AA	AA	AA	AA	AA	AA	AA	AA	AA	AA	AB	AA	AA
AA	AB	AB	AB	AB	AB	AB	AB	AB	AA	AB	AA	AA	AA	AA	AA	AA
AB	AA	AB	AB	AA	AA	AB	AB	AB	AA	AB	AA	AB	AB	AB	AB	AB
AA	AA	AA	AB	AA	AB	AB	AA	AA	AB	AA	AA	AB	AB	AB	AB	AA
-	AA	AA	AB	AA	-	AA	AA	-	AB	AB	AB	-	-	AB	AA	-
AA	AA	AA	AA	AA	AA	AA	AA	AB	AB	AA	AB	AB	AB	AB	AB	AB
AA	AB	AB	AB	AB	AB	AB	AB	AB	AB	AB	AB	AB	AB	AB	AB	AB
[illegible]	[illegible]	[illegible]	[illegible]	[illegible]	[illegible]	[illegible]	[illegible]	[illegible]	[illegible]	[illegible]	[illegible]	[illegible]	[illegible]	[illegible]	[illegible]	[illegible]

snp_B4289	snp_B4318	snp_B4326	snp_B4331	snp_B4334	snp_B4338	snp_B4343	snp_B4344	snp_B4365	snp_B4370	snp_B4371	snp_B4400	snp_B4408	snp_B4409	snp_B4428	snp_B4433	snp_B4438	snp_B4450	snp_B4453	snp_B4456	snp_B4459
4	4	4	4	4	4	4	4	4	4	4	4	4	4	4	4	4	4	4	4	4
--	--	--	--	LG6	--	--	--	--	--	LG6	--	--	--	--	--	--	--	--	--	--
AA	AA	AB	AA	AA	AB	AB	AA	AA	AB	AA	AA	AB	AB	AA	AB	AB	AA	AB	AB	AA
AA	AA	AA	AB	AB	AB	AA	AB	AA	AB	AB	AB	AA	AB	AB	AB	AB	AB	AA	AA	AB
AA	AA	AB	AB	AA	AB	AA	AA	AA	AB	AA	AB	AB	AA	AB	AA	AB	AB	AA	AB	AB
AA	AA	AA	AA	AB	-	AA	AB	AA	AB	AB	AB	AA	AB	-	AB	AB	-	AB	AB	AB
AA	AA	AA	AB	AA	AA	AB	AA	AA	AA	AA	AA	AB	AA	AA	AA	AA	AA	AB	AB	AA
AB	-	AA	AB	AA	AA	AB	AA	AB	AA	AA	AA	AB	-	AA	-	AA	-	AA	AA	AB
AB	AB	AA	AB	AB	AA	AA	AB	AB	AA	AB	AA	AB	AB	AB	AA	AA	AA	AA	AA	AB
AA	AA	AB	AA	AA	AB	AA	AB	AA	AA	AA	AB	AA	AA	AB	AB	AB	AA	AA	AA	AA
AA	AA	AB	AB	AA	AB	AB	AB	AA	AB	AA	AB	AA	AB	AA	AA	AB	AA	AB	AB	AB
AB	AA	AA	AB	AB	AA	AA	AA	AB	AA	AB	AA	AA	AA	AB	AB	AA	AB	AA	AB	AB
AB	AB	AA	AA	AB	AA	AA	AB	AB	AA	AB	AA	AB	AB	AB	AA	AA	AB	AA	AA	AA
AA	AB	AB	AB	AA	AB	AA	AA	AB	AB	AB	AB	AB	AB	AA	AA	AB	AB	AA	AA	AB
AB	AB	AB	AB	AB	AB	AB	AA	AB	AB	AB	AB	AB	AB	AB	AA	AB	AB	AA	AA	AB
AB	AA	AB	AB	AA	AB	AB	AB	AA	AA	AB	AA	AA	AB	AB	AB	AB	AA	AA	AA	AA
AB	AB	AA	AA	AB	AA	AB	AB	AB	AA	AB	AA	AA	AA	AB	AA	AA	AA	AB	AA	AA
AB	AB	AA	AB	AB	AB	AB	AB	AA	AB	AB	AB	AB	AB	AB	AB	AA	AB	AB	AB	AB
AA	AB	AA	AA	AB	AA	AA	AB	AA	AA	AB	AA	AA	AB	AB	AA	AA	AA	AB	AB	AA
AA	-	AB	AA	AB	-	AA	AB	AA	AB	AA	AB	AA	AA	AA	AB	AB	-	AA	AB	-
AA	AA	AB	AA	AB	AB	AA	AB	AA	AB	AA	AB	AA	AA	AB	AB	AB	AB	AA	AA	AA
AA	AA	AB	AA	AB	AB	AA	AB	AA	AB	AB	AB	AA	AB	AB	AB	AB	AB	AA	AB	AA
AB	AB	AB	AA	AB	AB	AB	AB	AA	AB	AB	AB	AA	AB	AB	AB	AB	AB	AB	AB	AB
AB	AB	AA	AA	AB	AA	AA	AB	AA	AA	AB	AB	AA	AB	AA	AA	AA	AB	AA	AA	AA
AA	AA	AB	-	AA	AB	AA	AA	AA	AB	AA	AB	AA	AA	-	-	AB	-	AB	AB	AA
AA	AA	AB	AA	AB	AB	AA	AB	AA	AA	AB	AA	AA	AB	AA	AA	AB	AB	AB	AB	AA
AB	AA	AB	AA	AB	AB	AA	AB	AA	AB	AB	AB	AA	AB	AB	AB	AB	AB	AA	AA	AA
AB	AA	AB	AB	AA	AA	AB	AA	AB	AA	AA	AA	AB	AA	AA	AA	AA	AA	AA	AB	AB
AA	AB	AB	AA	AB	AB	AA	AB	AA	AB	AB	AB	AA	AB	AB	AB	AB	AB	AA	AA	AA
AB	AA	AA	AB	AA	AA	AB	AA	AB	AA	AB	AA	AB	AB	AB	AA	AA	AB	AA	AA	AB
AA	AA	AB	AA	AA	AB	AB	AB	AB	AB	AA	AB	AB	AB	AB	AA	AB	AA	AB	AA	AA
AB	AB	AB	AB	AA	AB	AA	AA	AA	-	AA	AB	AB	-	AA	-	AB	-	AA	AA	-
AB	AA	AB	AB	AA	AB	AB	AA	AB	AB	AA	AB	AB	AA	AA	AB	AB	AB	AA	AA	AB
AB	AB	AB	AB	AB	AB	AB	AB	AB	AB	AB	AB	AB	AB	AB	AB	AB	AB	AA	AA	AB
[illegible]	[illegible]	[illegible]	[illegible]	[illegible]	[illegible]	[illegible]	[illegible]	[illegible]	[illegible]	[illegible]	[illegible]	[illegible]	[illegible]	[illegible]	[illegible]	[illegible]	[illegible]	[illegible]	[illegible]	[illegible]

snp_B4462	snp_B4494	snp_B4496	snp_B4511	snp_B4570	snp_B4579	snp_B4582	snp_B4597	snp_B4627	snp_B4654	snp_B4675	snp_B4691	snp_B4705	snp_B4728	snp_B4734	snp_B4739	snp_B4742	snp_B4773	snp_B4789	snp_B4800
4	4	4	4	4	4	4	4	4	4	4	4	4	4	4	4	4	4	4	4
--	--	--	--	--	--	--	--	--	--	--	--	--	--	--	--	--	--	--	--
AA	AA	AB	AA	AB	AB	AA	AA	AA	AB	AB	AA	AA	AA	AB	AA	AA	AB	AB	AB
AB	AB	AB	AB	AB	AB	AB	AB	AB	AB	AA	AB	AB	AB	AB	AB	AB	AA	AB	AB
AB	AB	AB	AA	AA	AB	AA	AA	AB	AB	AB	AA	AB	AA	AA	AA	AA	AB	AB	AB
-	AB	AB	AB	-	AB	AB	AB	AB	AB	AB	AB	AB	AB	AB	AB	AB	AA	AB	AB
AA	AA	AA	AA	AA	AA	AA	AA	AA	AA	AA	AA	AA	AA	AA	AA	AA	AA	AA	AA
-	AA	AA	-	AA	AA	-	AA	AA	AA	-	AA	AA	-	AA	AA	AB	AB	AA	AA
AA	AA	AA	AB	AB	AB	AB	AB	AA	AA	AA	AB	AB	AB	AB	AB	AB	AA	AA	AA
AB	AB	AB	AB	AA	AA	AA	AB	AB	AB	AB	AA	AB	AA	AB	AB	AA	AA	AB	AB
AB	AB	AB	AB	AA	AB	AA	AA	AA	AB	AB	AA	AB	AA	AA	AB	AA	AB	AB	AB
AA	AA	AA	AA	AB	AA	AB	AB	AA	AA	AB	AA	AA	AB	AA	AB	AB	AB	AA	AA
AA	AA	AA	AB	AB	AA	AB	AA	AA	AB	AA	AA	AA	AB	AA	AB	AB	AA	AA	AA
AB	AB	AB	AA	AB	AB	AB	AA	AB	AA	AB	AA	AB	AA	AA	AA	AB	AA	AB	AB
AA	AB	AA	AB	AB	AB	AB	AA	AA	AA	AA	AB	AA	AB	AA	AB	AB	AB	AB	AB
AA	AA	AA	AA	AB	AA	AB	AB	AA	AB	AB	AB	AB	AB	AB	AA	AB	AA	AB	AA
AA	AA	AA	AB	AB	AA	AB	AB	AA	AA	AA	AB	AA	AB	AA	AB	AB	AA	AA	AA
AB	AB	AB	AB	AB	AB	AB	AB	AB	AB	AB	AB	AB	AB	AB	AB	AA	AB	AB	AB
AA	AA	AA	AB	AB	AB	AB	AB	AA	AA	AA	AB	AB	AB	AB	AB	AA	AB	AA	AA
-	AB	-	AA	AB	AB	-	AA	AB	AA	-	AA	-	AB	AA	AB	AA	AB	AB	AB
AB	AB	AB	AB	AB	AA	AB	AB	AB	AB	AA	AA	AB	AA	AB	AB	AA	AA	AB	AB
AB	AB	AB	AB	AB	AB	AB	AB	AB	AB	AB	AB	AB	AA	AB	AB	AA	AB	AB	AB
AB	AB	AB	AB	AB	AB	AB	AB	AB	AB	AB	AB	AB	AB	AB	AB	AB	AB	AB	AB
AA	AA	AA	AA	AA	AB	AB	AB	AA	AB	AA	AB	AA	AA	AB	AA	AB	AA	AA	AA
-	AB	AB	-	-	AB	-	AB	AB	AA	-	-	AB	-	-	AA	AA	AB	AB	AB
AA	AA	AA	AA	AA	AB	AB	AB	AA	AB	AB	AB	AA	AA	AB	AA	AA	AA	AB	AA
AB	AB	AA	AB	AB	AB	AB	AB	AA	AB	AB	AB	AB	AA	AB	AB	AA	AA	AB	AB
AA	AA	AA	AA	AA	AB	AA	AA	AA	AA	AB	AB	AA	AB	AB	AA	AA	AB	AA	AA
AB	AB	AB	AB	AB	AB	AB	AB	AB	AB	AB	AA	AB	AA	AB	AB	AA	AA	AB	AB
AA	AA	AB	AB	AB	AA	AB	AA	AA	AA	AB	AA	AA	AA	AB	AB	AB	AA	AB	AA
AB	AB	AB	AB	AA	AA	AA	AA	AB	AB	AB	AB	AA	AA	AB	AB	AA	AB	AB	AB
-	AB	AB	-	-	AA	AB	AA	AB	AA	-	AA	AB	AA	AA	AA	AA	AB	AB	AB
AA	AB	AB	AA	AA	AA	AA	AA	AA	AB	AA	AA	AA	AA	AA	AA	AB	AA	AB	AB
AB	AB	AB	AB	AB	AB	AB	AB	AB	AB	AB	AA	AB	AA	AB	AB	AB	AA	AB	AB
[illegible]	[illegible]	[illegible]	[illegible]	[illegible]	[illegible]	[illegible]	[illegible]	[illegible]	[illegible]	[illegible]	[illegible]	[illegible]	[illegible]	[illegible]	[illegible]	[illegible]	[illegible]	[illegible]	[illegible]

snp_B4807	snp_B4837	snp_B4858	snp_B4893	snp_B4925	snp_B4935	snp_B4947	snp_B5007	snp_B5008	snp_B5009	snp_B5029	snp_B5031	snp_B5042	snp_B5046	snp_B5048	snp_B5052	snp_B5055	snp_B5063	snp_B5076
4	4	4	4	4	4	4	4	4	4	4	4	4	4	4	4	4	4	4
--	--	--	--	--	--	LG6	--	--	--	--	--	--	--	--	--	--	--	--
AB	AA	AA	AB	AB	AA	AA	AB	AB	AA	AB	AB	AA	AB	AA	AA	AB	AB	AB
AB	AB	AB	AA	AB	AB	AB	AB	AA	AB	AB	AB	AB	AB	AB	AB	AA	AB	AA
AB	AA	AA	AB	AB	AB	AA	AB	AB	AA	AB	AB	AA	AB	AA	AA	AB	AB	AB
AB	AB	AB	AA	AB	AB	AB	AB	AA	AB	AA	-	AB	AB	AB	-	-	AB	AA
AA	AA	AA	AA	AA	AA	AA	AA	AA	AA	AA	AA	AA	AB	AA	AA	AA	AA	AB
AA	AB	AA	AA	-	AA	AA	AB	AB	AA	-	-	-	AB	AA	AB	-	AA	AB
AA	AB	AB	AA	AB	AB	AB	AB	AA	AA	AA	AB	AA	AA	AB	AB	AA	AA	AA
AB	AA	AB	AA	AB	AB	AB	AA	AA	AB	AB	AB	AA	AB	AA	AA	AA	AB	AA
AB	AA	AA	AB	AB	AA	AA	AA	AB	AB	AA	AB	AA	AA	AB	AB	AB	AB	AB
AA	AB	AB	AA	AA	AB	AB	AA	AA	AA	AA	AA	AA	AA	AB	AB	AB	AB	AB
AA	AB	AB	AA	AB	AB	AB	AA	AA	AA	AA	AA	AA	AA	AA	AB	AA	AA	AA
AB	AB	AB	AB	AB	AA	AA	AB	AB	AA	AB	AA	AA	AB	AB	AB	AA	AB	AA
AA	AB	AB	AB	AB	AA	AA	AB	AB	AA	AB	AB	AB	AB	AB	AB	AB	AA	AB
AA	AB	AB	AB	AA	AB	AB	AB	AA	AA	AB	AA	AA	AB	AB	AB	-	AA	AA
AA	AA	AA	AA	AB	AB	AB	AA	AA	AB	AA	AA	AA	AA	AB	AA	AA	AA	AA
AB	AB	AB	AA	AA	AB	AB	AB	AB	AB	AA	AB	AB	AA	AB	AB	AB	AB	AB
AA	AA	AB	AA	AB	AB	AB	AA	AB	AA	AA	AB	AA	AA	AB	AA	AB	AA	AA
AB	AA	AA	AB	-	AA	AA	-	AA	AA	-	-	-	AB	-	-	AB	AB	AA
AB	AA	AB	AB	AB	AB	AB	AA	AA	AB	AB	AB	AB	AB	AB	AA	AA	AB	AA
AB	AA	AB	AB	AB	AB	AB	AA	AA	AB	AA	AB	AB	AB	AB	-	-	AB	AA
AB	AB	AB	AB	AB	AB	AB	AA	AB	AB	AB	AB	AB	AA	AB	AB	AB	AB	AB
AA	AB	AA	AA	AA	AA	AB	AA	AA	AB	AA	AA	AB	AA	AA	AA	AA	AB	AA
AB	AA	AB	AB	-	AA	AA	-	AA	AB	-	-	-	AB	AB	-	-	AB	-
AA	AA	AB	AB	AB	AA	AB	AA	AA	AB	AB	AB	AB	AB	AB	AA	AA	AB	AA
AB	AB	AB	AB	AB	AB	AB	AA	AA	AB	AB	AB	AB	AB	AB	AA	AA	AB	AA
AA	AB	AA	AA	AA	AA	AA	AB	AB	AA	AA	AA	AA	AB	AA	AB	AB	AA	AA
AB	AA	AB	AB	AB	AB	AB	AA	AA	AB	AB	AB	AB	AB	AB	AA	AA	AB	AA
AA	AB	AA	AB	AA	AA	AB	AB	AB	AA	AA	AA	AB	AA	AB	AB	AA	AB	AA
AB	AA	AB	AB	AB	AB	AA	AB	AB	AA	AB	AB	AB	AB	AA	-	AB	AB	AB
AB	AB	AB	AB	-	AA	AA	-	AA	AA	AB	-	AA	AB	AB	-	-	AB	AA
AA	AB	AA	AB	AA	AA	AA	AB	AB	AA	AB	AA	AB	AB	AA	AB	AA	AB	AB
AB	AB	AB	AB	AB	AB	AB	AB	AB	AB	AB	AB	AB	AB	AB	AB	AA	AB	AB
[illegible]	[illegible]	[illegible]	[illegible]	[illegible]	[illegible]	[illegible]	[illegible]	[illegible]	[illegible]	[illegible]	[illegible]	[illegible]	[illegible]	[illegible]	[illegible]	[illegible]	[illegible]	[illegible]

snp_B5086	snp_B5095	snp_B5103	snp_B5115	snp_B5146	snp_B5156	snp_B5157	snp_B5181	snp_B5184	snp_B5193	snp_B5202	snp_B5239	snp_B5240	snp_B5265	snp_B5279	snp_B5288	snp_B5328	snp_B5343	snp_B5358	snp_B5363
4	4	4	4	4	4	4	4	4	4	4	4	4	4	4	4	4	4	4	4
--	--	--	--	--	--	--	--	--	LG6	--	--	--	LG6	--	--	--	--	--	--
[illegible]	[illegible]	[illegible]	[illegible]	[illegible]	[illegible]	[illegible]	[illegible]	[illegible]	[illegible]	[illegible]	[illegible]	[illegible]	[illegible]	[illegible]	[illegible]	[illegible]	[illegible]	[illegible]	[illegible]

snp_B5368	snp_B5435	snp_B5458	snp_B5471	snp_B5484	snp_B5523	snp_B5524	snp_B5558	snp_B5562	snp_B5564	snp_B5596	snp_B5602	snp_B5620	snp_B5634	snp_B5639	snp_B5649	snp_B5652	snp_B5713	snp_B5727	snp_B5735
4	4	4	4	4	4	4	4	4	4	4	4	4	4	4	4	4	4	4	4
--	--	--	--	--	--	--	--	--	--	LG6	--	--	--	--	--	LG6	LG6	LG6	--
[illegible]	[illegible]	[illegible]	[illegible]	[illegible]	[illegible]	[illegible]	[illegible]	[illegible]	[illegible]	[illegible]	[illegible]	[illegible]	[illegible]	[illegible]	[illegible]	[illegible]	[illegible]	[illegible]	[illegible]

snp_B5762	snp_B5763	snp_B5769	snp_B5779	snp_B5816	snp_B5820	snp_B5828	snp_B5831	snp_B5835	snp_B5907	snp_B5914	snp_B5930	snp_B5935	snp_B5952	snp_B5954	snp_B5966	snp_B5992	snp_B6053	snp_B6162	snp_B6172
4	4	4	4	4	4	4	4	4	4	4	4	4	4	4	4	4	4	4	4
--	--	--	--	--	--	--	--	--	--	--	--	--	--	--	--	--	--	--	--
[illegible]	[illegible]	[illegible]	[illegible]	[illegible]	[illegible]	[illegible]	[illegible]	[illegible]	[illegible]	[illegible]	[illegible]	[illegible]	[illegible]	[illegible]	[illegible]	[illegible]	[illegible]	[illegible]	[illegible]

snp_B0001	snp_B0019	snp_B0070	snp_B0101	snp_B0105	snp_B0136	snp_B0156	snp_B0218	snp_B0219	snp_B0226	snp_B0237	snp_B0241	snp_B0243	snp_B0261	snp_B0262	snp_B0289	snp_B0322
5	5	5	5	5	5	5	5	5	5	5	5	5	5	5	5	5
--	--	--	--	--	--	--	--	--	--	--	--	--	--	--	--	--
[illegible]	[illegible]	[illegible]	[illegible]	[illegible]	[illegible]	[illegible]	[illegible]	[illegible]	[illegible]	[illegible]	[illegible]	[illegible]	[illegible]	[illegible]	[illegible]	[illegible]

snp_B0343	snp_B0348	snp_B0357	snp_B0370	snp_B0394	snp_B0428	snp_B0430	snp_B0437	snp_B0441	snp_B0444	snp_B0458	snp_B0466	snp_B0467	snp_B0486	snp_B0492	snp_B0506	snp_B0512	snp_B0514	snp_B0519	snp_B0583	snp_B0604	snp_B0616	snp_B0628	snp_B0633	snp_B0643	snp_B0675	snp_B0724	snp_B0729	snp_B0736	snp_B0765	snp_B0789	snp_B0812	snp_B0829	snp_B0839	snp_B0844	snp_B0857	snp_B0860	snp_B0882	snp_B0895	snp_B0934	snp_B0995	snp_B1065	snp_B1091	snp_B1096	snp_B1138	snp_B1157	snp_B1164	snp_B1183	snp_B1184	snp_B1215	snp_B1226	snp_B1240	snp_B1243	snp_B1256	snp_B1263	snp_B1294	snp_B1295	snp_B1300	snp_B1317	snp_B1339	snp_B1349	snp_B1368	snp_B1403	snp_B1406	snp_B1410	snp_B1476	snp_B1479	snp_B1483	snp_B1488	snp_B1497	snp_B1504	snp_B1517	snp_B1518	snp_B1536	snp_B1539	snp_B1540	snp_B1551
5	5	5	5	5	5	5	5	5	5	5	5	5	5	5	5	5	5	5	5	5	5	5	5	5	5	5	5	5	5	5	5	5	5	5	5	5	5	5	5	5	5	5	5	5	5	5	5	5	5	5	5	5	5	5	5	5	5	5	5	5	5	5	5	5	5	5	5	5	5	5	5	5	5	5	5	5
--	--	--	--	--	--	--	--	LG13	--	--	--	--	--	--	--	--	--	--	--	--	--	--	--	--	--	--	--	--	--	--	--	--	--	--	--	--	--	--	--	--	--	--	--	--	--	--	--	--	--	--	--	--	--	LG13	--	--	--	--	--	--	--	--	--	--	--	--	--	--	--	--	--	--	--	--	--	--
AA	AA	AB	AA	AB	AB	AB	AB	AB	AB	AB	AA	AA	AB	AB	AB	AB	AB	AA	AA	AA	AB	AB	AB	AB	AB	AA	AB	AB	AA	AB	AB	AA	AB	AB	AB	AB	AA	AB	AB	AA	AA	AA	AB	AA	AA	AB	AA	AB	AA	AB	AA	AB	AA	AA	AA	AB	AB	AB	AA	AA	AB	AA	AA	AA	AB	AB	AA	AA	AB	AB	AB	AB	AB	AA	AA	AB

snp_B1556 5	snp_B1594 5	snp_B1608 5	snp_B1618 5	snp_B1656 5	snp_B1657 5	snp_B1658 5	snp_B1676 5	snp_B1702 5	snp_B1721 5	snp_B1745 5	snp_B1761 5	snp_B1768 5	snp_B1776 5	snp_B1779 5	snp_B1808 5	snp_B1812 5	snp_B1820 5	snp_B1845 5	snp_B1864 5	snp_B1889 5	snp_B1892 5	snp_B1901
--	--	--	--	--	--	--	--	--	--	--	--	--	--	--	--	--	--	--	--	--	--	--
AA	AA	AB	AA	AB	AB	AA	AB	AB	AA	AB	AB	AB	AB	AB	AB	AB	AA	AA	AB	AB	AA	AA
AA	AB	AA	AA	AA	AA	AA	AA	AA	AB	AB	AB	AA	AA	AA	AA	AB	AA	AB	AA	AA	AA	AB
AA	AB	AB	AA	AB	AB	AA	AA	AB	AB	AA	AA	AA	AB	AB	AB	AA	AA	AB	AB	AB	AB	AA
AA	-	AA	AA	-	AB	AB	AB	AA	AB	AB	-	AA	AB	AA	-	AB	AB	AA	AB	AB	AB	AA
AA	AA	AB	AA	AB	AA	AA	AB	AB	AB	AB	AB	AB	AB	AB	AA	AA	AA	AB	AA	AA	AA	AB
-	-	AB	AA	AB	AB	-	AB	AB	AA	-	-	AB	-	AA	AB	-	-	AA	-	AB	AA	AA
AB	AA	AB	AB	-	AB	AB	AB	AA	AA	-	AB	AB	AB	AB	AB	AB	AB	AA	AB	AB	AB	AB
AA	AA	AA	AA	AA	AA	AA	AA	AA	AB	AB	AA	AB	AA	AA	AA	AB	AA	AA	AA	AA	AA	AA
AA	AB	AB	AA	AB	AB	AA	AA	AB	AB	AB	AA	AB	AB	AB	AB	AA	AA	AB	AB	AB	AB	AB
AB	AA	AA	AB	AB	AA	AA	AA	AB	AB	AB	AA	AB	AA	AB	AA	AB	AB	AA	AB	AA	AA	AA
AA	AB	AB	AB	AB	AB	AB	AB	AB	AA	AB	AA	AB	AB	AB	AB	AA	AA	AA	AB	AB	AA	AA
AB	AB	AB	AB	AA	AA	AB	AB	AB	AA	AB	AA	AA	AA	AB	AA	AA	AB	AA	AB	AB	AB	AB
AA	AB	AA	AA	AA	AA	AA	AA	AB	AB	AB	AB	AA	AA	AA	AA	AB	AA	AB	AA	AA	AA	AB
AA	AB	AA	AB	AB	AB	AB	AB	AB	AA	AB	AA	AB	AB	AB	AB	AA	AA	AA	AB	AA	AA	AA
AB	AA	AB	AB	AB	AB	AA	AA	AB	AA	AA	AB	AB	AA	AB	AB	AA	AB	AA	AB	AB	AB	AA
AA	AB	AB	AA	AB	AB	AB	AB	AB	AA	AA	AB	AA	AB	AA	AB	AA	AB	AB	AB	AB	AB	AB
AA	AA	AA	AA	AA	AA	AA	AA	AA	AA	AA	AA	AA	AA	AA	AA	AA	AA	AA	AA	AA	AA	AA
-	AB	AA	AB	-	AA	-	AA	AB	-	-	-	AB	-	AA	AA	-	AB	AA	-	AA	-	-
AB	AB	AB	AB	AB	AB	AB	AB	AA	AB	AB	AB	AB	AB	AA	AB	AB	AB	AB	AB	AB	AB	AB
AB	AB	AA	AB	AB	AA	AA	AA	AB	AB	AB	AA	AB	AA	AB	AA	AA	AA	AB	AB	AA	AA	-

snp_B2693	snp_B2703	snp_B2747	snp_B2761	snp_B2778	snp_B2788	snp_B2791	snp_B2813	snp_B2827	snp_B2831	snp_B2837	snp_B2842	snp_B2850	snp_B2869	snp_B2880	snp_B2881	snp_B2897	snp_B2898	snp_B2921	snp_B2924	snp_B2930	snp_B2931	snp_B2948	snp_B2962	snp_B2972	snp_B3001	snp_B3023	snp_B3029
5	5	5	5	5	5	5	5	5	5	5	5	5	5	5	5	5	5	5	5	5	5	5	5	5	5	5	5
--	--	--	--	--	--	--	--	--	LG13	--	--	--	--	--	--	--	--	--	--	--	--	--	--	--	--	--	--

snp_B3067	snp_B3083	snp_B3094	snp_B3099	snp_B3114	snp_B3187	snp_B3197	snp_B3212	snp_B3220	snp_B3240	snp_B3251	snp_B3254	snp_B3271	snp_B3273	snp_B3283	snp_B3293	snp_B3298	snp_B3307	snp_B3308	snp_B3335	snp_B3344	snp_B3346	snp_B3361	snp_B3363	snp_B3368	snp_B3379	snp_B3382
5	5	5	5	5	5	5	5	5	5	5	5	5	5	5	5	5	5	5	5	5	5	5	5	5	5	5
--	--	--	--	--	--	--	--	--	--	--	--	--	--	--	--	--	--	--	--	--	--	--	--	--	--	--

snp_B3383	snp_B3395	snp_B3406	snp_B3409	snp_B3433	snp_B3453	snp_B3495	snp_B3498	snp_B3510	snp_B3530	snp_B3534	snp_B3556	snp_B3559	snp_B3561	snp_B3569	snp_B3578	snp_B3585	snp_B3615	snp_B3635	snp_B3636	snp_B3653	snp_B3655
5	5	5	5	5	5	5	5	5	5	5	5	5	5	5	5	5	5	5	5	5	5
--	--	--	--	--	--	--	--	--	--	--	--	--	--	--	--	--	--	--	--	--	--

snp_B36935	snp_B38535	snp_B38715	snp_B38935	snp_B39055	snp_B39345	snp_B39355	snp_B39415	snp_B39445	snp_B39735	snp_B39815	snp_B40005	snp_B40015	snp_B40355	snp_B40565	snp_B40625	snp_B40765	snp_B40795	snp_B40845	snp_B40985	snp_B41125	snp_B41185	snp_B41515	snp_B41535	snp_B41805	snp_B41875	snp_B42245	snp_B42405	snp_B42825	snp_B42845	snp_B43365	snp_B43695	snp_B43935	snp_B43965	snp_B44115	snp_B44185	snp_B44375	snp_B44405	snp_B44465	snp_B44715	snp_B44775	snp_B44815	snp_B44825	snp_B45065	snp_B45235	snp_B45255	snp_B45365	snp_B45605	snp_B45895	snp_B46075	snp_B46235	snp_B46445	snp_B46625	snp_B46645	snp_B46865	snp_B46935	snp_B47115	snp_B47865	snp_B48015	snp_B48125	snp_B48395	snp_B49565	snp_B49605	snp_B49685	snp_B50035	snp_B50445	snp_B50455	snp_B50505	snp_B50605	snp_B50695	snp_B50875	snp_B51385	snp_B51425	snp_B51445	snp_B51975	snp_B52045	snp_B52145
--	--	--	--	--	--	--	--	--	--	--	--	--	--	--	--	--	--	--	--	--	--	--	--	--	--	--	--	--	--	--	--	--	--	--	--	--	--	--	--	LG13	--	--	--	--	--	--	--	--	--	--	--	--	--	--	--	--	--	--	--	--	--	--	--	--	--	--	--	--	--	--	--	--	--	--	--	--

snp_B5220 5	snp_B5236 5	snp_B5262 5	snp_B5296 5	snp_B5316 5	snp_B5326 5	snp_B5340 5	snp_B5381 5	snp_B5384 5	snp_B5414 5	snp_B5449 5	snp_B5468 5	snp_B5479 5	snp_B5497 5	snp_B5623 5	snp_B5635 5	snp_B5641 5	snp_B5645 5	snp_B5647 5	snp_B5660 5	snp_B5662 5	snp_B5669 5	snp_B5706 5	snp_B5722 5	snp_B5729 5	snp_B5733 5
--	--	--	LG13	--	--	--	--	--	--	--	--	--	--	--	--	--	--	--	--	--	--	--	LG13	--	--
AA	AA	AA	AA	AB	AA	AB	AA	AB	AA	AB	AB	AB	AA	AB	AB	AB	AB	AA	AB	AB	AA	AB	AB	AA	AB

snp_B5734 5	snp_B5737 5	snp_B5751 5	snp_B5756 5	snp_B5760 5	snp_B5788 5	snp_B5790 5	snp_B5792 5	snp_B5802 5	snp_B5810 5	snp_B5827 5	snp_B5834 5	snp_B5852 5	snp_B5854 5	snp_B5866 5	snp_B5872 5	snp_B5874 5	snp_B5916 5	snp_B5967 5	snp_B5999 5	snp_B6014 5	snp_B6095 5	snp_B6120 5	snp_B6165 5	snp_B0004 6	snp_B0021 6
--	--	--	--	--	LG13	--	--	--	--	--	--	--	--	--	--	--	--	--	--	--	--	--	--	--	--
AA	AA	AA	AA	AB	AB	AB	AA	AA	AB	AB	AA	AA	AB	AA	AA	AA	AA	AA	AA	AB	AA	AB	AA	AA	AA

snp_B0023 6	snp_B0041 6	snp_B0046 6	snp_B0052 6	snp_B0053 6	snp_B0112 6	snp_B0120 6	snp_B0140 6	snp_B0142 6	snp_B0150 6	snp_B0154 6	snp_B0155 6	snp_B0162 6	snp_B0163 6	snp_B0168 6	snp_B0269 6	snp_B0323 6	snp_B0382 6	snp_B0393 6	snp_B0453 6	snp_B0488 6	snp_B0493 6	snp_B0508 6	snp_B0525 6	snp_B0526 6
--	--	--	--	--	--	--	--	--	--	--	--	--	--	--	--	--	--	--	--	--	--	--	--	--
AA	AA	AB	AA	AA	AB	AA	AA	AA	AB	AA	AA	AA	AA	AA	AB	AA	AA	AA	AA	AA	AA	AB	AB	AB

snp_B0542	snp_B0547	snp_B0560	snp_B0618	snp_B0694	snp_B0698	snp_B0723	snp_B0728	snp_B0810	snp_B0836	snp_B0867	snp_B0875	snp_B0911	snp_B0921	snp_B0955	snp_B0956	snp_B0962	snp_B0982	snp_B1018	snp_B1031	snp_B1038	snp_B1040	snp_B1092	snp_B1106	snp_B1113	snp_B1119	snp_B1139	snp_B1155	snp_B1180	snp_B1186	snp_B1194	snp_B1212	snp_B1254	snp_B1255	snp_B1289	snp_B1296	snp_B1313	snp_B1314	snp_B1325	snp_B1367	snp_B1377	snp_B1404	snp_B1423	snp_B1439	snp_B1443	snp_B1444	snp_B1460	snp_B1462	snp_B1478	snp_B1496	snp_B1503	snp_B1514	snp_B1546	snp_B1560	snp_B1595	snp_B1606	snp_B1609	snp_B1625	snp_B1630	snp_B1677	snp_B1698	snp_B1707	snp_B1765	snp_B1804	snp_B1810	snp_B1836	snp_B1877	snp_B1909	snp_B1914	snp_B1916	snp_B1930	snp_B1932	snp_B1949	snp_B1995	snp_B1998	snp_B2010	snp_B2011
6	6	6	6	6	6	6	6	6	6	6	6	6	6	6	6	6	6	6	6	6	6	6	6	6	6	6	6	6	6	6	6	6	6	6	6	6	6	6	6	6	6	6	6	6	6	6	6	6	6	6	6	6	6	6	6	6	6	6	6	6	6	6	6	6	6	6	6	6	6	6	6	6	6	6	6	6
--	--	--	--	LG8	--	--	--	--	--	--	--	--	--	--	--	LG8	--	--	--	LG8	--	--	--	--	--	--	--	--	--	--	--	--	LG8	--	--	--	--	--	--	--	--	--	LG8	--	--	--	--	--	--	--	--	--	--	--	--	--	--	--	--	--	--	--	--	--	--	--	--	--	--	--	--	--	--	--	--	--
AA	AA	AB	AA	AB	AA	AA	AB	AA	AA	AA	AA	AA	AA	AA	AA	AB	AB	AA	AB	AB	AA	AB	AB	AA	AB	AB	AA	AB	AB	AA	AA	AB	AB	AB	AA	AB	AA	AA	AA	AA	AB	AA	AB	AA	AA	AB	AA	AB	AB	AB	AB	AB	AA	AB	AA	AA	AA	AB	AB	AB	AA	AB	AA	AA	AB	AB	AA	AA	AA	AA	AB	AB	AB	AA	AB	AA

snp_B2053	snp_B2057	snp_B2062	snp_B2074	snp_B2079	snp_B2090	snp_B2098	snp_B2101	snp_B2109	snp_B2121	snp_B2133	snp_B2134	snp_B2147	snp_B2150	snp_B2159	snp_B2172	snp_B2182
6	6	6	6	6	6	6	6	6	6	6	6	6	6	6	6	6
--	--	--	--	--	--	LG8	--	--	--	LG8	--	--	--	--	--	--
AA	AB	AB	AA	AA	AB	AB	AB	AA	AA	AB	AA	AA	AA	AB	AA	AA
AA	AA	AB	AA	AA	AA	AB	AA	AA	AA	AB	AA	AB	AA	AA	AA	AA
AB	AB	AA	AB	AB	AB	AA	AA	AB	AB	AA	AB	AB	AB	AA	AA	AB
AB	AB	AA	AA	AB	AB	AA	-	AB	AB	AA	AB	AA	AB	AA	AA	AA
AA	AA	AB	-	AB	AB	AB	AB	AB	AB	AB	AA	AA	AA	AA	AB	AB
AA	AA	-	AA	AA	AB	AA	-	-	-	AA	-	-	AA	AA	AA	-
AB	AB	AB	AA	AB	AA	AA	AB	-	AA	AB	AA	-	AB	AA	AA	AA
AB	AB	AA	AA	AB	AB	AA	AA	AB	AB	AA	AB	AA	AB	AB	AA	AB
AA	AA	AA	AA	AB	AB	AB	AB	AA	AA	AB	AB	AA	AB	AB	AB	AA
AB	AA	AB	AA	AB	AA	AA	AA	AA	AB	AA	AA	AB	AB	AB	AB	AA
AB	AB	AB	AB	AB	AA	AA	AA	AB	AB	AB	AA	AB	AB	AA	AA	AB
AB	AB	AA	AB	AA	AA	AB	AB	AA	AB	AB	AB	AB	AB	AB	AB	AA
AB	AB	AA	AA	AB	AA	AB	AA	AA	AA	AB	AB	AB	AB	AA	AA	AA
AA	AB	AA	AB	AB	AA	AB	AA	AA	AB	AA	AB	AB	AA	AA	AB	AA
AB	AA	AA	AB	AB	AB	AA	AB	AB	AB	AA	AB	AB	AB	AB	AA	AB
AB	AA	AA	AB	AB	AB	AA	AB	AB	AB	AA	AA	AB	AB	AB	AB	AB
AA	AB	AA	AA	AB	AA	AA	AA	AB	AA	AA	AA	AA	AA	AA	AA	AA
-	AB	-	-	AB	-	-	-	-	AA	-	-	AA	AA	AB	-	AA
AB	AB	AB	AA	AB	AB	AB	AB	AB	AB	AB	AB	AB	AB	AB	AB	AB
AB	AB	AB	-	AB	AA	AB	AB	AA	AB	AB	AB	AB	AB	AA	-	AA
AB	AA	AB	AB	AB	AB	AB	AA	AB	AB	AB	AB	AB	AB	AB	AB	AB
AA	AB	AB	AB	AA	AA	AB	AB	AA	AB	AB	AB	AA	AB	AB	AB	AA
AA	AA	AB	-	AA	AB	AA	-	-	AB	-	AA	-	AB	AA	-	-
AA	AB	AB	AB	AA	AB	AB	AA	AB	AB	AB	AB	AA	AB	AB	AB	AB
AB	AA	AA	AA	AB	AA	AA	AB	AA	AA	AA	AA	AB	AA	AA	AA	AA
AA	AA	AB	AB	AA	AA	AB	AA	AA	AB	AB	AB	AA	AB	AB	AB	AA
AB	AB	AA	AA	AB	AA	AA	AA	AB	AB	AA	AB	AB	AB	AB	AA	AB
AB	AB	AA	AA	AB	AA	AA	AA	AB	AB	AA	AB	AB	AB	AA	AA	AB
AB	AB	AA	AA	AB	AA	AB	-	-	AB	AA	AA	AB	AB	AA	-	AB
AA	AA	AB	AA	AB	AB	AA	-	-	AB	AA	-	-	AA	AB	-	AB
AB	AB	AA	AA	AB	AA	AB	AA	AA	AA	AB	AB	AB	AA	AA	AA	AA
AA	AA	AB	AA	AA	AA	AB	AB	AA	AA	AA	AB	AA	AA	AA	AB	AA
AA	AA	AA	AA	AA	AB	-	AA	AB	AB	-	AB	AA	AB	AB	AB	AB
AA	AA	AA	AA	AA	AA	AA	AB	-	AA	AA	AA	-	AA	AA	-	-
AB	AA	-	AA	AB	AB	AA	AB	-	-	AA	AA	-	AB	AA	AA	-
AB	AB	AB	-	AB	AB	AB	AB	AB	AB	AB	AB	AB	AB	AB	-	AB
AB	AB	AB	AB	AB	AB	AB	AB	AB	AB	AB	AB	AB	AB	AB	AB	AB
AB	AB	AB	AA	AB	AB	AB	AB	AB	AB	AB	AB	AB	AB	AB	AB	AB
AA	AA	AA	AA	AA	AA	AA	AA	AA	AA	AA	AA	AA	AA	AA	AA	AA
AB	AB	AA	-	AA	AB	AB	AA	AA	AA	AB	-	AB	AB	AB	-	AB
AB	AA	AA	AA	AB	AB	AA	AB	AB	AB	AA	AA	AB	AB	AA	AA	AB
AB	AB	AA	AB	AB	AB	AA	AB	AA	AB	AB	AA	AB	AB	AA	AB	AA
AA	AA	AA	AB	AA	AB	AA	AA	AA	AB	AB	AA	AB	AB	AA	AA	AB
AA	AB	AB	AA	AB	AA	AA	AB	AA	AA	AA	AA	-	AA	AA	AA	-
AA	AA	AB	AA	AA	AB	AB	AB	AB	AA	AB	AB	AA	AA	AA	AA	AA
AA	AA	AB	AA	AA	AB	AB	AB	AA	AA	AB	AA	AA	AA	AB	AB	AA
AA	AA	AB	AA	AA	AB	AB	AA	AB	AB	AB	AA	AA	AA	AB	AA	AB
AA	AA	AA	AA	AA	AB	AA	AA	AB	AA	AA	AA	AA	AA	AA	AA	AB
AA	AA	AA	AA	AA	AA	AA	AA	AA	AA	AA	AA	AA	AA	AA	AA	AB
AA	AA	AA	AA	AA	AA	AA	AA	AA	AA	AA	AA	AA	AA	AA	AA	AA
AB	AB	AA	AB	AB	AB	AA	AA	AB	AB	AA	AA	AA	AA	AB	AB	AB

snp_B3258 6	snp_B3269 6	snp_B3339 6	snp_B3353 6	snp_B3377 6	snp_B3394 6	snp_B3400 6	snp_B3420 6	snp_B3439 6	snp_B3443 6	snp_B3462 6	snp_B3520 6	snp_B3525 6	snp_B3527 6	snp_B3576 6	snp_B3592 6	snp_B3597 6	snp_B3600 6	snp_B3627 6	snp_B3639 6	snp_B3670 6	snp_B3681 6	snp_B3692 6	snp_B3702 6	snp_B3706 6	snp_B3708 6	snp_B3746 6	snp_B3804 6	snp_B3805 6	snp_B3806 6	snp_B3820 6	snp_B3821 6	snp_B3843 6	snp_B3874 6	snp_B3877 6	snp_B3882 6	snp_B3886 6	snp_B3907 6	snp_B3917 6	snp_B3928 6	snp_B3938 6	snp_B3972 6	snp_B3996 6	snp_B3999 6	snp_B4018 6	snp_B4027 6	snp_B4052 6	snp_B4058 6	snp_B4086 6	snp_B4094 6	snp_B4097 6	snp_B4106 6	snp_B4114 6	snp_B4117 6	snp_B4130 6	snp_B4145 6	snp_B4146 6	snp_B4148 6	snp_B4159 6	snp_B4167 6	snp_B4189 6	snp_B4191 6	snp_B4201 6	snp_B4205 6	snp_B4218 6	snp_B4238 6	snp_B4247 6	snp_B4250 6	snp_B4266 6	snp_B4294 6	snp_B4312 6	snp_B4335 6	snp_B4348 6	snp_B4353 6	snp_B4358 6	snp_B4381 6	snp_B4397 6
--	--	--	--	--	--	--	--	--	--	--	--	--	--	--	--	--	--	--	--	--	--	--	--	--	--	--	--	--	--	--	--	--	--	--	--	LG8	--	--	--	LG8	LG8	--	--	--	--	--	--	--	--	--	--	--	--	--	--	--	--	--	--	--	--	LG8	--	--	--	--	--	--	--	--	--	--	--	--	--	--
AA	AA	AA	AB	AB	AA	AA	AB	AA	AB	AA	AA	AB	AB	AB	AA	AA	AB	AA	AB	AB	AA	AB	AA	AA	AA	AA	AB	AA	AA	AB	AB	AB	AA	AA	AB	AB	AA	AB	AB	AB	AB	AA	AA	AB	AB	AA	AA	AB	AA	AB	AA	AB	AB	AA	AB	AA	AA	AA	AA	AA	AA	AB	AB	AA	AA	AB	AB	AB	AB	AB	AB	AA	AB	AA	AA	AB

snp_B4405	snp_B4420	snp_B4422	snp_B4425	snp_B4427	snp_B4430	snp_B4442	snp_B4445	snp_B4451	snp_B4455	snp_B4466	snp_B4474	snp_B4514	snp_B4526	snp_B4535	snp_B4541	snp_B4554
6	6	6	6	6	6	6	6	6	6	6	6	6	6	6	6	6
--	--	--	--	--	--	--	LG8	--	--	--	--	--	--	--	--	--
AA	AA	AA	AA	AA	AA	AB	AB	AA	AA	AA	AA	AB	AA	AA	AA	AB
AA	AA	AA	AB	AA	AA	AA	AB	AA	AA	AA	AB	AB	AA	AA	AA	AB
AB	AB	AB	AA	AB	AB	AA	AA	AA	AB	AB	AB	AB	AA	AB	AB	AA
AB	AB	AB	-	AB	AB	AA	-	AA	AB	AB	AA	AA	AB	-	AB	AA
AB	AB	AB	AB	AB	AA	AB	AB	AB	AA	AA	AA	AB	AB	AB	AB	AB
AB	-	AB	AB	AB	AA	AB	-	AA	AA	AA	AA	AA	-	-	-	-
AA	AA	AB	AB	AA	AA	AB	AA	AA	AA	AB	AB	AB	AB	AA	AA	AB
AB	AB	AB	AA	AB	AB	AA	AA	AB	AB	AB	AA	AA	AB	AB	AB	AA
AA	AA	AA	AA	AA	AA	AB	AB	AB	AB	AA	AB	AA	AB	AA	AA	AB
AA	AA	AA	AA	AB	AA	AA	AB	AB	AA	AB	AB	AA	AA	AA	AA	AA
AB	AB	AB	AA	AB	AB	AA	AA	AA	AB	AB	AB	AA	AB	AB	AB	AA
AA	AA	AA	AB	AA	AB	AA	AB	AB	AB	AB	AB	AA	AA	AA	AB	AB
AA	AA	AA	AB	AA	AA	AA	AB	AA	AA	AB	AA	AA	AB	AA	AA	AB
AA	AA	AA	AA	AA	AA	AA	AB	AB	AA	AB	AA	AA	AA	AA	AA	AB
AA	AB	AB	AB	AB	AB	AA	AA	AA	AB	AB	AA	AA	AB	AB	AB	AA
AB	AB	AB	AA	AB	AB	AA	AB	AB	AB	AB	AB	AA	AA	AB	AB	AA
AA	AA	AA	AB	AA	AA	AA	AA	AA	AA	AA	AB	AB	AA	AA	AA	AA
AB	-	-	AA	AA	AA	AB	-	AA	-	AA	AA	AA	-	-	AA	-
AB	AB	AB	AB	AB	AB	AB	AB	AB	AB	AB	AB	AB	AB	AB	AB	AB
AA	AA	AA	AB	AA	AA	AB	AB	AB	AA	AB	AB	AB	AB	AA	AA	AB
AB	AB	AB	AB	AB	AB	AB	AB	AB	AB	AB	AB	AA	AA	AB	AB	AB
AA	AA	AA	AB	AA	AA	AA	AB	AB	AA	AA	AA	AB	AA	AA	AA	AB
-	-	-	-	AB	AA	AB	AA	AA	-	AA	AA	-	-	-	AB	-
AB	AB	AB	AB	AB	AB	AA	AB	AB	AB	AA	AA	AB	AA	AB	AA	AB
AA	AA	AA	AB	AA	AA	AA	AA	AB	AB	AB	AB	AA	AB	AB	AA	AA
AA	AA	AA	AB	AA	AA	AB	AA	AB	AA	AB	AB	AB	AB	AB	AA	AB
AB	AB	AB	AA	AB	AB	AA	AA	AA	AB	AB	AB	AB	AB	AB	AA	AA
AB	AB	AB	AB	AB	AB	AB	AA	AA	AB	AB	AB	AB	AB	AB	AA	AA
AA	AA	AA	AB	AA	AB	AA	AA	AB	AB	AB	AB	AA	AB	AB	AB	AA
-	AB	AB	AA	AA	AB	AB	AA	AA	AB	AB	AA	AB	AB	AB	-	AB
AA	AA	AA	AB	AA	AA	AA	AB	AB	AA	AB	AB	AA	AA	AA	AA	AA
AA	AA	AA	AA	AA	AA	AB	AB	AB	AA	AA	AB	AA	AB	AA	AA	AB
AB	AB	-	AB	AB	AB	AB	-	AB	AB	AA	AA	AA	AA	AB	AB	AA
AA	AA	AA	AB	AA	AA	AB	AA	AA	AA	AA	AA	AA	AA	AA	AA	-
-	-	AB	AA	-	AB	AA	-	AA	AB	AB	AB	AA	-	AA	AB	-
AB	AB	AB	AB	AB	AB	AB	-	AB	AB	AB	AB	AB	AB	AB	AB	AB
AB	AB	AB	AA	AB	AB	AB	AB	AB	AB	AB	AB	AB	AB	AB	AB	AB
AB	AB	AB	AB	AB	AB	AB	AB	AB	AB	AB	AB	AB	AB	AB	AB	AB
AA	AA	AA	AA	AA	AA	AB	AA	AA	AA	AA	AA	AB	AA	AA	AA	AA
AA	AB	AA	AA	AA	AB	AA	-	AB	AB	AB	AB	AA	AA	AB	AB	-
AB	AB	AB	AA	AB	AB	AA	AB	AA	AB	AB	AB	AA	AA	AA	AB	AA

snp_B55526	snp_B55556	snp_B55566	snp_B55636	snp_B55656	snp_B55786	snp_B55796	snp_B56326	snp_B56566	snp_B56636	snp_B56766	snp_B56826	snp_B57086	snp_B57426	snp_B57686	snp_B57846	snp_B57896	snp_B57956	snp_B57976	snp_B57986	snp_B58056	snp_B58376	snp_B584[illegible]
--	--	--	--	--	--	--	--	--	LG8	--	--	--	--	LG8	--	--	--	--	--	--	--	--
AB	AA	AB	AB	AA	AA	AA	AA	AA	AB	AA	AA	AA	AA	AA	AA	AA	AB	AB	AB	AA	AA	AA
AB	AA	AB	AA	AA	AB	AA	AA	AA	AB	AA	AA	AA	AA	AA	AA	AA	AB	AA	AA	AA	AA	AA
AA	AA	AA	AA	AB	AB	AA	AB	AB	AA	AB	AB	AA	AA	AB	AA	AB	AA	AB	AA	AB	AB	AB
AA	-	AB	AA	AB	AA	AB	AB	AB	AA	AB	AB	AA	AA	AB	AA	AB	AA	AB	AB	AB	AB	AB
AB	AB	AB	AB	AB	AA	AA	AA	AA	AB	AB	AB	AB	AB	AA	AB	AB	AB	AB	AB	AA	AA	AB
AA	AA	-	AA	AB	AA	AA	AA	AA	AA	AB	AA	-	AB	AB	AB	AA	AA	AA	-	-	AA	AB
AB	-	AB	AA	AA	AB	AB	AB	AB	AB	AA	AB	AA	AB	AB	AB	AA	AB	AA	AA	AA	AA	AA
AA	AB	AA	AB	AB	AA	AB	AB	AB	AB	AB	AB	AB	AA	AB	AA	AB	AB	AA	AB	AB	AB	AB
AB	AB	AA	AB	AA	AB	AB	AB	AB	AB	AB	AB	AA	AB	AA	AB	AA	AA	AB	AB	AA	AA	AA
AB	AA	AA	AB	AA	AA	AB	AA	AB	AA	AA	AA	AA	AA	AA	AA	AA	AB	AA	AA	AB	AA	AA
AA	AB	AA	AA	AB	AA	AA	AB	AB	AB	AB	AB	AB	AA	AB	AA	AB	AB	AA	AB	AB	AB	AA
AB	AB	AA	AB	AB	AA	AA	AB	AA	AB	AB	AB	AB	AA	AA	AA	AA	AB	AB	AB	AB	AB	AA
AB	AA	AB	AA	AA	AA	AB	AB	AB	AB	AA	AB	AA	AA	AA	AA	AA	AA	AA	AA	AB	AA	AA
AB	AA	AA	AB	AA	AB	AA	AA	AB	AA	AB	AA	AB	AA	AB	AA	AB	AB	AB	AB	AA	AA	AA
AA	AA	AA	AA	AB	AA	AA	AB	AB	AA	AB	AA	AB	AA	AB	AA	AA	AB	AB	AB	AB	AB	AB
AA	AB	AB	AB	AB	AB	AB	AA	AB	AA	AB	AA	AB	AA	AA	AA	AB	AB	AB	AB	AB	AB	AB
AA	AA	AA	AA	AA	AA	AA	AA	AA	AA	AA	AB	AA	AB	AB	AB	AA	AA	AA	AA	AA	AA	AA
AA	AB	-	AB	AA	-	AA	AA	AA	-	-	AB	-	AB	-	AB	-	-	-	-	-	AA	AA
AB	AB	AB	AB	AB	AB	AB	AB	AB	AB	AB	AB	AB	AB	AB	AB	AB	AB	AB	AB	AB	AB	AB
AB	-	AB	AB	AA	AB	AB	AB	AB	-	AB	AB	AA	AB	AA	AB	AA	AB	AB	AA	-	AA	AA
AB	AB	AB	AB	AB	AA	AB	AB	AB	AB	AB	AA	AB	AB	AA	AB	AB	AB	AB	AB	AB	AB	AB
AB	AB	AB	AB	AA	AB	AB	AB	AA	AB	AA	AB	AA	AA	AA	AA	AB	AB	AB	AA	AA	AA	AA
AB	-	AB	-	AB	AA	AA	AB	AA	-	-	AB	-	AB	-	-	-	AA	-	-	-	AB	AB
AB	AB	AB	AB	AB	AB	AB	AB	AA	AB	AB	AB	AB	AA	AA	AA	AB	AB	AB	AB	AA	AB	AB
AA	AA	AB	AA	AA	AA	AA	AA	AB	AA	AA	AA	AA	AA	AB	AA	AA	AA	AA	AA	AB	AA	AA
AB	AA	AA	AB	AA	AA	AA	AB	AA	AB	AB	AB	AA	AB	AA	AB	AB	AB	AB	AB	AA	AA	AA
AA	AB	AB	AA	AB	AB	AB	AB	AB	AA	AA	AA	AB	AA	AB	AA	AA	AA	AA	AA	AB	AB	AB
AA	AB	AB	AA	AB	AB	AB	AB	AB	AA	AA	AA	AB	AB	AB	AB	AA	AA	AA	AA	AB	AB	AB
AA	AB	AB	AB	AB	AB	AB	AB	AB	-	-	AB	-	AA	AA	AA	AB	AB	AB	AA	AB	AB	AB
AA	AA	-	AA	AB	-	AB	AA	AB	-	-	AA	-	AB	AB	AB	AB	AA	AA	-	-	AB	AB
AA	AB	AA	AB	AA	AA	AA	AB	AA	AB	AB	AB	AA	AB	AA	AB	AA	AB	AB	AA	AA	AA	AA
AB	AB	AB	AB	AA	AB	AB	AB	AB	AA	AB	AA	AA	AA	AA	AA	AA	AB	AB	AA	AA	AA	AA
AA	-	AB	AB	AB	AA	AA	AB	AA	AA	-	AA	AB	AB	AB	AB	AB	AB	-	AB	-	AB	AB
AA	-	AB	AA	AA	AA	AA	AA	AA	-	AA	AB	-	AB	AB	AB	AA	AA	AA	AA	AA	AA	AA
AA	AB	-	AA	AB	AB	AB	AA	AB	AA	-	AA	AB	AA	AA	AA	AA	AA	AB	-	AB	AB	AB
AB	AB	AB	AB	AB	AB	AB	AB	AB	AB	AB	AB	AB	AB	AA	AB	AB	AB	AB	AB	AB	AB	AB
AB	AB	AA	AB	AB	AB	AB	AB	AB	AB	AB	AB	AB	AA	AA	AA	AB	AB	AB	AB	AB	AB	AB
AB	AB	AB	AB	AB	AB	AB	AB	AB	AB	AB	AB	AB	AB	AB	AB	AB	AB	AB	AB	AB	AB	AB
AA	AA	AB	AA	AA	AA	AA	AA	AA	AA	AA	AA	AA	AB	AA	AB	AA	AA	AA	AA	AA	AA	AB
AB	AB	-	AA	AB	AB	AB	AB	AB	-	-	AA	-	AA	AB	AA	AA	AB	AA	-	-	AB	AB
AA	AB	AB	AA	AB	AB	AB	AA	AB	AA	AA	AA	AB	AA	AB	AA	AA	AA	AA	AA	AB	AB	AB
AA	AB	AA	AB	AA	AA	AA	AA	AA	AB	AB	AB	AA	AB	AA	AB	AA	AB	AB	AA	AB	AB	AA
AA	AA	AA	AA	AB	AA	AA	AA	AA	AB	AA	AA	AB	AA	AB	AA	AB	AA	AA	AB	AB	AB	AB
AA	-	AB	AA	AA	AB	AB	AA	AB	AA	AA	AB	AA	AB	AB	AA	AA	AA	AA	AA	AA	AA	AA
AB	AA	AB	AB	AB	AA	AB	AB	AB	AA	AA	AA	AA	AB	AB	AB	AA	AA	AA	AA	AA	AB	AB
AB	AA	AA	AB	AA	AA	AA	AA	AA	AB	AB	AB	AA	AB	AB	AA	AB	AB	AB	AB	AA	AA	AB
AB	AA	AB	AA	AB	AB	AB	AB	AB	AB	AB	AB	AB	AB	AB	AB	AB	AA	AA	AA	AA	AB	AB
AA	AA	AB	AA	AB	AA	AA	AB	AB	AB	AB	AB	AB	AB	AB	AA	AB	AA	AB	AB	AA	AB	AB
AA	AA	AA	AA	AA	AA	AA	AA	AA	AA	AB	AA	AA	AB	AA	AA	AA	AA	AA	AB	AA	AA	AA
AA	AA	AA	AA	AA	AA	AA	AA	AA	AA	AA	AA	AA	AB	AA	AB	AA	AA	AA	AA	AA	AA	AA
AA	AB	AB	AB	AB	AA	AB	AB	AB	AA	AB	AB	AB	AB	AB	AB	AB	AB	AB	AB	AA	AB	AB
AA	AA	AB	AA	AB	AB	AA	AA	AB	AA	AB	AB	AB	AB	AB	AB	AB	AA	AA	-	AB	AB	AB
AB	AB	AB	AB	AB	AB	AB	AB	AB	AB	AB	AB	AB	AB	AB	AB	AB	AB	AB	AB	AB	AB	AB
AB	AB	AB	AB	AB	AB	AB	AB	AB	AB	AB	AB	AB	AB	AA	AB	AB	AB	AB	AB	AB	AB	AB

snp_B08047	snp_B08147	snp_B08557	snp_B08567	snp_B08897	snp_B09017	snp_B09087	snp_B09207	snp_B09387	snp_B09467	snp_B09587	snp_B09687	snp_B09917	snp_B10447	snp_B10477	snp_B10557	snp_B10577	snp_B10607	snp_B10697	snp_B10767	snp_B10857	snp_B10947	snp_B10977	snp_B11087	snp_B11297	snp_B11337	snp_B11367	snp_B11507	snp_B11537	snp_B11677	snp_B12007	snp_B12287	snp_B12317	snp_B12447	snp_B12537	snp_B12737	snp_B13087	snp_B13167	snp_B13327	snp_B13747	snp_B13827	snp_B13907	snp_B14077	snp_B14167	snp_B14197	snp_B14287	snp_B14327	snp_B14527	snp_B14557	snp_B14577	snp_B14907	snp_B15237	snp_B15287	snp_B15427	snp_B15477	snp_B15487	snp_B15537	snp_B15647	snp_B15677	snp_B15967	snp_B15987	snp_B16037	snp_B16297	snp_B16347	snp_B16507	snp_B16527	snp_B16647	snp_B16657	snp_B16917	snp_B17047	snp_B17167	snp_B17207	snp_B17227	snp_B17407	snp_B17547	snp_B17727	snp_B17817
--	--	--	--	--	--	--	--	--	--	--	--	--	--	--	--	--	--	--	--	--	--	--	--	--	--	--	--	--	LG5	--	LG5	--	LG5	--	--	LG5	--	--	--	--	LG5	LG5	--	--	--	--	--	--	--	--	LG5	--	--	--	--	--	--	LG5	--	--	--	--	--	--	--	--	--	--	--	--	--	--	--	--	--	--
AA	AA	AB	AA	AA	AB	AA	AA	AB	AA	AA	AB	AB	AA	AA	AA	AA	AB	AA	AB	AA	AB	AB	AA	AA	AA	AB	AA	AA	AB	AA	AB	AB	AA	AA	AB	AA	AB	AB	AB	AB	AA	AB	AB	AB	AA	AA	AB	AB	AB	AB	AB	AB	AB	AB	AB	AA	AB	AA	AA	AB	AB	AB	AA	AA	AA	AB	AA	AB	AA	AB	AB	AA	AB	AB	AA	AB
AB	AB	AB	AB	AB	AB	AB	AB	AA	AB	AB	AA	AB	AA	AB	AA	AB	AB	AA	AB	AB	AA	AA	AA	AB	AB	AA	AB	AA	AB	AA	AB	AB	AB	AA	AB	AA	AB	AB	AB	AB	AA	AB	AA	AB	AB	AA	AB	AA	AA	AB	AB	AB	AB	AB	AB	AA	AB	AB	AB	AA	AA	AB	AB	AB	AB	AB	AB	AB	AB	AB	AA	AB	AB	AB	AB	AB
AA	AB	AA	AB	AB	AA	AB	AB	AB	AB	AA	AB	AA	AB	AB	AA	AB	AA	AB	AA	AB	AA	AB	AB	AA	AB	AA	AB	AB	AB	AB	AB	AA	AB	AB	AA	AA	AA	AB	AA	AA	AB	AB	AB	AB	AB	AB	AB	AA	AA	AA	AB	AB	AA	AA	AB	AB	AA	AB	AB	AA	AA	AB	AA	AB	AB	AB	AB	AA	AB	AA	AB	AB	AB	AB	AA	AB
AA	AB	AB	AB	AB	AB	AA	AA	AB	-	AB	AA	AB	AB	AB	AA	AB	AB	AA	AB	AB	AA	AA	AA	-	AA	AB	AB	-	-	AA	AB	AB	AB	AB	AB	AA	AA	AB	AA	AB	AA	AB	AB	AB	AA	AB	AA	AA	AA	AB	AB	AB	AB	AB	AB	AA	AB	AB	AB	AA	AA	AB	AA	AB	AB	AB	AB	AA	AA	AB	AB	AB	-	AA	AB	AA
AB	AB	AB	AB	AB	AB	AB	AA	AA	AB	AA	AA	AB	AA	AB	AB	AA	AB	AB	AA	AB	AA	AA	AA	AB	AB	AB	AB	AB	AA	AB	AB	AB	AB	AA	AA	AB	AA	AA	AB	AB	AB	AB	AA	AA	AB	AB	AB	AB	AB	AA	AA	AB	AA	AB	AA	AB	AA	AB	AB	AA	AB	AA	AB	AA	AA	AB	AB	AB	AB	AB	AA	AA	AA	AA	AB	AA
-	AA	AA	AA	AB	AA	AA	-	AB	-	AA	AB	AA	-	AA	AB	AB	AB	AA	AA	AA	AA	-	AB	-	AA	AB	AA	-	-	AA	-	AB	AA	AA	-	AB	-	-	-	AA	-	AA	AB	-	AA	AB	AB	AA	AA	AB	-	AB	AB	AA	AB	AA	AA	AA	-	AB	-	AB	AA	AB	-	AB	AA	-	AB	AA	AB	AA	AB	-	AA	-
AA	-	AA	AA	AA	AA	AA	AA	AA	AA	AA	AA	AA	AB	AA	AA	AB	AA	AA	AA	AA	AA	AA	AA	AA	AA	AA	AA	AA	AA	AA	AA	AB	AB	AB	AA	AA	AA	AA	AA	AA	AA	AA	AB	AA	AA	AA	AA	AA	AA	AB	AA	AA	AA	AA	AB	AB	AB	AA	AA	AA	AB	AA	AA	AA	AB	AA	AA	AA	AA	AB	AB	AB	AA	AA	AA	AA
AB	AB	AB	AB	AB	AB	AB	AB	AA	AB	AB	AA	AB	AA	AB	AB	AB	AB	AB	AB	AA	AB	AB	AA	AB	AB	AB	AB	AB	AB	AB	AB	AB	AB	AB	AA	AA	AB	AB	AB	AB	AB	AB	AA	AB	AB	AA	AA	AB	AB	AB	AB	AB	AB	AB	AB	AB	AB	AB	AB	AB	AA	AB	AB	AB	AB	AB	AB	AB	AB	AB	AA	AB	AA	AB	AB	AB	AA
AB	AB	AA	AB	AB	AB	AA	AA	AB	AB	AA	AB	AA	AB	AB	AB	AB	AB	AB	AB	AA	AB	AB	AA	AB	AA	AB	AB	AB	AB	AB	AB	AA	AB	AB	AA	AA	AB	AA	AB	AB	AA	AA	AB	AB	AA	AB	AA	AA	AA	AA	AB	AB	AA	AA	AB	AB	AB	AA	AB	AB	AB	AA	AA	AA	AA	AB	AA	AA	AB	AA	AB	AB	AB	AB	AB	AB
AB	AA	AA	AB	AB	AA	AA	AA	AA	AB	AB	AA	AA	AB	AA	AB	AB	AA	AB	AA	AA	AB	AB	AA	AB	AB	AA	AA	AB	AB	AA	AB	AA	AA	AA	AB	AA	AB	AA	AA	AA	AA	AA	AB	AB	AB	AA	AA	AA	AA	AB	AA	AB	AA	AB	AA	AB	AB	AB	AB	AA	AB	AA	AB	AB	AB	AA	AB	AA	AB	AB	AB	AB	AB	AA	AB	AB

snp_B1786	snp_B1791	snp_B1793	snp_B1838	snp_B1840	snp_B1866	snp_B1880	snp_B1913	snp_B1933	snp_B1943	snp_B1987	snp_B1989	snp_B1994	snp_B2050	snp_B2058	snp_B2072	snp_B2095
7	7	7	7	7	7	7	7	7	7	7	7	7	7	7	7	7
--	--	--	--	--	--	LG5	--	--	--	--	LG5	--	LG5	--	--	--
AB	AB	AB	AB	AA	AA	AA	AB	AA	AA	AA	AA	AB	AB	AB	AA	AA
AB	AB	AB	AA	AA	AB	AB	AB	AB	AB	AA	AB	AB	AB	AB	AB	AB
AA	AA	AA	AA	AB	AB	AB	AA	AB	AB	AB	AB	AB	AA	AA	AB	AB
-	AB	AB	-	AA	AB	AB	-	-	AB	-	AB	AB	-	AB	AB	AB
AB	AA	AA	AB	AB	AA	AB	AA	AB	AA	AA	AB	AA	AB	AA	AA	AA
-	AB	AB	-	AA	AA	AA	-	-	-	-	AA	AB	AA	AB	AA	AB
AA	AB	AB	AA	AA	AA	AA	AA	AB	AA	AB	AA	AB	AA	AA	AA	AB
AB	AB	AB	AB	AB	AB	AB	AB	AB	AB	AA	AB	AB	AB	AB	AB	AB
-	AA	AB	AA	AA	AB	AB	AA	AB	-	AA	AB	AB	AA	AA	AB	AA
AA	AB	AB	AA	AA	AA	AA	AA	AB	AB	AA	AA	AA	AA	AA	AB	AA
AB	AA	AB	AA	AB	AA	AB	AB	AA	AA	AB	AA	AB	AA	AA	AB	AB
AB	AB	AB	AB	AB	AB	AB	AB	AB	AA	AA	AB	AB	AB	AB	AB	AB
AA	AB	AB	AA	AA	AB	AB	AB	AB	AB	AA	AA	AB	AA	AA	AB	AB
AA	AB	AB	AA	AA	AA	AA	AB	AA	AA	AB	AA	AA	AA	AA	AB	AA
AA	AB	AA	AA	AA	AA	AA	AB	AB	AB	AA	AB	AA	AB	AB	AA	AA
AB	AB	AB	AB	AB	AB	AB	AA	AB	AB	AB	AB	AB	AB	AB	AB	AB
AA	AB	AB	AA	AA	AA	AA	AB	AA	AB	AA	AA	AB	AA	AB	AA	AA
-	AA	AB	-	AB	AB	-	AA	-	-	-	AB	AB	AB	-	AB	AA
AB	AB	AB	AB	AB	AB	AB	AB	AB	AB	AB	AB	AB	AB	AB	AB	AB
AA	AA	AA	AA	AA	AA	AA	AA	AB	AA	AB	AA	AA	AA	-	-	AA
AA	AB	AB	AA	AA	AA	AA	AB	AB	AB	AB	AA	AB	AA	AB	AB	AB
AA	AA	AA	AA	AA	AA	AA	AA	AB	AA	AB	AA	AA	AA	AA	AA	AA
-	AA	AA	-	-	AB	AB	-	-	-	-	AB	AB	-	-	-	-
AA	AB	AB	AA	AA	AA	AA	AB	AB	AA	AB	AA	AB	AA	AB	AA	AB
AB	AB	AB	AB	AB	AB	AB	AB	AB	AB	AA	AB	AB	AB	AB	AB	AB
AB	AA	AA	AB	AA	AA	AA	AB	AA	AA	AB	AA	AA	AB	AB	AA	AA
AA	AA	AA	AA	AA	AA	AA	AA	AA	AA	AB	AA	AA	AA	AA	AA	AA
AA	AB	AB	AA	AA	AB	AB	AA	AB	AB	AA	AB	AA	AB	AA	AB	AA
AB	AB	AA	AB	AA	AB	AA	AB	-	AA	AB	AA	AB	AB	AA	AB	AB
-	AA	-	-	AB	AB	AB	-	-	-	-	AB	AA	-	-	AB	-
AA	AB	AB	AA	AA	AA	AA	AB	AB	AA	AB	AA	AB	AA	AB	AA	AB
AB	AA	AA	AB	AB	AB	AB	AA	AA	AB	AA	AB	AA	AB	AA	AB	AA
-	AA	AA	AB	AB	AB	AB	AA	AB	-	-	AB	AA	-	-	-	-
AB	AB	AB	-	AA	AA	AA	-	-	AA	-	AB	-	AB	AA	AB	AB
-	AA	AA	AA	AB	AA	AA	AA	AA	-	AB	AA	AA	AB	AB	AA	AA
-	AB	AB	AB	AB	AB	AB	AB	AB	AB	AB	AB	AB	AB	AB	AB	AB
AA	AB	AB	AA	AB	AA	AB	AA	AB	AB	AB	AA	AB	AA	AA	AB	AB
AA	AB	AB	AA	AA	AA	AA	AB	AA	AA	AA	AA	AB	AA	AB	AA	AB
AA	AA	AB	AA	AA	AB	AB	AB	AA	AB	AB	AB	AB	AA	AA	AB	AA
-	AA	AA	AB	AB	AA	AB	-	AA	AA	AA	AB	AA	-	AA	AA	AA
AB	AB	AB	AB	AB	AB	AB	AB	AB	AB	AA	AB	AB	AB	AB	AB	AB
AB	AA	AA	AB	AB	AB	AA	AA	AA	AB	AA	AB	AA	AB	AB	AA	AA
AB	AA	AA	AB	AB	AB	AB	AB	AB	AA	AB	AB	AA	AB	AB	AB	AA
AB	AA	AA	AA	AB	AB	AB	AA	AA	AB	-	AB	AB	AB	-	-	AA
AB	AB	AB	AB	AA	AA	AA	AA	AA	AA	AB	AA	AA	AB	AB	AB	AA

snp_B3174	snp_B3177	snp_B3205	snp_B3210	snp_B3219	snp_B3229	snp_B3234	snp_B3260	snp_B3284	snp_B3342	snp_B3362	snp_B3405	snp_B3435	snp_B3444	snp_B3446	snp_B3472	snp_B3515	snp_B3543	snp_B3544	snp_B3589	snp_B3591	snp_B3599	snp_B3611
7	7	7	7	7	7	7	7	7	7	7	7	7	7	7	7	7	7	7	7	7	7	7
--	--	--	--	--	--	--	--	--	--	--	--	--	--	--	--	--	LG5	--	--	LG5	--	--
AA	AB	AB	AA	AB	AA	AB	AA	AB	AA	AA	AB	AB	AB	AA	AB	AA	AA	AB	AB	AA	AB	AB
AA	AB	AB	AA	AB	AA	AB	AB	AB	AA	AA	AB	AB	AB	AA	AA	AB	AA	AB	AB	AA	AB	AB
AB	AB	AB	AA	AA	AB	AB	AB	AB	AB	AA	AB	AB	AA	AB	AB	AB	AB	AA	AA	AB	AB	AB
AA	AA	AA	AA	AB	AA	AB	AB	AA	AA	AA	AB	AB	AB	AA	AB	AB	AB	AB	AB	AB	AB	AB
AA	AA	AA	AB	AA	AB	AB	AA	AB	AB	AA	AB	AA	AB	AB	AA	AA	AB	AB	AB	AB	AB	AB
AA	AB	AA	AA	AB	AA	AA	-	AA	AA	AB	AA	AA	-	-	-	AB	AA	-	AA	AA	AB	AB
AB	AA	AA	AB	AB	AA	AB	AA	AA	AA	AA	AA	AA	AA	AA	AB	AB	AA	AA	AA	AA	AA	AB
AA	AB	AB	AA	AB	AB	AB	AB	AB	AB	AB	AA	AB	AB	AB	AA	AB	AB	AB	AB	AB	AA	AB
AA	AB	AA	AA	AA	AB	AB	AB	AB	AA	AB	AA	AB	AB	AA	AA	AA	AB	AA	AB	AB	AA	AB
AA	AA	AA	AB	AB	AA	AA	AB	AB	AA	AB	AA	AB	AA	AA	AB	AB	AB	AA	AB	AA	AA	AB
AA	AA	AA	AA	AB	AB	AB	AA	AA	AA	AA	AB	AA	AB	AB	AA	AA	AB	AA	AA	AB	AA	AA
AA	AB	AB	AA	AB	AB	AB	AB	AB	AB	AA	AB	AA	AB	AB	AA	AB	AB	AB	AB	AB	AB	AB
AA	AB	AB	AA	AB	AA	AB	AB	AB	AA	AB	AA	AB	AB	AA	AA	AB	AB	AB	AB	AB	AB	AA
AB	AB	AA	AB	AB	AA	AA	AA	AA	AA	AA	AA	AA	AA	AB	AA	AB	AA	AB	AA	AA	AA	AB
AB	AB	AA	AB	AB	AA	AA	AA	AA	AA	AA	AB	AA	AB	AB	AA	AA	AA	AB	AB	AA	AA	AA
AB	AB	AA	AB	AB	AB	AB	AB	AA	AB	AB	AB	AB	AB	AB	AB	AB	AB	AB	AB	AB	AB	AB
AA	AA	AB	AA	AB	AA	AB	AB	AA	AA	AB	AA	AB	AA	AA	AA	AA	AB	AA	AB	AB	AB	AB
-	AA	-	AB	-	AB	-	-	AB	AB	AB	AB	AA	AA	-	-	AA	AB	-	AA	AB	AB	AB
AB	AB	AB	AB	AB	AB	AB	AB	AB	AB	AB	AB	AA	AB	AB	AB	AB	AB	AB	AB	AB	AB	AB
AB	AB	AB	AB	AA	AA	AA	AA	AB	AB	AA	AA	AA	AA	AA	AB	AB	AA	AA	AA	AA	AA	AB
AA	AB	AB	AB	AB	AA	AA	AB	AB	AA	AB	AA	AB	AB	AA	AB	AB	AB	AB	AB	AA	AB	AA
AA	AA	AB	AA	AA	AA	AA	AA	AA	AA	AB	AA	AB	AA	AA	AB	AB	AA	AA	AA	AA	AA	AB
AB	AA	-	AB	-	AB	-	AB	AA	AB	AB	AB	AB	AA	-	AA	AA	AB	-	-	AA	AA	-
AA	AB	AA	AA	AB	AA	AA	AA	AA	AA	AB	AA	AA	AB	AA	AB	AB	AA	AB	AA	AA	AB	AB
AB	AB	AA	AB	AA	AB	AB	AB	AB	AB	AA	AB	AB	AB	AB	AB	AB	AB	AB	AB	AB	AB	AB
AA	AA	AA	AB	AA	AA	AA	AA	AB	AA	AA	AB	AB	AA	AA	AB	AB	AA	AA	AB	AA	AB	AB
AA	AA	AA	AB	AA	AA	AA	AA	AA	AA	AA	AA	AA	AA	AA	AB	AA	AA	AA	AA	AA	AA	AB
AA	AB	AB	AB	AB	AA	AB	AB	AA	AA	AB	AA	AA	AA	AA	AB	AA	AA	AA	AB	AB	AA	AA
AB	AB	AB	AB	AB	AA	AA	AA	AA	AA	AB	AA	AB	AA	AA	AB	AA	AB	AA	AB	AA	AA	AB
-	AB	AB	AB	AA	AB	AB	AB	AB	AB	-	AB	AA	AA	-	AA	AA	AB	-	AA	AB	AB	AA
AB	AB	AA	AB	AB	AA	AB	AA	AB	AB	AA	AA	AA	AA	AA	AB	AB	AA	AA	AA	AA	AB	AB
AA	AB	AA	AB	AA	AB	AB	AB	AB	AB	AB	AB	AB	AB	AB	AA	AA	AB	AB	AB	AB	AA	AB
AA	AB	AA	AB	AA	AB	AB	AB	AB	AB	AA	AB	AB	AA	AB	-	AA	AB	AA	AB	AB	AA	AA

snp_B3616	snp_B3682	snp_B3683	snp_B3687	snp_B3711	snp_B3723	snp_B3724	snp_B3730	snp_B3737	snp_B3749	snp_B3750	snp_B3763	snp_B3791	snp_B3798	snp_B3802	snp_B3847	snp_B3878	snp_B3897	snp_B3902	snp_B3903	snp_B3945	snp_B3947	snp_B3961	snp_B3963	snp_B3964	snp_B3991
7	7	7	7	7	7	7	7	7	7	7	7	7	7	7	7	7	7	7	7	7	7	7	7	7	7
--	LG5	--	--	LG5	--	--	--	--	--	--	LG5	--	LG5	--	--	--	--	--	--	--	--	--	--	LG5	--

snp_B4008	snp_B4020	snp_B4028	snp_B4041	snp_B4096	snp_B4141	snp_B4149	snp_B4168	snp_B4184	snp_B4195	snp_B4204	snp_B4210	snp_B4223	snp_B4226	snp_B4229	snp_B4256	snp_B4259	snp_B4267	snp_B4271	snp_B4277	snp_B4292	snp_B4306	snp_B4307	snp_B4330	snp_B4332	snp_B4349	snp_B4366	snp_B4377
7	7	7	7	7	7	7	7	7	7	7	7	7	7	7	7	7	7	7	7	7	7	7	7	7	7	7	7
--	--	--	--	--	--	--	--	--	--	LG5	--	--	LG5	--	--	--	--	--	LG5	LG5	--	--	--	--	--	--	--

snp_B4382	snp_B4407	snp_B4424	snp_B4444	snp_B4468	snp_B4470	snp_B4473	snp_B4487	snp_B4495	snp_B4499	snp_B4543	snp_B4546	snp_B4581	snp_B4592	snp_B4620	snp_B4621	snp_B4638	snp_B4650	snp_B4651	snp_B4674	snp_B4677	snp_B4679	snp_B4724	snp_B4764	snp_B4781	snp_B4821	snp_B4859	snp_B4865	snp_B4875	snp_B4919	snp_B4939	snp_B4970	snp_B4985	snp_B5049	snp_B5056	snp_B5065	snp_B5080	snp_B5109	snp_B5122	snp_B5126	snp_B5161	snp_B5189	snp_B5191	snp_B5201	snp_B5207	snp_B5211	snp_B5227	snp_B5228	snp_B5232	snp_B5291	snp_B5332	snp_B5335	snp_B5375	snp_B5420	snp_B5432	snp_B5472	snp_B5546	snp_B5550	snp_B5554	snp_B5561	snp_B5567	snp_B5593	snp_B5646	snp_B5657	snp_B5667	snp_B5685	snp_B5689	snp_B5691	snp_B5712	snp_B5717	snp_B5754	snp_B5772	snp_B5814	snp_B5839	snp_B5846	snp_B5850	snp_B5859
7	7	7	7	7	7	7	7	7	7	7	7	7	7	7	7	7	7	7	7	7	7	7	7	7	7	7	7	7	7	7	7	7	7	7	7	7	7	7	7	7	7	7	7	7	7	7	7	7	7	7	7	7	7	7	7	7	7	7	7	7	7	7	7	7	7	7	7	7	7	7	7	7	7	7	7	7
--	--	--	--	--	--	--	--	--	--	--	--	--	--	--	--	--	--	--	--	--	--	--	--	LG5	--	--	--	--	--	--	--	--	LG5	--	--	--	--	--	--	--	--	--	--	LG5	--	--	--	--	--	--	--	--	LG5	LG5	--	LG5	--	--	--	--	--	--	--	--	--	--	--	--	--	--	--	LG5	--	LG5	--	--
AB	AA	AB	AA	AA	AB	AB	AA	AA	AB	AA	AB	AB	AA	AB	AB	AA	AA	AB	AB	AA	AB	AA	AB	AA	AB	AB	AA	AA	AB	AA	AA	AB	AB	AB	AB	AA	AA	AB	AB	AB	AA	AA	AA	AB	AA	AB	AA	AA	AA	AB	AA	AA	AB	AA	AA	AA	AA	AB	AA	AB	AB	AA	AB	AA	AB	AA	AA	AB	AA	AB	AA	AA	AB	AA	AA	AA

snp_B5877	snp_B5887	snp_B5908	snp_B5939	snp_B5951	snp_B5956	snp_B5964	snp_B5997	snp_B6032	snp_B6163	snp_B6293	snp_B6305	snp_B6325	snp_B0007	snp_B0008	snp_B0009	snp_B0012	snp_B0017	snp_B0018	snp_B0058
7	7	7	7	7	7	7	7	7	7	7	7	7	8	8	8	8	8	8	8
--	--	--	--	--	--	LG5	--	--	--	--	--	--	--	--	--	--	--	--	--
AA	AB	AB	AB	AA	AB	AA	AB	AB	AB	AB	AA	AB	AB	AA	AB	AA	AB	AA	AA
AB	AB	AB	AB	AA	AB	AB	AB	AA	AA	AA	AB	AB	AB	AB	AB	AA	AA	AA	AA
AB	AB	AB	AA	AA	AB	AB	AA	AB	AB	AB	AA	AA	AA	AA	AB	AA	AB	AA	AA
AA	AB	AA	AA	AA	AB	AB	AB	AB	AA	AB	AB	-	AB	AB	AB	AA	-	AB	AB
AB	AA	AA	AA	AB	AB	AB	AB	AA	AA	AA	AB	AB	AB	AA	AB	AA	AB	AB	AB
AB	AB	AA	AB	AB	AB	AA	AB	-	AB	-	-	-	AB	AB	AA	-	-	AA	-
AB	AB	AA	AA	AA	AA	AA	AA	AB	AB	AB	AA	AA	AA	AB	AA	AB	AA	AA	AA
AB	AB	AB	AA	AA	AB	AB	AB	AA	AA	AB	AB	AB	AA	AA	AB	AA	AA	AA	AA
AB	AA	AB	AB	AA	AA	AB	AB	AB	AB	AB	AB	AA	AA	AA	AB	AA	AA	AA	AA
AB	AB	AB	AB	AA	AA	AA	AA	AA	AB	AB	AB	AA	AA	AA	AA	AA	AB	AB	AB
AA	AA	AB	AA	AA	AB	AB	AA	AA	AA	AA	AA	AA	AB	AB	AB	AA	AA	AB	AB
AA	AA	AB	AB	AA	AB	AB	AB	AA	AA	AA	AA	AB	AA	AA	AB	AA	AA	AA	AA
AB	AB	AA	AB	AA	AA	AB	AB	AA	AA	AA	AB	AB	AA	AA	AB	AA	AB	AB	AB
AB	AB	AA	AB	AA	AB	AA	AB	AB	AB	AB	AB	AB	AB	AB	AA	AB	AB	AA	AA
AA	AA	AB	AB	AA	AA	AB	AB	AA	AA	AB	AA	AB	AB	AB	AB	AA	AB	AB	AB
AB	AB	AB	AB	AB	AB	AB	AB	AB	AB	AB	AB	AB	AB	AB	AB	AA	AB	AB	AB
AA	AA	AB	AB	AB	AB	AB	AB	AA	AA	AA	AB	AA	AB	AB	AB	AB	AB	AB	AB
AB	AA	AB	AA	AA	AA	AA	AA	-	AB	-	-	-	AB	AA	AB	AB	-	AA	-
AB	AB	AB	AB	AB	AB	AA	AB	AB	AB	AB	AB	AB	AB	AB	AA	AA	AA	AA	AA
AB	AB	AA	AB	AA	AA	AA	AB	AB	AB	AB	AA	AA	AB	AB	AB	AB	AB	AB	AB
AB	AB	AA	AB	AB	AB	AA	AB	AB	AB	AB	AB	AB	AB	AB	AB	AB	AB	AB	AA
AB	AB	AA	AA	AB	AA	AA	AA	AB	AB	AB	AA	AA	AB	AA	AA	AA	AA	AA	AA
AB	AA	AB	AA	AB	AB	AB	AA	-	AA	-	-	-	AA	AB	AA	-	-	AA	-
AB	AB	AA	AB	AB	AB	AA	AB	AB	AB	AB	AA	AB	AA	AA	AB	AB	AB	AB	AB
AB	AB	AB	AB	AA	AB	AB	AB	AA	AB	AA	AB	AB	AA	AA	AB	AB	AB	AB	AB
AA	AB	AB	AB	AA	AA	AA	AA	AB	AB	AB	AA	AA	AA	AA	AA	AA	AB	AA	AA
AA	AA	AB	AA	AA	AA	AA	AA	AB	AB	AB	AA	AA	AB	AA	AB	AA	AA	AB	AA
AB	AA	AB	AA	AB	AB	AB	AA	AA	AA	AA	AA	AA	AA	AB	AB	AB	AB	AA	AB
AB	AB	AA	AA	AB	AA	AB	AA	AB	AB	AB	AA	AA	AB	AB	AB	AB	-	AB	AB
-	AA	AB	AA	AB	-	-	AA	-	AB	-	-	-	AA	AA	AB	AA	-	AA	-
AB	AB	AB	AB	AA	AB	AA	AB	AB	AB	AB	AA	AA	AB	AB	AB	AB	AB	AB	AB
AA	AA	AB	AA	AB	AA	AB	AA	AA	AB	AA	AB	AB	AA	AA	AB	AB	AA	AA	AA
AA	AA	AB	AA	AA	AA	-	AA	AA	AA	AA	-	-	AB	AB	AA	AA	-	AA	AA
AB	AB	AB	AB	AA	AB	-	AB	AB	AB	AB	-	-	AB	AB	AB	AB	AB	AB	-
AA	AB	AB	AB	AA	AA	AA	AA	-	AB	-	-	-	AA	AA	AB	AA	-	AA	AA
AB	AB	AB	AB	AB	AB	AB	AB	AB	AB	AB	-	AB	AB	AB	AB	AB	AB	AB	AB
AB	AB	AA	AA	AB	AA	AB	AA	AB	AB	AB	AB	AA	AA	AA	AA	AA	AA	AA	AA
AA	AA	AB	AB	AA	AA	AB	AB	AA	AA	AA	AA	AA	AB	AB	AB	AA	AB	AB	AB
AB	AA	AB	AA	AB	AA	AB	AA	AA	AB	AA	AB	AA	AB	AB	AB	AB	AB	AB	AB
AB	AA	AA	AA	AB	-	AB	AB	AA	AA	-	-	-	AA	AB	AB	AB	AA	AA	AA
AA	AB	AB	AB	AA	AB	AB	AB	AA	AA	AA	AB	AB	AB	AA	AA	AA	AB	AB	AB
AA	AA	AB	AB	AA	AB	AB	AB	AA	AA	AA	AA	AB	AB	AA	AA	AA	AB	AA	AB
AB	AA	AB	AB	AA	AB	AB	AB	AB	AB	AB	AA	AB	AB	AB	AA	AB	AB	AB	AB
AA	AA	AA	AA	AB	AA	AB	AB	AA	AA	AA	AB	AA	AA	AA	AA	AB	AA	AB	AB
AA	AB	AB	AA	AA	AA	AA	AB	AA	AB	AB	AB	AB	AB	AA	AB	AB	AA	AA	AB
AA	AB	AB	AB	AA	AA	AA	AB	AB	AB	AB	AB	AB	AB	AB	AA	AB	AB	AB	AB
AB	AB	AB	AA	AB	AA	AB	AA	AB	AB	AB	AB	AA	AA	AB	AA	AB	AA	AB	AA
AA	AA	AB	AA	AA	AA	AB	AA	AA	AB	AA	AB	AA	AA	AA	AA	AA	AB	AB	AB
AB	AB	AA	AB	AB	AA	AB	AB	AB	AB	AB	AB	AB	AA	AB	AA	AA	AB	AB	AB
AB	AA	AA	AB	AB	AA	AB	AB	AA	AA	AA	AB	AB	AB	AB	AA	AA	AB	AB	AB
AB	AB	AB	AB	AA	AB	AB	AB	AA	AA	AA	AB	AB	AB	AB	AA	AB	AB	AB	AB
AA	AA	AA	AA	AB	AA	AB	AB	AA	AA	AA	-	-	AA	AB	AA	AA	-	AA	-
AA	AA	AB	AB	AB	AB	AB	AB	AA	AB	AA	AB	AA	AB	AB	AB	AB	AA	AA	AA

snp_B10728	snp_B10738	snp_B10938	snp_B10998	snp_B11048	snp_B11078	snp_B11448	snp_B11458	snp_B11608	snp_B11878	snp_B11908	snp_B12138	snp_B12328	snp_B12468	snp_B12528	snp_B12578	snp_B12598	snp_B12628	snp_B12648	snp_B13128	snp_B13158	snp_B13698	snp_B13788	snp_B13968	snp_B14058	snp_B14308	snp_B14368	snp_B14378	snp_B14388	snp_B14408	snp_B14468	snp_B14648	snp_B14708	snp_B14878	snp_B15018	snp_B15298	snp_B15328	snp_B15458	snp_B15578	snp_B15698	snp_B15738	snp_B15898	snp_B15938	snp_B15998	snp_B16138	snp_B16208	snp_B16328	snp_B16468	snp_B17068	snp_B17758	snp_B17828	snp_B18008	snp_B18068	snp_B18178	snp_B18308	snp_B18418	snp_B18478	snp_B18738	snp_B18868	snp_B19568	snp_B19888	snp_B20078	snp_B20388	snp_B20418	snp_B20548	snp_B20708	snp_B20828	snp_B20928	snp_B21178	snp_B21418	snp_B21838	snp_B21858	snp_B21868	snp_B21968	snp_B22148	snp_B22188	snp_B22348
--	--	--	--	--	--	--	--	--	--	--	--	--	--	--	--	--	--	--	--	--	--	--	--	--	--	--	--	--	LG10	--	--	--	--	--	--	--	--	--	--	--	--	--	--	--	--	--	--	--	--	--	--	--	--	--	--	--	--	--	--	--	--	LG10	LG10	--	--	--	--	LG10	--	--	--	--	--	--	--	--
AA	AA	AB	AA	AB	AB	AA	AA	AB	AB	AA	AA	AA	AB	AA	AB	AB	AB	AA	AA	AA	AB	AB	AA	AB	AA	AB	AB	AA	AA	AA	AB	AA	AA	AA	AB	AA	AA	AB	AB	AB	AA	AA	AA	AA	AA	AA	AB	AB	AA	AA	AB	AB	AA	AB	AB	AA	AA	AB	AA	AB	AB	AB	AB	AB	AA	AB	AA	AB	AB	AA	AA	AB	AB	AA	AA	AA
AA	AA	AB	AB	AB	AB	AA	AA	AA	AB	AA	AA	AA	AB	AA	AB	AA	AB	AA	AA	AA	AB	AB	AB	AB	AB	AB	AB	AA	AB	AB	AA	AA	AB	AA	AB	AA	AA	AB	AA	AB	AB	AB	AA	AA	AA	AB	AB	AB	AA	AA	AB	AB	AB	AB	AA	AB	AB	AB	AA	AB	AB	AB	AB	AA	AB	AB	AA	AB	AA	AA	AA	AB	AB	AA	AA	AA
AA	AA	AB	AA	AA	AB	AA	AA	AA	AB	AB	AB	AA	AB	AB	AB	AA	AB	AA	AA	AA	AB	AB	AB	AB	AB	AA	AB	AA	AB	AA	AB	AA	AA	AA	AA	AA	AA	AB	AA	AB	AB	AB	AB	AA	AB	AB	AA	AA	AA	AA	AA	AA	AB	AB	AB	AB	AB	AB	AA	AB	AA	AB	AB	AA	AB	AB	AA	AB	AA	AB	AA	AB	AA	AA	AA	AA
-	AA	AB	AB	AB	AB	AA	AB	AB	AB	AB	AA	AA	AB	AB	AB	AB	AB	AA	AB	AA	AB	AB	AB	AB	AA	AB	AB	AB	-	AB	-	AB	AB	AB	AB	AB	AB	AB	AA	AB	AB	AA	AA	AB	AA	AB	AB	AB	AB	AA	AB	AB	AB	AB	AA	AB	AB	AB	AA	AA	AA	AB	AB	AA	AB	AB	-	AB	AB	AB	AB	AA	AB	AB	AB	AA
AB	AA	AB	AA	AA	AB	AA	AB	AB	AB	AB	AA	AA	AB	AB	AB	AA	AB	AA	AB	AA	AB	AA	AA	AB	AB	AA	AB	AA	AA	AB	AB	AA	AA	AB	AB	AB	AB	AB	AA	AB	AB	AB	AB	AA	AA	AA	AA	AB	AB	AB	AB	AB	AA	AA	AA	AB	AB	AB	AA	AB	AA	AB	AB	AB	AA	AB	AA	AB	AA	AA	AA	AB	AB	AB	AB	AA
-	-	AA	AA	AB	AB	-	AA	-	AA	AA	AA	AA	AA	AA	AB	AB	AA	-	-	AB	AA	AB	AB	-	-	AA	AA	-	-	AB	-	-	-	AA	AB	-	-	-	-	-	-	AA	-	-	-	AA	AB	AB	AA	-	AA	AB	AA	-	-	AA	-	AB	AA	AB	AB	AB	-	AB	AA	-	-	AB	AB	AA	AA	AB	AB	-	AA	AB
AA	AA	AB	AB	AA	AB	AA	AB	AA	AA	AA	AB	AA	AB	AA	AB	AB	AA	AB	AA	AA	AB	AA	AB	AA	AA	AB	AA	AA	AA	AB	AA	AA	AA	AB	AA	AB	AA	AA	AA	AB	AB	AA	AB	AA	AA	AA	-	AA	AA	AA	AA	AA	AA	AA	AA	AA	AA	AA	AA	AA	AA	AA	AA	AA	AB	AB	AB	AB	AB	AA	AB	AA	AA	AA	AA	AA
AA	AB	AB	AA	AA	AB	AB	AA	AB	AA	AA	AB	AB	AB	AA	AB	AA	AA	AA	AA	AB	AA	AA	AB	AA	AB	AA	AA	AA	AB	AB	AA	AA	AA	AB	AA	AB	AA	AA	AB	AB	AB	AA	AB	AA	AA	AB	AA	AA	AA	AB	AA	AA	AB	AA	AA	AB	AA	AA	AA	AA	AB	AA	AA	AA	AB	AA	AA	AB	AB	AA	AA	AA	AA	AA	AA	AB
AA	AA	AA	AA	AB	AA	AB	AA	AB	AB	AA	AA	AB	AA	AB	AA	AA	AA	AB	AA	AA	AB	AB	AA	AA	AA	AA	AB	AA	AB	AB	AA	AA	AB	AA	AA	AA	AB	AB	AA	AA	AA	AB	AA	AA	AB	AA	AA	AA	AA	AB	AB	AB	AB	AB	AB	AA	AB	AB	AA	AB	AB	AB	AB	AA	AA	AB	AA	AB	AA	AA	AB	AB	AA	AA	AB	AA
AB	AB	AB	AA	AA	AB	AA	AA	AA	AB	AA	AA	AB	AA	AB	AA	AA	AA	AA	AA	AB	AA	AA	AB	AB	AA	AA	AB	AB	AA	AA	AB	AA	AB	AA	AA	AA	AB	AB	AA	AB	AA	AB	AA	AA	AA	AA	AA	AB	AB	AA	AB	AA	AA	AB	AA	AB	AA	AB	AA	AB	AA	AB	AB	AA	AB	AB	AA	AB	AA	AA	AA	AB	AB	AA	AB	AA
AB	AA	AB	AA	AB	AB	AA	AA	AA	AB	AB	AA	AA	AB	AA	AB	AB	AB	AB	AA	AA	AA	AB	AB	AA	AB	AB	AA	AB	AA	AB	AB	AA	AA	AA	AB	AB	AA	AB	AB	AA	AA	AB	AB	AB	AA	AA	AB	AB	AB	AA	AB	AA	AB	AA	AA	AB	AA	AA	AA	AB	AA	AB	AB	AA	AB	AB	AB	AB	AA	AA	AB	AB	AB	AA	AB	AA
AA	AB	AB	AA	AB	AB	AA	AA	AA	AA	AA	AA	AA	AA	AA	AB	AA	AA	AA	AA	AB	AA	AA	AB	AA	AB	AA	AA	AA	AB	AA	AA	AA	AB	AA	AB	AA	AA	AA	AB	AA	AB	AB	AA	AA	AA	AA	AA	AA	AA	AA	AA	AB	AB	AB	AA	AA	AA	AB	AA	AA	AB	AB	AA	AA	AB	AB	AA	AB	AA	AA	AA	AA	AA	AA	AA	AB
AB	AA	AB	AA	AA	AB	AB	AB	AA	AA	AB	AB	AB	AA	AB	AB	AB	AB	AA	AB	AA	AA	AA	AB	AA	AA	AA	AA	AB	AB	AA	AB	AB	AA	AB	AA	AB	AB	AA	AA	AB	AB	AA	AA	AB	AB	AA	AA	AA	AA	AB	AA	AA	AB	AB	AB	AA	AB	AA	AA	AB	AB	AA	AA	AA	AB	AA	AB	AA	AA	AB	AB	AA	AA	AB	AB	AA
AA	AB	AB	AB	AA	AA	AB	AA	AB	AB	AA	AB	AB	AA	AA	AA	AA	AB	AB	AA	AB	AB	AA	AB	AB	AB	AA	AB	AA	AB	AB	AA	AB	AB	AA	AA	AA	AB	AA	AB	AA	AA	AA	AB	AA	AA	AA	AA	AA	AA	AB	AB	AB	AB	AB	AA	AB	AB	AA	AB	AB	AB	AA	AA	AB	AB	AB	AA	AB	AA	AA	AB	AA	AB	AA	AB	AB
AB	AA	AB	AA	AB	AA	AA	AA	AB	AA	AB	AA	AB	AB	AA	AA	AB	AB	AB	AA	AB	AB	AA	AB	AA	AA	AA	AA	AB	AB	AA	AB	AA	AA	AB	AA	AA	AA	AB	AB	AA	AA	AA	AA	AB	AA	AB	AB	AB	AB	AA	AB	AB	AA	AB	AA	AB	AB	AA	AB	AB	AA	AA	AB	AA	AB	AB	AA	AA	AB	AA	AA	AB	AA	AA	AB	AA
AB	AB	AB	AA	AB	AB	AA	AB	AB	AB	AB	AA	AB	AA	AB	AB	AB	AB	AA	AB	AB	AA	AB	AB	AB	AA	AA	AB	AB	AB	AA	AB	AB	AA	AB	AB	AB	AB	AB	AB	AA	AB	AB	AA	AB	AA	AA	AB	AB	AB	AA	AB	AB	AA	AB	AB	AB	AB	AB	AB	AB	AB	AA	AB	AA	AA	AB	AB	AB	AB	AB	AA	AB	AB	AB	AB	AB
AB	AA	AB	AB	AB	AB	AB	AB	AA	AB	AB	AB	AB	AA	AB	AB	AB	AB	AA	AB	AB	AA	AB	AB	AB	AA	AB	AB	AB	AB	AB	AB	AB	AB	AB	AB	AB	AA	AB	AB	AB	AA	AB	AB	AB	AB	AA	AB	AB	AB	AB	AB	AB	AB	AB	AB	AB	AB	AB	AB	AB	AB	AB	AB	AA	AB	AB	AB	AA	AB	AB	AB	AB	AB	AB	AB	AA
-	-	AA	AA	AA	AA	-	AB	-	AA	AB	AB	AB	AA	-	AA	AA	AA	AB	-	AA	AA	AB	AB	AB	AA	AA	AB	AA	-	AA	-	AB	-	AB	AB	-	-	-	-	-	AA	-	-	-	-	AB	AA	AB	AA	AB	AA	AA	AA	AA	-	-	AB	-	-	AA	AB	-	-	AB	AA	-	-	AA	AB	AB	AB	AB	-	-	AA	AB
AA	AA	AA	AA	AA	AA	AA	AA	AA	AA	AA	AA	AA	AA	AA	AA	AA	AA	AA	AA	AB	AA	AA	AA	AA	AA	AA	AA	AA	AA	AA	AA	AA	AA	AA	AA	AA	AA	AA	AA	AA	AA	AA	AA	AA	AA	AA	AA	AA	AA	AA	AA	AA	AA	AA	AA	AA	AA	AA	AA	AA	AA	AA	AA	AA	AA	AA	AA	AA	AA	AA	AA	AA	AA	AA	AA	AA
AB	AB	AA	AB	AA	AA	AB	AB	AA	AB	AB	AA	AB	AA	AB	AA	AB	AB	AB	AA	AB	AB	AA	AA	AA	AA	AB	AA	AB	AA	AB	-	AB	AA	AA	AA	AB	AB	AB	AB	AA	AA	-	AA	AA	AA	AB	-	AA	AA	AB	AB	AA	AB	AA	AA	AB	AA	AA	AA	AA	AA	AA	AA	AA	AA	AB	-	AA	AA	AA	AB	AA	AA	AA	AB	AB

snp_B2266 8	snp_B2298 8	snp_B2302 8	snp_B2315 8	snp_B2322 8	snp_B2333 8	snp_B2361 8	snp_B2365 8	snp_B2386 8	snp_B2399 8	snp_B2404 8	snp_B2406 8	snp_B2408 8	snp_B2436 8	snp_B2455 8	snp_B2476 8	snp_B2491 8
--	--	--	--	--	--	--	--	--	LG10	--	--	--	--	--	--	--
AB	AA	AB	AB	AB	AA	AB	AA	AA	AB	AA	AB	AA	AA	AA	AA	AB
AA	AA	AA	AB	AB	AA	AB	AA	AA	AB	AA	AB	AB	AB	AA	AB	AA
AA	AA	AA	AB	AB	AB	AA	AA	AA	AB	AB	AB	AB	AB	AA	AB	AB
AA	AB	AB	AB	AB	-	AB	AA	AB	AA	AB	AB	AB	AB	AA	AB	AB
AB	AB	AA	AB	AB	AA	AB	AA	AA	AB	AA	AB	AB	AA	AA	AB	AB
AA	-	-	AB	AA	-	AB	AA	AA	AA	-	AA	AA	-	AA	AA	AA
AA	AA	AA	AB	AA	AA	AA	AA	AB	AA	AA	AB	AA	AB	AA	AA	AA
AB	AA	AA	AB	AA	AA	AA	AA	AA	AA	AA	AB	AA	AA	AB	AA	AA
AB	AA	AB	AA	AB	AA	AB	AA	AB	AA	AB	AB	AA	AA	AB	AB	AA
AB	AA	AA	AB	AA	AA	AB	AA	AA	AB	AB	AA	AB	AA	AA	AB	AB
AB	AB	AB	AB	AA	AA	AB	AB	AA	AA	AB	AB	AA	AA	AA	AA	AA
AA	AA	AB	AB	AA	AA	AA	AA	AA	AB	AA	AA	AA	AA	AA	AA	AA
AA	AB	AB	AB	AA	AB	AA	AA	AB	AA	AA	AA	AA	AA	AB	AA	AB
AA	AB	AB	AA	AA	AB	AB	AA	AB	AB	AA	AA	AA	AA	AB	AB	AA
AB	AB	AB	AA	AB	AA	AB	AA	AB	AA	AB	AB	AA	AA	AA	AB	AA
AA	AB	AB	AB	AB	AA	AB	AA	AB	AA	AB	AA	AB	AB	AA	AB	AB
AA	AB	AB	AB	AB	AB	AB	AB	AB	AA	AB	AA	AB	AB	AB	AB	AB
AA	-	AA	AA	AB	-	-	AB	-	AA	-	AA	AA	AA	-	AB	-
AA	AA	AA	AA	AA	AA	AA	AA	AA	AA	AA	AA	AA	AA	AA	AA	AA
AB	AB	AA	AA	AA	AA	AA	AA	AB	AA	AA	AA	AB	AB	AB	AA	AA
AB	AB	AB	AB	AB	AB	AB	AB	AB	AB	AB	AB	AB	AB	AB	AB	AA
AB	AA	AA	AA	AA	AA	AA	AA	AA	AA	AA	AB	AA	AA	AA	AA	AA
AA	-	-	-	AA	-	-	AB	AA	AB	AB	AB	AA	-	-	AB	AA
AA	AA	AA	AA	AB	AB	AA	AB	AB	AA	AB	AB	AB	AA	AB	AB	AB
AB	AA	AA	AA	AB	AB	AA	AB	AB	AA	AB	AB	AB	AA	AB	AB	AB
AB	AA	AA	AB	AB	AA	AB	AA	AA	-	AB	AB	AA	AB	AB	AB	AB
AA	AB	AA	AB	AB	AA	AA	AA	AA	AA	AB	AB	AB	AA	AA	AA	AB
AB	AB	AB	AB	AA	AB	AB	AB	AB	AB	AB	AB	AB	AB	AB	AB	AA
AA	AB	AB	AA	AB	AB	AA	AB	AB	AA	AB	AB	AB	AA	-	AA	AA
AB	-	-	AB	AA	AA	AA	AA	AA	-	AA	-	AA	AA	-	AA	-
AB	AB	AB	AB	AB	AB	AB	AB	AB	AB	AB	AB	AB	AB	AB	AB	AB
AB	AA	AB	AB	AB	AB	AB	AB	AA	AB	AB	AB	AA	AB	AB	AB	AA
AB	AB	AB	-	AA	-	AB	AA	AA	AA	AB	AB	AA	AB	AA	AB	AA
AB	-	AB	-	AA	AB	AB	AB	AB	AA	AB	AA	AB	AB	-	AB	AA
AB	-	AA	AB	AA	-	AB	AB	AA	AB	AB	AB	AA	AB	AB	AB	AA
AB	AB	AB	AB	AB	AB	AB	AB	AB	AB	AB	AB	AB	AB	AB	AB	AB
AA	AA	AA	AA	AA	AA	AA	AA	AA	AA	AA	AA	AA	AA	AA	AA	AA
AA	AB	AA	AA	AA	AA	AA	AA	AA	AA	AA	AA	AB	AA	-	AA	AB
AB	AA	AB	AA	AA	AB	AA	AB	AA	AB	AB	AB	AA	AB	AB	AA	AB
AB	-	AA	AB	AA	-	AA	AA	AA	AA	AA	AB	AA	AB	-	AA	AA
AB	AA	AA	AB	AB	AA	AB	AA	AA	AB	AB	AB	AA	AB	AB	AB	AB
AA	AB	AB	AB	AB	AB	AB	AB	AB	AB	AB	AB	AB	AA	AA	AB	AB
AA	AB	AB	AB	AB	AB	AB	AB	AB	AB	AB	AB	AA	AB	AA	AB	AB
AB	AB	AA	AB	AB	-	AA	AB	AB	AA	AB	AA	AA	AA	-	AA	AB
AB	AB	AA	AA	AA	AB	AA	AA	AB	AA	AA	AA	AA	AA	AA	AA	AB
AB	AA	AB	AA	AB	AB	AA	AB	AB	AB	AA	AA	AA	AB	AB	AB	AB
AA	AB	AA	AA	AB	AA	AB	AB	AA	AB	AA	AB	AB	AA	AA	AB	AA
AB	AA	AA	AB	AB	AB	AA	AB	AA	AA	AB	AA	AB	AB	AA	AB	AB
AA	AA	AB	AB	AB	AB	AB	AB	AA	AA	AB	AB	AA	AA	AB	AB	AB
AA	AB	AB	AA	AB	AB	AB	AB	AB	AA	AB	AA	AB	AB	AA	AB	AB
AA	AB	AB	AA	AB	AB	AB	AB	AB	AA	AB	AB	AB	AB	AA	AB	AB
AA	-	AA	AA	AB	-	AB	AA	AB	AB	AA	AB	AB	AB	AA	AB	AB
AA	AA	AB	AB	AB	AB	AB	AB	AA	AB	AB	AB	AA	AA	AB	AB	AB
AB	AB	AB	AB	AB	AB	AB	AB	AB	AB	AB	AB	AB	AB	AB	AB	AB

snp_B3456 8	snp_B3463 8	snp_B3469 8	snp_B3489 8	snp_B3500 8	snp_B3537 8	snp_B3571 8	snp_B3574 8	snp_B3582 8	snp_B3590 8	snp_B3623 8	snp_B3654 8	snp_B3700 8	snp_B3701 8	snp_B3800 8	snp_B3813 8	snp_B3837 8	snp_B3854 8	snp_B3856 8	snp_B3868 8	snp_B3884 8	snp_B3908 8	snp_B3919 8	snp_B3953 8	snp_B4003 8	snp_B4016 8	snp_B4038 8	snp_B4061 8	snp_B4083 8	snp_B4119 8	snp_B4131 8	snp_B4135 8	snp_B4166 8	snp_B4178 8	snp_B4182 8	snp_B4213 8	snp_B4235 8	snp_B4262 8	snp_B4304 8	snp_B4305 8	snp_B4313 8	snp_B4316 8	snp_B4340 8	snp_B4399 8	snp_B4401 8	snp_B4416 8	snp_B4429 8	snp_B4448 8	snp_B4484 8	snp_B4485 8	snp_B4486 8	snp_B4498 8	snp_B4534 8	snp_B4555 8	snp_B4634 8	snp_B4648 8	snp_B4659 8	snp_B4682 8	snp_B4689 8	snp_B4698 8	snp_B4708 8	snp_B4723 8	snp_B4750 8	snp_B4762 8	snp_B4856 8	snp_B4884 8	snp_B4895 8	snp_B4900 8	snp_B4906 8	snp_B4923 8	snp_B4951 8	snp_B4969 8	snp_B4980 8	snp_B4993 8	snp_B4998 8	snp_B5097 8	snp_B5132 8
--	--	--	--	LG10	--	--	--	--	--	--	--	LG10	--	--	--	--	--	--	--	--	--	--	--	--	--	--	--	--	--	--	--	--	--	--	--	--	--	--	--	--	--	--	--	--	--	--	--	--	--	LG10	--	--	--	--	--	--	--	--	--	--	--	--	--	--	--	--	--	--	--	--	--	--	--	--	--	--
AB	AB	AB	AA	AA	AB	AB	AB	AB	AA	AA	AB	AB	AA	AB	AA	AA	AA	AB	AB	AA	AA	AB	AB	AB	AA	AA	AB	AA	AB	AB	AB	AB	AB	AA	AA	AA	AB	AA	AB	AA	AA	AA	AA	AB	AB	AB	AB	AA	AA	AA	AB	AA	AB	AA	AA	AB	AA	AA	AA	AA	AA	AA	AA	AB	AB	AB	AA	AA	AA	AB	AA	AA	AA	AA	AA	AB
AB	AA	AA	AA	AB	AB	AA	AA	AB	AB	AB	AA	AB	AA	AB	AA	AB	AA	AA	AB	AB	AA	AB	AA	AB	AA	AA	AA	AB	AB	AA	AA	AA	AB	AB	AB	AA	AB	AA	AA	AA	AA	AA	AB	AB	AB	AB	AB	AA	AB	AB	AA	AB	AB	AA	AA	AA	AA	AA	AB	AB	AA	AA	AA	AB	AA	AB	AA	AB	AA	AA	AB	AA	AA	AA	AA	AB
AB	AA	AB	AA	AB	AA	AB	AA	AA	AA	AB	AA	AA	AB	AA	AA	AA	AA	AB	AA	AA	AB	AB	AB	AA	AA	AB	AA	AA	AA	AA	AA	AB	AA	AA	AA	AA	AA	AA	AB	AA	AB	AB	AA	AB	AB	AB	AB	AA	AA	AA	AB	AA	AB	AA	AA	AA	AB	AB	AB	AB	AA	AA	AA	AB	AA	AB	AA	AA	AA	AA	AB	AB	AA	AB	AB	AB
AB	AB	AB	AB	AB	AB	AB	AA	AB	AB	-	AB	AB	AA	AB	-	AB	AA	AA	AB	AB	AB	AB	AA	AA	AB	AB	AB	AB	AB	AA	AB	AA	AB	AB	AB	AA	AA	AB	AB	AB	AA	AA	AB	AB	AB	-	AB	AB	-	AB	AA	AB	AB	AA	AA	-	AB	AB	AB	AB	AB	AB	AB	AB	AB	AB	AB	AA	AB	AB	AA	AB	AA	AB	AA	AB

snp_B5143	snp_B5171	snp_B5175	snp_B5255	snp_B5297	snp_B5309	snp_B5314	snp_B5364	snp_B5380	snp_B5382	snp_B5395	snp_B5409	snp_B5425	snp_B5442	snp_B5465	snp_B5473	snp_B5488
8	8	8	8	8	8	8	8	8	8	8	8	8	8	8	8	8
--	--	--	--	--	--	--	--	--	--	--	--	--	--	--	--	--
AA	AA	AB	AB	AB	AB	AA	AA	AA	AB	AA	AA	AA	AB	AA	AA	AB
AA	AB	AB	AB	AA	AA	AB	AA	AB	AA	AA	AA	AA	AB	AB	AB	AB
AA	AB	AB	AB	AB	AA	AB	AA	AA	AA	AA	AB	AB	AB	AA	AB	AA
AB	AB	-	AB	AA	AA	AB	AA	AA	AB	AA	-	AA	AB	AA	AB	AB
AA	AB	AB	AB	AB	AA	AB	AB	AB	AB	AB	AB	AA	AB	AA	AB	AB
AA	AA	-	AB	AB	AB	AA	-	AB	AB	AA	-	AA	-	AB	AA	AA
AB	AA	AA	AA	AA	AA	AA	AA	AA	AA	AB	AA	AA	AB	AA	AA	AB
AA	AA	AA	AA	AA	AA	AA	AA	AB	AA	AB	AA	AA	AB	AB	AA	AB
AA	AB	AA	AB	AA	AB	AB	AA	AA	AB	AA	AA	AA	AA	AA	AA	AA
AA	AB	AA	AB	AB	AA	AB	AA	AA	AB	AB	AA	AA	AB	AA	AB	AB

snp_B5537	snp_B5540	snp_B5557	snp_B5571	snp_B5583	snp_B5586	snp_B5624	snp_B5630	snp_B5637	snp_B5664	snp_B5687	snp_B5718	snp_B5725	snp_B5753	snp_B5757	snp_B5759	snp_B5898	snp_B5936	snp_B6001	snp_B6150	snp_B6160
8	8	8	8	8	8	8	8	8	8	8	8	8	8	8	8	8	8	8	8	8
--	--	--	--	--	--	--	LG10	--	--	--	--	--	--	--	--	--	--	--	--	--
AB	AB	AA	AB	AA	AB	AB	AB	AA	AB	AA	AA	AA	AA	AB	AA	AB	AA	AB	AA	AA
AA	AA	AA	AB	AB	AA	AA	AB	AA	AB	AA	AB	AB	AA	AB	AA	AB	AA	AA	AA	AB
AA	AA	AB	AA	AA	AA	AA	AB	AA	AA	AB	AB	AA	AA	AA	AB	AA	AB	AA	AB	AB
-	AA	AA	AB	AB	AA	AA	AB	AB	AB	AA	AB	AA	AB	AB	-	AB	AA	AB	AA	AB
AB	AA	AB	AB	AA	AB	AA	AB	AA	AA	AA	AB	AB	AA	AA	AB	AA	AA	AA	AA	AB
AB	AA	AA	AA	AA	AB	AB	AB	-	AA	AA	AB	AB	AB	AB	-	AA	AA	AB	AA	-
AA	AA	AA	AA	AA	AA	AA	AA	AB	AA	AA	AA	AA	AB	AA	AA	AA	AB	AB	AB	AA
AA	AA	AA	AA	AA	AA	AA	AA	AA	AB	AA	AA	AB	AB	AA	AA	AA	AA	AA	AB	AA
AA	AB	AA	AA	AB	AB	AA	AB	AA	AB	AA	AA	AA	AA	AA	AA	AA	AA	AB	AA	AA
AB	AA	AA	AA	AA	AA	AA	AB	AA	AB	AA	AB	AB	AA	AA	AA	AA	AA	AA	AA	AB

snp_B0002	snp_B0006	snp_B0084	snp_B0090	snp_B0091	snp_B0115	snp_B0173	snp_B0190	snp_B0222	snp_B0238	snp_B0305	snp_B0403	snp_B0465	snp_B0481	snp_B0523	snp_B0532	snp_B0533	snp_B0555	snp_B0577	snp_B0600	snp_B0617
9	9	9	9	9	9	9	9	9	9	9	9	9	9	9	9	9	9	9	9	9
--	--	--	--	--	--	--	LG2	--	--	--	--	--	LG2	--	--	--	--	LG2	--	--
AA	AA	AA	AB	AA	AA	AA	AB	AB	AB	AA	AB	AB	AB	AA	AB	AB	AA	AB	AA	AA
AB	AA	AB	AB	AB	AA	AA	AB	AB	AB	AA	AB	AB	AB	AB	AB	AB	AA	AB	AA	AB
AA	AB	AA	AB	AA	AB	AB	AA	AB	AB	AB	AA	AB	AA	AA	AA	AB	AB	AA	AB	AA
-	AA	AA	AB	AB	AB	-	AA	AA	AA	AB	AB	AB	AB	AB	-	AB	AB	-	AA	AB
AA	AA	AB	AB	AA	AA	AA	AB	AA	AB	AA	AB	AB	AA	AB	AB	AB	AA	AA	AA	AA
-	-	AA	AA	AB	-	-	AA	AB	AA	-	AA	-	-	AA	AA	AB	AB	-	AB	-
AB	AB	AB	AA	AB	AB	AB	AB	AB	AB	AB	AB	AB	AB	AB	AB	-	AB	AB	AB	AB
AB	AB	AB	AA	AB	AB	AB	AB	AB	AA	AB	AA	AA	AA	AB	AA	AA	AB	AA	AB	AA
AB	AB	AA	AA	AA	AB	AB	AA	AB	AA	AB	AA	AB	AA	AB	AA	AA	AB	AA	AB	AA
AB	AB	AB	AB	AB	AB	AA	AA	AA	AA	AB	AB	AA	AA	AA	AA	AB	AA	AA	AB	AA

snp_B0641	snp_B0649	snp_B0662	snp_B0710	snp_B0713	snp_B0719	snp_B0739	snp_B0741	snp_B0755	snp_B0764	snp_B0793	snp_B0864	snp_B0876	snp_B0902	snp_B0905	snp_B0922	snp_B0952	snp_B0980
9	9	9	9	9	9	9	9	9	9	9	9	9	9	9	9	9	9
--	--	--	--	--	--	--	--	--	--	--	--	--	--	--	--	--	--
AB	AA	AA	AA	AA	AA	AA	AB	AB	AA	AA	AB	AB	AA	AA	AB	AA	AA
AB	AA	AA	AA	AB	AA	AB	AB	AB	AA	AB	AB	AB	AB	AA	AB	AB	AB
AA	AB	AA	AB	AA	AB	AA	AB	AA	AB	AB	AB	AB	AA	AB	AA	AA	AA
AA	AB	-	AB	AB	AB	-	AA	AB	AB	AB	AA	AA	AA	AB	AB	AB	AB
AB	AA	AB	AB	AA	AA	AB	AB	AA	AA	AA	AB	AA	AA	AA	AB	AB	AB
-	AB	AA	AA	-	AB	AA	AA	AB	AB	AB	-	-	-	AB	AB	AA	AB
AA	AB	AB	AB	AB	AB	AB	AB	AB	AB	AB	AA	AB	AB	AB	AB	AB	AB
AB	AB	AB	AB	AA	AB	AB	AA	AA	AB	AA	AB	AB	AB	AB	AA	AA	AA
AB	AA	AB	AA	AA	AB	AA	AA	AA	AB	AA	AB	AB	AA	AB	AB	AA	AA
AB	AA	AB	AB	AB	AB	AA	AA	AA	AA	AB	AA	AA	AA	AA	AA	AA	AA

snp_B10199	snp_B10779	snp_B10899	snp_B11119	snp_B11169	snp_B11499	snp_B11699	snp_B11729	snp_B11829	snp_B11859	snp_B11889	snp_B11969	snp_B11979	snp_B12099	snp_B12689	snp_B12769	snp_B12979
LG2	--	--	--	--	--	--	--	--	--	--	--	--	LG2	--	--	--
AB	AB	AB	AA	AB	AB	AB	AB	AB	AB	AA	AB	AA	AB	AA	AB	AB
AB	AB	AB	AB	AB	AB	AB	AB	AB	AA	AB	AA	AB	AB	AB	AB	AB
AA	AB	AA	AA	AA	AA	AA	AA	AA	AA	AB	AA	AA	AB	AB	AB	AA
AB	AA	AB	AB	AB	AB	AB	AB	AB	AB	AB	-	-	AA	AA	AB	AB
AA	AB	AA	AA	AB	AA	AB	AA	AA	AA	AA	AA	AA	AA	AA	AA	AA
AA	AB	-	-	AA	AA	AA	-	-	-	-	AB	-	AB	AA	AB	-
AB	AB	AB	AB	AB	AB	AB	AB	AB	AB	AB	AB	AB	AA	AB	AB	AB
AA	AB	AA	AA	AA	AA	AA	AA	AA	AB	AA	AB	AB	AB	AB	AA	AA
AA	AB	AA	AB	AA	AB	AB	AA	AA	AB	AA	AA	AB	AB	AA	AA	AA
AA	AA	AA	AB	AB	AB	AB	AA	AA	AA	AA	AA	AB	AB	AA	AB	AB
AB	AA	AB	AA	AB	AB	AB	AB	AB	AB	AB	AB	AA	AA	AB	AB	AA
AB	AA	AA	AA	AA	AA	AA	AB	AA	AB	AB	AB	AA	AB	AA	AB	AB
AB	AB	AB	AB	AB	AB	AB	AB	AB	AA	AB	AA	AA	AB	AB	AB	AB
AB	AA	AB	AB	AB	AA	AB	AB	AB	AB	AA	AB	AB	AA	AA	AA	AB
AB	AB	AB	AB	AB	AA	AA	AB	AB	AB	AA	AB	AA	AA	AA	AA	AB
AB	AB	AB	AA	AB	AA	AA	AA	AB	AA	AB	AB	AB	AA	AA	AA	AA
AA	AA	AB	AB	AA	AA	AB	AA	AB	AA	AB	AA	AB	AB	AA	AB	AB
AB	AA	-	-	AA	-	AB	AB	AB	-	-	-	-	AA	AA	AA	AB
AB	AA	AB	AA	AA	AA	AA	AB	AB	AB	AB	AB	AA	AB	AA	AB	AB
AB	AA	AA	AB	AB	AA	AA	AB	AA	AB	AA	AB	AB	AA	AA	AA	AA
AA	AA	AA	AB	AB	AB	AB	AB	AA	AA	AA	AB	AB	AB	AA	AB	AB
AA	AB	AA	AA	AB	AB	AB	AB	AA	AA	AB	AB	AB	AB	AB	AB	AA
AB	AB	AB	-	AB	-	AB	AB	AB	-	-	-	-	AA	AA	AA	AB
AA	AB	AA	AA	AA	AA	AA	AB	AA	AA	AA	AB	AA	AB	AB	AB	AA
AB	AA	AB	AB	AA	AA	AA	AB	AB	AB	AA	AB	AA	AB	AA	AB	AB
AA	AB	AA	AA	AB	AB	AB	AA	AA	AA	AA	AA	AA	AA	AB	AA	AA
AB	AB	AB	AB	AB	AB	AA	AB	AB	AB	AA	AA	AA	AB	AB	AB	AB
AB	AB	AB	AB	AB	AB	AA	AB	AB	AB	AA	AA	AB	AB	AB	AB	AB
AB	AA	AA	AA	AB	AB	AB	AA	AA	AA	AA	AA	AB	AA	AB	AB	AA
-	AA	AA	-	AB	-	AB	AB	AA	-	AB	AB	-	AA	AB	AB	AB
AB	AA	AA	AA	AB	AA	AB	AB	AA	AB	AA	AB	AB	AA	AA	AA	AA
AA	AB	AB	AA	AB	AB	AA	AA	AB	AA	AB	AA	AA	AB	AB	AB	AB

snp_B13099	snp_B13189	snp_B13249	snp_B13349	snp_B13459	snp_B13539	snp_B13739	snp_B13849	snp_B14029	snp_B14099	snp_B14179	snp_B14209	snp_B14259	snp_B14619	snp_B14949	snp_B15279	snp_B15449	snp_B15499	snp_B15549	snp_B15769	snp_B16059	snp_B16289	snp_B16339	snp_B16549	snp_B16869	snp_B16909	snp_B16949	snp_B17519	snp_B17639	snp_B17879
--	--	--	LG2	--	--	--	--	--	--	LG2	--	--	LG2	--	--	--	--	--	--	--	--	--	--	--	--	--	--	--	--

snp_B17999	snp_B18299	snp_B19079	snp_B19379	snp_B19519	snp_B19589	snp_B20329	snp_B20459	snp_B20759	snp_B20849	snp_B20969	snp_B21289	snp_B21659	snp_B21899	snp_B22399	snp_B22409	snp_B22469	snp_B23419	snp_B23559	snp_B23699	snp_B23819	snp_B23849	snp_B24729	snp_B24839	snp_B24979	snp_B24989	snp_B25129	snp_B25399	snp_B25659	snp_B25709
--	--	--	--	--	--	--	--	--	--	--	--	--	--	--	--	--	--	--	--	LG2	--	--	--	--	--	--	--	--	--

snp_B2595 9	snp_B2617 9	snp_B2689 9	snp_B2719 9	snp_B2728 9	snp_B2740 9	snp_B2775 9	snp_B2782 9	snp_B2832 9	snp_B2835 9	snp_B2867 9	snp_B2879 9	snp_B2909 9	snp_B2934 9	snp_B2938 9	snp_B2942 9	snp_B2952 9
--	--	--	--	--	LG2	--	LG2	--	--	--	--	--	--	--	--	--
AB	AA	AB	AA	AB	AB	AA	AB	AB	AA	AA	AB	AB	AA	AA	AB	AA
AA	AB	AA	AB	AB	AB	AA	AB	AB	AA	AB	AA	AB	AA	AA	AB	AB
AB	AB	AB	AB	AB	AA	AA	AA	AA	AA	AA	AB	AA	AB	AA	AA	AA
AB	-	AB	AB	AA	AB	AB	AB	AB	AB	AA	AB	AB	AB	AA	AB	AB
AA	AA	AA	AA	AB	AB	AA	AB	AA	AB	AB	AB	AA	AA	AA	AB	AB
-	-	AB	AB	-	AA	AA	AA	AB	AB	AB	AA	AA	AB	-	AA	AB
AB	AB	AB	AB	AB	AA	AB	AB	AB	AA	AB	AB	AA	AB	AA	AB	AB
AB	AA	AB	AB	AB	AA	AB	AA	AA	AA	AB	AB	AA	AB	AB	AB	AA
AB	AB	AB	AB	AA	AA	AB	AA	AB	AA	AA	AA	AA	AB	AA	AB	AA
AB	AA	AB	AB	AA	AA	AA	AA	AB	AA	AA	AB	AA	AA	AB	AA	AA
AA	AA	AA	AA	AA	AA	AB	AB	AB	AB	AB	AA	AB	AB	AB	AA	AB
AA	AA	AB	AB	AA	AA	AB	AB	AA	AA	AB	AA	AB	AB	AA	AA	AA
AA	AB	AA	AA	AB	AB	AA	AB	AB	AA	AB	AA	AB	AA	AB	AA	AB
AB	AB	AA	AA	AA	AA	AB	AB	AB	AB	AB	AA	AB	AA	AB	AB	AA
AA	AB	AA	AA	AA	AB	AA	AB	AB	AA	AA	AA	AB	AA	AA	AA	AA
AB	AA	AA	AA	AB	AB	AA	AB	AB	AB	AB	AA	AB	AA	AB	AA	AA
AB	AB	AB	AB	AA	AA	AA	AB	AB	AB	AB	AB	AB	AB	AB	AB	AA
-	-	AA	AA	AB	AA	AA	AA	AB	AA	AA	AA	AB	AB	-	AA	AB
AB	AB	AB	AB	AA	AA	AB	AB	AB	AA	AA	AA	AB	AB	AA	AA	AA
AA	AA	AA	AA	-	AB	AB	AB	AA	AA	AA	AB	AA	AA	AA	AB	AA
AB	AA	AB	AB	AA	AA	AA	AA	AB	AA	AA	AB	AA	AA	AB	AA	AA
AB	AB	AB	AB	AA	AA	AA	AA	AB	AA	AA	AA	AB	AB	AB	AB	AA
-	-	AB	-	-	AB	AA	AB	AB	AB	AB	AA	AB	AB	AB	AA	AB
AA	AA	AB	AB	AB	AA	AA	AA	AA	AB	AB	AA	AA	AA	AB	AA	AB
AB	AA	AB	AB	AB	AA	AB	AB	AA	AB	AB	AA	AB	AA	AA	AA	AB
AA	AA	AA	AA	AB	AB	AA	AA	AA	AA	AA	AA	AA	AA	AA	AB	AB
AB	AB	AA	AA	AB	AB	AB	AB	AB	AA	AB	AB	AB	AA	AB	AB	AA
AB	AB	AA	AA	AB	AB	AB	AB	AB	AB	AB	AB	AB	AA	AB	AB	AA
AB	AA	AB	AB	AB	AA	AB	AA	AA	AB	AB	AA	AA	AA	AB	AB	AB
-	-	AA	AA	-	AB	AB	AB	AB	AB	AB	AA	AB	AB	AB	AA	AB
AA	AA	AA	AA	AA	AB	AB	AB	AA	AB	AB	AA	AA	AA	AB	AB	AA
AB	AB	AB	AB	AB	AB	AA	AA	AB	AA	AA	AB	AB	AB	AA	AA	AB
-	-	AB	AB	-	AB	AB	AB	AB	AA	AA	AB	AB	AB	AA	AA	AA
-	AA	AA	AA	AA	AB	AB	AB	AA	AA	AA	AB	AB	AA	AB	AB	AA
AB	AB	AA	AA	AB	AB	AB	AB	AB	AB	AB	AB	AB	AB	-	AB	AB
AA	-	AA	-	AA	AA	AA	AA	AA	AA	AA	AA	AA	AA	AA	AB	AA
AB	AB	AB	AB	AA	AB	AB	AB	AB	AA	AB	AB	AB	AB	AB	AB	AB
AB	AB	AB	AB	AA	AB	AA	AB	AB	AA	AB	AB	AB	AB	AB	AB	AA
AB	AB	AB	AB	AB	AA	AA	AA	AB	AA	AB	AA	AA	AA	AB	AA	AB
-	AB	AA	AA	-	AA	AA	AA	AB	AA	AA	AA	AA	AA	AA	AA	AA
AA	AB	AB	AB	AB	AA	AB	AA	AA	AA	AA	AA	AA	AB	AA	AB	AB
AA	AA	AA	AA	AA	AA	AA	AA	AA	AB	AB	AB	AA	AA	AB	AB	AA
AA	AA	AA	AA	-	AA	AA	AA	AA	AA	AB	AB	AA	AA	AB	AB	AB
AB	AA	AA	AA	AB	AB	AA	AA	AA	AB	AB	AA	AB	AB	AB	AB	AB
AA	AA	AA	AA	AB	AA	AA	AA	AA	AA	AA	AA	AA	AA	AB	AB	AA
AB	AB	AA	AA	AA	AA	AA	AA	AB	AA	AA	AB	AA	AA	AA	AA	AA
AB	AA	AB	AB	AB	AA	AB	AA	AA	AB	AB	AA	AA	AB	AA	AA	AA
AB	AB	AB	AB	AB	AA	AA	AA	AB	AA	AB	AB	AA	AB	AA	AA	AB
AA	AA	AA	AA	AA	AA	AA	AA	AA	AA	AA	AA	AA	AA	AA	AA	AA
AB	AA	AB	AB	AB	AA	AB	AA	AA	AA	AB	AB	AA	AB	AB	AA	AA
-	AA	AB	AB	AB	AA	AB	AA	AA	AA	AA	AA	AB	AB	AA	AA	AB
AA	AA	AA	AA	AB	AB	AB	AA	AA	AA	AA	AA	AA	AA	AA	AA	AB

snp_B3916	snp_B3930	snp_B3939	snp_B3970	snp_B3994	snp_B3995	snp_B4029	snp_B4031	snp_B4033	snp_B4046	snp_B4047	snp_B4068	snp_B4075	snp_B4090	snp_B4095	snp_B4103	snp_B4134	snp_B4139	snp_B4165	snp_B4245
9	9	9	9	9	9	9	9	9	9	9	9	9	9	9	9	9	9	9	9
--	--	--	--	--	--	--	--	--	--	--	--	--	--	--	--	--	LG2	--	--
[illegible]	[illegible]	[illegible]	[illegible]	[illegible]	[illegible]	[illegible]	[illegible]	[illegible]	[illegible]	[illegible]	[illegible]	[illegible]	[illegible]	[illegible]	[illegible]	[illegible]	[illegible]	[illegible]	[illegible]

snp_B4258	snp_B4261	snp_B4278	snp_B4286	snp_B4288	snp_B4329	snp_B4345	snp_B4380	snp_B4387	snp_B4414	snp_B4426	snp_B4443	snp_B4449	snp_B4467	snp_B4483	snp_B4510	snp_B4517	snp_B4557	snp_B4574	snp_B4596
9	9	9	9	9	9	9	9	9	9	9	9	9	9	9	9	9	9	9	9
--	--	--	--	--	--	--	--	--	LG2	--	--	--	--	--	--	--	LG2	--	--
[illegible]	[illegible]	[illegible]	[illegible]	[illegible]	[illegible]	[illegible]	[illegible]	[illegible]	[illegible]	[illegible]	[illegible]	[illegible]	[illegible]	[illegible]	[illegible]	[illegible]	[illegible]	[illegible]	[illegible]

snp_B4608	snp_B4610	snp_B4619	snp_B4736	snp_B4741	snp_B4833	snp_B4843	snp_B4882	snp_B4886	snp_B4888	snp_B4913	snp_B4918	snp_B4962	snp_B5028	snp_B5040	snp_B5064	snp_B5072
9	9	9	9	9	9	9	9	9	9	9	9	9	9	9	9	9
--	--	--	--	--	--	--	LG2	LG2	--	--	--	--	--	--	--	--
[illegible]	[illegible]	[illegible]	[illegible]	[illegible]	[illegible]	[illegible]	[illegible]	[illegible]	[illegible]	[illegible]	[illegible]	[illegible]	[illegible]	[illegible]	[illegible]	[illegible]

snp_B5075	snp_B5123	snp_B5130	snp_B5167	snp_B5218	snp_B5249	snp_B5259	snp_B5290	snp_B5317	snp_B5321	snp_B5329	snp_B5341	snp_B5350	snp_B5355	snp_B5390	snp_B5393	snp_B5421	snp_B5427	snp_B5452	snp_B5476
9	9	9	9	9	9	9	9	9	9	9	9	9	9	9	9	9	9	9	9
LG2	--	--	LG2	--	--	LG2	LG2	--	--	--	--	--	--	--	--	--	--	--	--
[illegible]	[illegible]	[illegible]	[illegible]	[illegible]	[illegible]	[illegible]	[illegible]	[illegible]	[illegible]	[illegible]	[illegible]	[illegible]	[illegible]	[illegible]	[illegible]	[illegible]	[illegible]	[illegible]	[illegible]

snp_B5490	snp_B5499	snp_B5505	snp_B5528	snp_B5535	snp_B5559	snp_B5576	snp_B5577	snp_B5589	snp_B5594	snp_B5607	snp_B5625	snp_B5643	snp_B5644	snp_B5677	snp_B5696	snp_B5705	snp_B5741	snp_B5822	snp_B5836
9	9	9	9	9	9	9	9	9	9	9	9	9	9	9	9	9	9	9	9
--	--	--	--	--	--	--	--	--	--	LG2	--	--	LG2	--	--	--	--	--	--
AB	AB	AA	AA	AB	AB	AB	AA	AB	AA	AB	AA	AB	AB	AA	AA	AB	AB	AB	AA
AB	AB	AA	AA	AB	AB	AA	AB	AB	AB	AB	AB	AB	AA	AA	AA	AB	AA	AB	AA
AA	AB	AA	AA	AB	AA	AA	AA	AB	AA	AA	AA	AA	AB	AA	AB	AB	AB	AA	AB
AB	AB	AB	AA	AB	AA	AB	AB	AB	AA	AB	AA	AB	AB	AA	AA	AB	AB	-	AA
AA	AB	AA	AA	AB	AA	AA	AB	AA	AB	AA	AA	AB	AB	AA	AA	AA	AA	AB	AA
AA	AA	AB	-	AA	AB	AA	AA	AA	-	AA	AB	AA	AA	AB	AA	AA	AA	AA	AB
AB	AB	AB	AB	AB	AA	AB	AB	AB	AB	AB	AA	AB	AB	AB	AB	AA	AB	AB	AB
AB	AA	AB	AB	AA	AB	AA	AA	AB	AA	AA	AB	AA	AB	AA	AB	AB	AB	AA	AB
AB	AA	AB	AA	AA	AB	AA	AA	AA	AA	AA	AA	AA	AB	AB	AB	AB	AB	AA	AB
AA	AA	AB	AA	AB	AA	AB	AA	AA	AA	AA	AB	AA	AA	AB	AA	AB	AB	AA	AB
AA	AB	AB	AB	AB	AB	AB	AA	AB	AB	AB	AB	AA	AA	AA	AB	AB	AB	AB	AA
AA	AA	AA	AA	AB	AA	AB	AA	AB	AA	AB	AA	AA	AA	AA	AB	AA	AA	AB	AB
AA	AB	AA	AA	AB	AB	AA	AB	AB	AB	AB	AB	AB	AA	AA	AA	AB	AA	AB	AA
AB	AA	AA	AA	AB	AA	AB	AA	AA	AB	AB	AB	AA	AA	AA	AB	AA	AB	AB	AA
AA	AB	AA	AB	AA	AA	AB	AB	AB	AA	AB	AB	AB	AB	AA	AA	AB	AB	AB	AA
AA	AB	AA	AB	AA	AB	AA	AA	AB	AB	AB	AB	AA	AB	AA	AB	AA	AB	AB	AA
AA	AA	AB	AB	AB	AB	AB	AA	AA	AA	AA	AB	AA	AA	AB	AA	AA	AB	AB	AB
AB	AA	-	-	AB	AA	-	-	AB	AB	AB	-	AA	-	AA	AA	AA	AB	AA	AA
AA	AA	AA	AB	AA	AA	AA	AA	AB	AA	AB	AA	AA	AA	AB	AB	AA	AA	AB	AB
AA	AB	AA	AA	AB	AB	AA	AB	AB	AA	AB	AA	AA	AB	AA	AB	AA	AA	AB	AA
AA	AA	AB	AB	AB	AA	AB	AA	AA	AA	AA	AB	AA	AA	AB	AA	AA	AB	AA	AB
AB	AB	AA	AB	AB	AB	AB	AB	AA	AB	AA	AB	AB	AB	AB	AA	AB	AB	AA	AB
AA	AB	AB	AA	AB	AB	AB	-	AA	AB	AB	-	-	-	AA	AB	-	-	-	AA
AA	AA	AA	AB	AA	AB	AA	AA	AA	AA	AA	AB	AA	AA	AB	AA	AA	AA	AA	AB
AA	AA	AA	AB	AA	AA	AB	AA	AB	AA	AB	AA	AA	AA	AB	AB	AA	AA	AB	AB
AA	AB	AA	AA	AB	AB	AA	AA	AA	AB	AA	AA	AB	AB	AA	AA	AB	AB	AA	AA
AA	AB	AA	AA	AB	AB	AA	AB	AB	AA	AB	AB	AB	AB	AA	AA	AB	AB	AB	AA
AB	AB	AA	AA	AB	AB	AA	AB	AB	AB	AB	AB	AB	AA	AA	AA	AB	AB	AB	AA
AB	AA	AA	AA	AB	AB	AA	AA	AB	AB	AA	AA	AA	AA	AB	AA	AB	AB	AA	AB
AB	AA	AA	AB	AA	AA	AA	AB	AB	-	AB	AA	AB	AB	AB	AA	AB	AB	-	AB
AB	AB	AA	AA	AB	AB	AA	AB	AB	AB	AB	AB	AB	AB	AA	AB	AA	AA	AA	AA
AB	AB	AA	AB	AA	AB	AA	AB	AA	AB	AA	AA	AB	AB	AB	AA	AB	AA	AA	AB
AB	AB	AB	AB	AA	AA	AB	AB	AB	AB	AB	AA	AA	-	AB	AB	AA	AB	AB	AB
AA	AB	AA	AA	AB	AB	AA	AB	AB	AB	AB	AB	AB	AB	AA	AA	AB	AB	AB	AA
AB	AB	AB	AB	AB	AB	AB	-	AA	AB	AB	-	AA	AB	AA	AA	AB	AB	AB	AA
AA	AA	AA	AA	AA	AA	AA	AA	AA	AA	AA	AA	AA	AA	AA	AA	AA	AA	AA	AA
AB	AB	AB	AB	AB	AA	AB	AB	AB	AB	AB	AA	AB	AB	AB	AB	AB	AB	AB	AB
AA	AA	AB	AB	AB	AA	AB	AA	AA	AA	AA	AA	AA	AA	AB	AA	AA	AB	AB	AB
AA	AB	AB	AB	AB	AA	AB	AB	AB	AB	AA	AB	AA	AA	AB	AA	AA	AB	AA	AB
AA	AA	AB	AA	AA	AA	AB	AA	AA	AA	AA	AA	-	AA	AB	AA	AA	AA	-	AA
AA	AA	AB	AA	AB	AB	AB	AA	AA	AB	AA	AA	AA	AA	AB	AA	AB	AA	AA	AB
AA	AA	AA	AA	AA	AB	AA	AA	AA	AA	AA	AA	AA	AA	AB	AA	AA	AA	AA	AB
AA	AA	AA	AA	AB	AB	AA	AA	AA	AA	AA	AB	AA	AA	AA	AA	AA	AB	AA	AA
AA	AA	AA	AA	AA	AB	AA	AA	AA	AB	AA	AB	AA	AA	AB	AA	AB	AA	AA	AA
AB	AB	AB	AA	AB	AB	AB	AA	AA	AB	AB	AA	AA	AA	AA	AA	AB	AB	AA	AA
AB	AA	AB	AA	AA	AB	AA	AB	AA	AB	AA	AB	AB	AA	AA	AA	AB	AB	AA	AA
AA	AB	AB	AA	AA	AB	AB	AA	AA	AB	AA	AA	AA	AA	AB	AA	AB	AA	AA	AA
AA	AB	AA	AB	AA	AA	AB	AB	AB	AA	AB	AA	AB	AB	AB	AB	AA	AB	AB	AB
AB	AA	AB	AB	AA	AA	AB	AA	AA	AB	AA	AA	AA	AA	AB	AB	AA	AB	AB	AB
AA	AA	AA	AA	AA	AA	AA	AA	AB	AB	AA	AA	AB	AA	AA	AB	AA	AA	AB	AB
AB	AA	AB	AB	AA	AB	AB	AA	AA	AA	AA	AB	AA	AB	AB	AB	AB	AB	AA	AB
AA	AB	AB	AA	AB	AB	AB	-	AA	AA	AA	AA	AA	AB	AA	AB	AB	AB	-	AB
AB	AB	AA	AA	AB	AB	AA	AB	AB	AB	AB	AA	AB	AA	AB	AB	AA	AB	AA	AA
AA	AA	AB	AB	AB	AB	AB	AA	AA	AA	AA	AB	AA	AA	AB	AA	AA	AB	AB	AB

[illegible]

snp_B1250	snp_B1251	snp_B1278	snp_B1293	snp_B1306	snp_B1326	snp_B1342	snp_B1363	snp_B1366	snp_B1401	snp_B1421	snp_B1447	snp_B1448	snp_B1453	snp_B1515	snp_B1525	snp_B1537	snp_B1550	snp_B1579	snp_B1580	snp_B1583	snp_B1587	snp_B1651	snp_B1678	snp_B1701	snp_B1728	snp_B1731	snp_B1733	snp_B1735	snp_B1785	snp_B1823	snp_B1844	snp_B1850	snp_B1852	snp_B1856	snp_B1857	snp_B1878	snp_B1899	snp_B1900
10	10	10	10	10	10	10	10	10	10	10	10	10	10	10	10	10	10	10	10	10	10	10	10	10	10	10	10	10	10	10	10	10	10	10	10	10	10	10
--	LG4	--	--	--	--	--	--	--	--	--	--	--	--	--	--	--	LG4	--	LG4	--	--	--	--	--	--	--	--	--	--	--	--	--	--	--	--	--	--	--
AB	AA	AA	AB	AA	AA	AA	AA	AB	AB	AB	AA	AB	AA	AA	AA	AB	AA	AB	AB	AA	AB	AB	AB	AA	AB	AB	AB	AA	AA	AA	AB	AA	AB	AB	AA	AB	AA	AA
[illegible]																																						

snp_B1905	snp_B1910	snp_B1918	snp_B1921	snp_B1945	snp_B1954	snp_B1960	snp_B1963	snp_B1971	snp_B1993	snp_B2002	snp_B2080	snp_B2094	snp_B2114	snp_B2156	snp_B2158	snp_B2169	snp_B2175	snp_B2184	snp_B2188	snp_B2204	snp_B2215	snp_B2231	snp_B2236	snp_B2272	snp_B2288	snp_B2289	snp_B2299	snp_B2306	snp_B2307	snp_B2338	snp_B2352	snp_B2380	snp_B2395	snp_B2397	snp_B2422	snp_B2427	snp_B2440
10	10	10	10	10	10	10	10	10	10	10	10	10	10	10	10	10	10	10	10	10	10	10	10	10	10	10	10	10	10	10	10	10	10	10	10	10	10
--	--	--	--	--	--	LG4	--	--	--	--	--	--	--	--	--	--	--	--	--	LG4	--	--	LG4	--	--	--	--	--	--	--	--	--	--	--	--	--	--
AA	AB	AA	AA	AA	AB	AA	AA	AA	AA	AA	AB	AA	AB	AB	AA	AB	AB	AA	AA	AA	AA	AA	AA	AA	AA	AB	AA	AA	AA	AB	AB	AA	AB	AB	AB	AA	AB
[illegible]																																					

snp_B2457	snp_B2499	snp_B2503	snp_B2504	snp_B2518	snp_B2528	snp_B2532	snp_B2566	snp_B2589	snp_B2629	snp_B2671	snp_B2673	snp_B2676	snp_B2684	snp_B2710	snp_B2720	snp_B2723
10	10	10	10	10	10	10	10	10	10	10	10	10	10	10	10	10
--	--	--	--	--	--	--	--	LG4	--	LG4	--	LG4	--	LG4	--	--
AB	AA	AB	AA	AA	AB	AB	AB	AA	AA	AA	AB	AB	AA	AA	AA	AA
AA	AA	AB	AA	AB	AB	AA	AA	AA	AA	AA	AA	AA	AB	AA	AA	AA
AB	AB	AB	AB	AB	AB	AA	AA	AA	AB	AA	AB	AB	AB	AA	AA	AB
AB	AB	AA	AB	AA	AA	AB	AB	AB	AA	AB	AA	-	AB	AB	AB	AB
AB	AB	AB	AB	AA	AB	AB	AA	AB	AB	AB	AA	AB	AB	AB	AB	AB
AB	AA	AA	-	AB	AA	AB	-	AA	AA	AA	AA	-	AB	AB	-	AB
AA	AB	AA	AA	AB	AB	AB	AB	AA	AB	AB	AA	AB	AB	AB	AA	AA
AA	AA	AA	AA	AB	AA	AB	AA	AA	AB	AA	AB	AA	AA	AA	AA	AA
AA	AB	AB	AA	AB	AB	AB	AB	AA	AB	AA	AB	AA	AA	AA	AA	AB
AA	AA	AA	AB	AA	AA	AA	AB	AB	AA	AB	AB	AB	AA	AB	AA	AA
AB	AA	AB	AB	AB	AA	AB	AA	AA	AA	AA	AB	AA	AA	AA	AA	AA
AB	AB	AB	AB	AA	AB	AB	AB	AB	AB	AB	AA	AB	AB	AB	AA	AB
AB	AA	AB	AB	AB	AB	AA	AA	AA	AB	AA	AB	AB	AB	AB	AB	AB
AA	AA	AB	AB	AB	AA	AB	AA	AA	AB	AA	AB	AA	AA	AA	AA	AA
AB	AB	AA	AA	AB	AB	AB	AA	AA	AB	AB	AA	AB	AB	AB	AA	AA
AA	AB	AB	AA	AB	AB	AB	AB	AB	AA	AB	AB	AA	AB	AB	AA	AA
AB	AA	AB	AB	AB	AA	AB	AB	AA	AA	AA	AB	AA	AA	AA	AA	AB
AB	AB	AA	-	AA	AB	AB	-	AB	-	AB	AA	-	AA	AA	-	AA
AB	AB	AB	AB	AB	AB	AB	AB	AB	AB	AB	AA	AB	AB	AB	AB	AB
AA	AA	AB	AA	AB	AA	AB	AB	AA	AB	AA	AA	AB	-	AA	AB	AB
AB	AB	AB	AB	AB	AB	AB	AB	AB	AB	AB	AB	AB	AB	AB	AB	AB
AB	AB	AB	AA	AA	AB	AB	AB	AA	AA	AA	AA	AB	AB	AB	AB	AB
AB	AB	AA	-	AB	AB	-	AB	AB	-	AB	AA	-	-	AB	AB	AA
AB	AB	AB	AB	AA	AB	AB	AB	AB	AB	AB	AA	AB	AB	AB	AB	AA
AA	AB	AB	AB	AB	AA	AB	AA	AA	AB	AA	AB	AA	AA	AA	AA	AA
AB	AA	AB	AA	AA	AA	AA	AA	AA	AA	AA	AB	AA	AB	AA	AA	AB
AB	AB	AB	AB	AB	AB	AB	AA	AB	AB	AB	AB	AB	AB	AB	AB	AB
AA	AB	AB	AB	AA	AB	AB	AA	AB	AA	AB	AB	AB	AB	AB	AA	AB
AA	AA	AA	AB	AB	AB	AA	AA	AB	AB	AB	AA	-	AA	AB	AB	AB
AB	AB	AB	-	AA	AB	-	-	AA	AA	AA	-	-	-	AA	AB	AA
AA	AB	AA	AA	AA	AB	AA	AA	AB	AA	AB	AA	AB	AB	AB	AB	AA
AA	AB	AA	AA	AB	AB	AB	AB	AA	AB	AA	AA	AB	AB	AB	AB	AA
AA	AA	AA	-	AB	AA	AA	-	AA	-	AB	-	-	AA	AA	AA	AA
AB	AB	AB	-	AB	AB	AA	AA	AA	AA	AA	-	AA	AB	AA	AA	AA
AB	AA	AA	AB	AA	AA	AA	-	AA	AB	AB	AB	-	AA	AA	AB	AA
AB	AB	AB	AA	AB	AB	AB	AB	AB	AB	AB	AA	AB	AB	AB	AA	AB
AB	AA	AA	AB	AB	AA	AA	AB	AA	AB	AB	AA	AB	AB	AA	AB	AA
AB	AB	AA	AB	AA	AB	AA	AB	AA	AB	AB	AB	AA	AB	AB	AA	AA

snp_B4283	snp_B4290	snp_B4300	snp_B4310	snp_B4324	snp_B4325	snp_B4333	snp_B4341	snp_B4342	snp_B4346	snp_B4350	snp_B4360	snp_B4363	snp_B4395	snp_B4412	snp_B4472	snp_B4490	snp_B4545	snp_B4566	snp_B4569
10	10	10	10	10	10	10	10	10	10	10	10	10	10	10	10	10	10	10	10
--	--	--	--	--	--	--	--	--	--	--	--	LG4	--	--	--	--	--	--	--
AB	AA	AB	AA	AB	AA	AA	AA	AB	AB	AA	AA	AA	AB	AA	AA	AB	AB	AA	AA
AA	AB	AA	AA	AA	AA	AA	AA	AA	AA	AB	AA	AA	AA	AA	AA	AA	AA	AA	AA
AB	AB	AA	AB	AB	AA	AB	AB	AA	AB	AB	AA	AB	AB	AB	AB	AB	AA	AB	AA
-	AA	AA	AB	AA	AB	AA	AB	AB	AA	AA	AB	AB	AB	AA	AB	AA	AB	AA	AB
AB	AB	AA	AB	AA	AB	AA	AB	AB	AB	AB	AB	AB	AB	AA	AB	AA	AB	AA	AB
-	AA	AA	-	AB	AA	AA	AA	AA	AA	AA	-	AA	AB	-	AB	AA	AB	-	AB
AB	AA	AA	AA	AA	AB	AA	AB	AB	AB	AB	AA	AB	AA	AB	AA	AA	AA	AB	AA
AB	AA	AB	AA	AB	AA	AB	AA	AA	AB	AB	AA	AA	AA	AB	AA	AB	AA	AB	AA
AA	AB	AA	AB	AB	AB	AB	AB	AB	AB	AA	AA	AA	AA	AB	AA	AB	AB	AB	AA
AB	AA	AB	AA	AA	AB	AB	AA	AB	AB	AB	AA	AB	AA	AA	AA	AB	AA	AB	AA
AB	AA	AB	AB	AA	AA	AA	AA	AB	AB	AA	AA	AA	AB	AB	AA	AB	AA	AB	AA
AB	AB	AB	AB	AA	AA	AB	AA	AA	AB	AB	AB	AB	AB	AA	AB	AB	AB	AB	AB
AB	AB	AB	AB	AB	AB	AB	AB	AA	AB	AB	AB	AB	AA	AB	AB	AB	AB	AB	AB
AB	AA	AB	AB	AB	AA	AB	AA	AA	AB	AA	AA	AA	AA	AB	AA	AB	AA	AB	AB
AB	AB	AA	AA	AA	AA	AB	AB	AA	AB	AA	AB	AB	AB	AA	AB	AB	AB	AB	AB
AA	AB	AA	AA	AA	AB	AA	AB	AA	AA	AA	AB	AA	AB	AB	AA	AA	AA	AA	AA
AB	AB	AA	AB	AB	AB	AB	AA	AB	AB	AA	AB	AA	AB	AB	AB	AA	AB	AB	AA
-	AA	AA	-	AB	-	AA	AA	AB	AA	AB	-	AA	-	-	AB	AA	AB	-	AB
AB	AB	AB	AB	AB	AB	AB	AB	AB	AB	AB	AB	AB	AB	AB	AB	AB	AB	AB	AB
AA	AB	AA	-	AA	AB	AA	AA	AB	AA	-	AA	AA	AB	AB	AB	AA	AA	AA	AA
AB	AB	AB	AB	AB	AB	AB	AB	AB	AB	AB	AB	AA	AB	AB	AB	AA	AB	AB	AA
AA	AB	AA	AB	AB	AA	AB	AB	AA	AB	AA	AB	AB	AB	AA	AB	AB	AB	AA	AB
AA	AB	AA	-	AB	-	AA	AB	AA	AA	-	AA	AA	AB	-	AA	AA	AA	AA	-
AB	AB	AB	AB	AB	AB	AB	AB	AB	AB	AA	AB	AB	AB	AA	AB	AB	AB	AB	AB
AB	AB	AB	AA	AB	AA	AB	AB	AB	AB	AB	AA	AB	AA	AB	AA	AB	AA	AB	AA
AB	AB	AB	AA	AB	AA	AB	AB	AB	AB	AA	AA	AA	AA	AB	AA	AB	AA	AB	AA
AB	AB	AB	AB	AB	AB	AB	AB	AB	AB	AB	AB	AB	AB	AB	AB	AB	AB	AB	AB
AB	AB	AB	AA	AB	AA	AB	AB	AA	AB	AA	AB	AB	AB	AA	AB	AB	AA	AB	AA
AA	AB	AA	AA	AB	AA	AA	AB	AB	AA	AB	AB	AB	AA	AB	AB	AB	AB	AA	-
-	AB	AA	AB	AB	AA	-	AB	AB	AA	-	-	AA	AA	-	AA	AB	AB	AA	AA
AA	AA	AA	AA	AA	AA	AA	AB	AA	AA	AB	AA	AB	AA	AA	AA	AA	AA	AA	AA
AA	AA	AA	AA	AA	AB	AA	AA	AA	AA	AA	AA	AB	AA	AB	AA	AA	AA	AA	AA
AB	AA	AB	AA	AA	AB	AB	AA	AB	AB	-	-	AB	AA	AB	AA	AA	AA	AB	AA
AB	AA	AA	AB	AA	AB	AB	AB	AB	AB	AA	-	AA	AB	-	AA	AB	AB	-	AA
AB	AA	AB	-	AB	AA	AB	AA	AB	AB	AA	-	AA	AA	-	AB	AB	AB	-	AB
AA	AB	AB	AB	AA	AB	-	AB	AA	AB	AB	AB	AB	AB	AA	AB	AB	AB	AA	AB
AB	AB	AB	AB	AB	AB	AB	AA	AB	AB	AB	AB	AB	AA	AB	AB	AB	AB	AB	AB
AB	AA	AA	AB	AB	AA	AB	AB	AB	AB	AB	AB	AB	AB	AA	AB	AB	AB	AB	AB
AA	AA	AA	AA	AA	AA	AA	AA	AA	AA	AA	AA	AA	AA	AA	AA	AA	AA	AA	AB
AB	AA	AA	AB	AA	AA	AA	AB	AB	AA	-	-	AA	AB	AA	AB	AA	AB	-	AB
AA	AB	AA	AB	AA	AA	AA	AB	AB	AA	AA	AB	AA	AB	AB	AB	AA	AB	AA	AB
AA	AA	AA	AA	AA	AB	AA	AA	AA	AA	AA	AA	AA	AB	AB	AA	AA	AA	AA	AA
AA	AA	AA	AA	AA	AB	AA	AA	AA	AA	-	AA	AA	AA	AB	AA	AA	AA	AA	AA
AB	AA	AA	AA	AA	AB	AB	AB	AA	AB	AA	AA	AA	AA	AA	AA	AA	AA	AB	AA
AA	AB	AA	AA	AA	AB	AA	AB	AB	AB	AA	AB	AB	AA	AB	AB	AA	AB	AA	AB
AB	AB	AB	AB	AB	AA	AB	AA	AB	AB	AA	AB	AA	AA	AA	AB	AB	AB	AB	AB
AB	AB	AB	AB	AB	AB	AB	AB	AB	AB	AB	AB	AB	AB	AA	AB	AB	AB	AB	AB
AB	AA	AB	AB	AB	AA	AB	AA	AB	AB	AA	AB	AB	AB	AA	AB	AB	AB	AB	AB
AA	AA	AA	AA	AA	AB	AB	AA	AA	AA	AB	AA	AA	AB	AB	AA	AA	AA	AA	AA
AA	AB	AA	AB	AA	AB	AA	AB	AB	AA	AB	AB	AB	AB	AA	AB	AA	AA	AA	AB
AB	AB	AB	AA	AB	AB	AA	AB	AB	AB	AB	AA	AB	AB	AB	AB	AA	AB	AB	AA
AA	AA	AB	AB	AB	AA	AA	AA	AA	AA	-	-	AA	AA	AA	AB	AA	AB	AA	AB
AB	AA	AA	AA	AB	AB	AB	AA	AB	AB	AA	AA	AA	AB	AB	AA	AA	AB	AB	AA
AB	AB	AB	AB	AB	AB	AB	AB	AB	AB	AB	AB	AB	AB	AB	AB	AB	AB	AB	AB
[illegible]																			

[illegible]

snp_B5665	snp_B5670	snp_B5672	snp_B5675	snp_B5719	snp_B5723	snp_B5758	snp_B5783	snp_B5848	snp_B5856	snp_B5863	snp_B5888	snp_B5897	snp_B5899	snp_B5903	snp_B5917	snp_B5923	snp_B5938	snp_B5944	snp_B5947	snp_B5971	snp_B5989	snp_B5996	snp_B6003	snp_B6017	snp_B6038	snp_B6135	snp_B6192	snp_B6219	snp_B0064	snp_B0082	snp_B0088	snp_B0111	snp_B0122	snp_B0159	snp_B0179	snp_B0200	snp_B0231	snp_B0232	snp_B0234	snp_B0247	snp_B0264	snp_B0275	snp_B0288	snp_B0331	snp_B0436	snp_B0550	snp_B0609	snp_B0614	snp_B0621	snp_B0626	snp_B0629	snp_B0665	snp_B0670	snp_B0676	snp_B0693	snp_B0725	snp_B0738	snp_B0822	snp_B0827	snp_B0871	snp_B0886	snp_B0939	snp_B0951	snp_B1002	snp_B1084	snp_B1112	snp_B1115	snp_B1125	snp_B1131	snp_B1146	snp_B1174	snp_B1176	snp_B1214	snp_B1279	snp_B1284	snp_B1287
10	10	10	10	10	10	10	10	10	10	10	10	10	10	10	10	10	10	10	10	10	10	10	10	10	10	10	10	10	11	11	11	11	11	11	11	11	11	11	11	11	11	11	11	11	11	11	11	11	11	11	11	11	11	11	11	11	11	11	11	11	11	11	11	11	11	11	11	11	11	11	11	11	11	11	11	11
--	--	--	--	--	--	--	--	--	--	--	--	--	--	--	LG4	--	--	--	LG4	--	--	--	--	--	--	LG4	--	--	--	--	LG11	--	LG12	--	LG11	--	--	--	--	LG11	--	--	--	--	--	--	--	--	LG11	LG11	--	--	--	LG11	--	LG12	--	--	--	--	--	--	--	--	--	--	--	--	--	--	LG12	--	--	--	--	--
AB	AA	AB	AB	AA	AA	AB	AA	AA	AA	AA	AA	AA	AA	AB	AB	AA	AA	AB	AA	AB	AB	AA	AB	AB	AA	AA	AB	AA	AA	AB	AB	AA	AA	AB	AA	AA	AA	AA	AA	AA	AA	AA	AA	AA	AA	AB	AB	AB	AA	AA	AA	AA	AB	AB	AA	AA	AB	AB	AB	AA	AA	AA	AA	AA	AB	AB	AB	AB	AB	AA	AA	AA	AA	AB	AA	AA
AB	AA	AA	AB	AA	AA	AA	AA	AA	AA	AB	AA	AA	AA	AA	AA	AA	AA	AA	AA	AB	AB	AA	AB	AB	AA	AA	AB	AA	AB	AA	AB	AB	AB	AB	AB	AA	AB	AB	AA	AA	AB	AB	AB	AB	AA	AB	AB	AB	AA	AA	AB	AA	AB	AB	AB	AB	AB	AA	AA	AB	AB	AA	AA	AA	AB	AB	AB	AA	AB	AA	AB	AB	AB	AB	AB	AA
AB	AA	AA	AB	AB	AA	AA	AA	AB	AA	AA	AB	AA	AB	AA	AB	AB	AB	AA	AA	AB	AB	AA	AB	AA	AA	AA	AA	AA	AB	AA	AB	AB	AB	AB	AA	AA	AB	AB	AA	AB	AB	AB	AB	AB	AA	AB	AA	AA	AA	AB	AA	AB	AB	AB	AB	AB	AB	AA	AB	AA	AA	AB	AB	AB	AB	AB	AB	AA	AB	AA	AB	AA	AB	AB	AB	AA
AA	AA	AB	-	AA	AA	AB	AB	-	AA	AA	AB	AB	-	AB	AA	AB	AB	AB	AB	AB	AA	AB	AA	AA	-	AB	AA	-	AB	AB	AB	AA	AA	AB	AB	AB	-	AB	AB	AB	AA	AB	AA	AB	AB	AB	AB	AB	AB	AB	-	AB	AB	AB	-	AA	AB	AB	AA	AB	AB	-	AB	AB	AB	AB	AB	AB	AB	-	AB	-	AB	AB	AA	AB
AA	AB	AB	AB	AB	AB	AB	AB	AB	AB	AA	AA	AA	AA	AA	AB	AB	AB	AA	AB	AB	AB	AB	AA	AA	AA	AB	AB	-	AB	AB	AA	AB	AB	AB	AB	AB	AB	AB	AB	AB	AB	AB	AB	AB	AB	AB	AB	AB	AB	AB	AB	AB	AA	AA	AB	AB	AB	AB	AB	AB	AA	AB	AB	AB	AA	AB	AB	AB	AB	AB	AB	AB	AB	AB	AB	AB
-	AB	-	-	AA	AA	AB	AA	-	AB	-	AA	AA	-	AA	AA	AB	AB	-	-	AB	AA	AB	-	AB	-	-	AA	-	-	AB	-	-	AA	AA	AB	AA	-	-	-	-	AA	AB	AA	AA	AB	AA	AB	AB	AB	-	-	AB	AB	AB	-	AA	-	-	AB	AB	-	-	AA	AB	AB	-	AA	-	-	-	-	-	AA	AA	-	AA

snp_B1298	snp_B1361	snp_B1372	snp_B1379	snp_B1381	snp_B1450	snp_B1534	snp_B1566	snp_B1588	snp_B1617	snp_B1662	snp_B1697	snp_B1703	snp_B1715	snp_B1730	snp_B1748	snp_B1764	snp_B1796	snp_B1802	snp_B1813	snp_B1858	snp_B1888	snp_B1915	snp_B1925
11	11	11	11	11	11	11	11	11	11	11	11	11	11	11	11	11	11	11	11	11	11	11	11
--	--	--	--	--	--	--	--	LG11	--	--	--	--	LG11	LG11	--	LG12	--	--	--	LG11	--	--	--
AA	AA	AA	AA	AB	AB	AB	AB	AA	AA	AA	AB	AA	AA	AA	AA	AA	AB	AB	AA	AB	AB	AB	AA
AB	AA	AA	AA	AB	AB	AA	AB	AA	AA	AA	AA	AB	AA	AA	AA	AA	AB	AB	AA	AB	AA	AB	AA
AB	AB	AA	AB	AA	AB	AA	AB	AA	AA	AA	AB	AB	AB	AB	AA	AB	AA	AA	AB	AB	AB	AA	AA
AB	AB	AB	AB	AB	AB	AA	AB	AB	AB	AB	AB	AA	AB	AB	AB	AB	-	AB	AB	AB	AA	AB	AB
AA	AB	AB	AB	AB	AB	AA	AB	AB	AB	AB	AA	AB	AB	AB	AB	AB	AB	AA	AB	AB	AB	AB	AB
-	AB	AB	AA	-	-	AB	AB	AB	AB	AA	AB	AA	AB	-	AB	-	AA	AB	AA	-	-	AB	-

snp_B1940	snp_B1947	snp_B1972	snp_B2030	snp_B2161	snp_B2179	snp_B2194	snp_B2223	snp_B2264	snp_B2291	snp_B2319	snp_B2320	snp_B2324	snp_B2343	snp_B2374	snp_B2376	snp_B2420	snp_B2421	snp_B2432	snp_B2462	snp_B2468	snp_B2473	snp_B2507	snp_B2551	snp_B2616	snp_B2646	snp_B2647	snp_B2651	snp_B2656	snp_B2666
11	11	11	11	11	11	11	11	11	11	11	11	11	11	11	11	11	11	11	11	11	11	11	11	11	11	11	11	11	11
--	LG11	--	--	LG11	--	--	--	LG12	--	--	--	--	--	--	--	--	--	--	LG12	--	--	--	--	--	--	--	--	--	LG11
AA	AB	AA	AA	AA	AB	AA	AA	AA	AA	AB	AA	AA	AA	AA	AA	AA	AA	AA	AA	AB	AB	AB	AA	AB	AA	AA	AA	AB	AB
AB	AB	AA	AA	AA	AB	AA	AA	AB	AB	AA	AA	AA	AA	AA	AB	AA	AB	AA	AB	AB	AA	AA	AA	AA	AA	AA	AA	AA	AB
AA	AB	AB	AA	AB	AA	AB	AA	AB	AB	AB	AA	AB	AA	AA	AB	AB	AB	AA	AB	AB	AB	AB	AA	AA	AB	AA	AB	AA	AB
AB	AB	AB	-	AA	AB	AB	AA	AA	AB	AA	AB	AB	AB	AA	AA	AA	AA	AA	AB	AB	-	AB	AA	AA	AB	AB	AB	AA	AB
AA	AB	AB	AB	AA	AA	AB	AB	AB	AB	AB	AB	AB	AA	AB	AB	AB	AB	AB	AA	AB	AB	AB	AB	AB	AB	AB	AB	AB	AA
AB	AB	AB	AB	AB	AB	AB	AB	-	AB	-	AA	AB	AB	AB	-	AA	AA	AB	AA	AB	AB	-	-	-	AB	-	AB	AB	AB

snp_B2711	snp_B2722	snp_B2738	snp_B2745	snp_B2752	snp_B2762	snp_B2771	snp_B2787	snp_B2805	snp_B2852	snp_B2873	snp_B2941	snp_B2949	snp_B2970	snp_B2980	snp_B2988	snp_B3007	snp_B3013	snp_B3051	snp_B3059	snp_B3077	snp_B3121	snp_B3134
11	11	11	11	11	11	11	11	11	11	11	11	11	11	11	11	11	11	11	11	11	11	11
--	--	--	--	--	--	--	--	--	--	--	--	--	--	--	--	--	LG12	--	--	--	--	--
AA	AB	AB	AB	AA	AA	AA	AB	AB	AB	AA	AB	AA	AA	AB	AB	AA	AA	AB	AA	AB	AB	AB
AA	AB	AB	AA	AB	AB	AA	AA	AB	AB	AA	AB	AA	AB	AB	AB	AA	AB	AB	AA	AB	AA	AB
AB	AB	AB	AB	AA	AB	AA	AB	AB	AA	AB	AA	AB	AB	AA	AB	AB	AB	AA	AA	AA	AB	AA
AB	AB	AB	AB	AA	AB	AA	AB	AB	AB	AA	AB	AB	AB	AB	AB	AB	AA	AB	-	-	AB	AB
AB	AA	AB	AA	AB	AB	AB	AB	AB	AB	AB	AB	AB	AB	AB	AB	AB	AB	AB	AB	AB	AB	AB
AB	AB	AA	AB	-	AA	AB	-	AA	AA	AA	-	AB	AA	AA	AA	AA	-	AA	-	-	-	AA

snp_B3160	snp_B3184	snp_B3195	snp_B3237	snp_B3256	snp_B3277	snp_B3296	snp_B3334	snp_B3366	snp_B3386	snp_B3415	snp_B3448	snp_B3483	snp_B3497	snp_B3509	snp_B3513	snp_B3602	snp_B3604	snp_B3605	snp_B3606	snp_B3624	snp_B3644	snp_B3646	snp_B3675	snp_B3712	snp_B3718
11	11	11	11	11	11	11	11	11	11	11	11	11	11	11	11	11	11	11	11	11	11	11	11	11	11
--	--	--	--	--	LG12	LG11	--	--	--	LG11	--	--	--	--	--	--	--	--	--	--	--	--	--	--	LG12
AA	AB	AA	AB	AB	AA	AA	AA	AA	AA	AB	AB	AB	AA	AA	AB	AB	AB	AB	AB	AA	AB	AB	AA	AB	AA

snp_B3720	snp_B3728	snp_B3733	snp_B3839	snp_B3901	snp_B3923	snp_B3960	snp_B4004	snp_B4012	snp_B4049	snp_B4057	snp_B4065	snp_B4124	snp_B4137	snp_B4200	snp_B4220	snp_B4252	snp_B4260	snp_B4274	snp_B4291	snp_B4317	snp_B4320	snp_B4355	snp_B4359	snp_B4394	snp_B4423
11	11	11	11	11	11	11	11	11	11	11	11	11	11	11	11	11	11	11	11	11	11	11	11	11	11
--	--	--	--	--	--	--	--	--	--	--	--	--	--	--	--	--	--	--	--	--	LG11	LG12	--	--	LG12
AB	AA	AA	AB	AB	AA	AB	AA	AB	AA	AA	AA	AA	AB	AA	AB	AB	AB	AB	AA	AA	AA	AA	AA	AB	AA

snp_B4441	snp_B4461	snp_B4542	snp_B4552	snp_B4641	snp_B4684	snp_B4687	snp_B4709	snp_B4721	snp_B4740	snp_B4767	snp_B4784	snp_B4804	snp_B4922	snp_B4936	snp_B4945	snp_B4950	snp_B4963	snp_B5034	snp_B5070	snp_B5074	snp_B5094	snp_B5105	snp_B5113	snp_B5128
11	11	11	11	11	11	11	11	11	11	11	11	11	11	11	11	11	11	11	11	11	11	11	11	11
--	--	--	--	--	--	--	--	--	LG11	--	LG12	--	--	--	--	--	--	LG11	--	--	LG12	--	--	--
AB	AA	AA	AB	AA	AA	AA	AB	AA	AB	AB	AA	AA	AB	AB	AB	AB	AA	AA	AB	AA	AA	AA	AB	AA

snp_B5149	snp_B5158	snp_B5203	snp_B5241	snp_B5307	snp_B5311	snp_B5365	snp_B5376	snp_B5378	snp_B5383	snp_B5387	snp_B5429	snp_B5447	snp_B5516	snp_B5532	snp_B5566	snp_B5621
11	11	11	11	11	11	11	11	11	11	11	11	11	11	11	11	11
--	--	--	--	--	LG11	LG11	--	--	--	--	--	LG12	--	--	LG11	--
AA	AB	AB	AA	AB	AA	AB	AB	AB	AB	-	AA	AA	AA	AA	AA	AA
AB	AA	AB	AB	AA	AA	AB	AB	AB	AA	AB	AB	AB	AA	AA	AA	AA
AB	AA	AB	AB	AB	AA	AB	AB	AA	AA	AB	AA	AB	AB	AA	AA	AA
AB	AB	-	AB	AB	AA	AB	-	AB	AB	AB	AB	AA	-	AB	AB	AB
AA	AB	AB	AB	AB	AA	AB	AB	AB	AB	AB	AA	AB	AB	AB	AB	AB
AB	-	-	-	AA	AA	AB	AA	AA	AB	AA	AB	AA	AB	AB	-	-
AB	AB	AB	AA	AB	AA	AB	AA	AB	AA	AB	AA	AB	AA	AB	AA	AA
AA	AB	AB	AA	AB	AB	AA	AA	AA	AA	AB	AA	AB	AA	AA	AB	AB
AB	AA	AB	AB	AB	AB	AA	AA	AA	AA	AB	AB	AB	AA	AA	AB	AA
AB	AA	AA	AB	AA	AB	AA	AB	AA	AB	AA	AB	AA	AA	AB	AB	AA
AB	AA	AB	AA	AA	AB	AA	AA	AB	AA	AB	AB	AA	AB	AA	AB	AA
AA	AB	AA	AB	AB	AB	AA	AB	AA	AB	AA	AA	AB	AB	AB	AB	AB
AB	AB	AB	AA	AB	AB	AB	AB	AB	AA	AB	AB	AB	AA	AA	AB	AB
AB	AA	AB	AA	AB	AB	AB	AB	AB	AA	AB	AB	AB	AB	AA	AB	AA
AB	AA	AB	AA	AB	AA	AA	AA	AB	AA	AB	AB	AA	AB	AA	AB	AA
AA	AA	AA	AA	AA	AB	AA	AB	AB	AB	AA	AA	AA	AB	AB	AA	AA
AB	AB	AA	AA	AB	AA	AB	AA	AA	AB	AB	AB	AA	AB	AB	AB	AB
AB	-	-	AA	AA	AA	AA	-	AB	-	AA	AA	-	AB	AA	-	-
AA	AB	AB	AB	AB	AA	AA	AB	AB	AA	AB	AA	AB	AA	AA	AA	AA
AA	AB	AB	AB	AB	AB	AA	AA	AB	AA	AB	AB	AB	AA	AA	AB	AB
AB	AA	AA	AB	AA	AB	AB	AA	AB	AB	AA	AB	AA	AB	AB	AB	AB
AA	AB	AB	AB	AB	AA	AA	AA	AA	AA	AB	AA	AB	AA	AA	AA	AA
AA	-	-	-	-	-	AB	-	AA	AA	AA	AB	AA	-	AA	AA	-
AA	AB	AB	AA	AB	AA	AA	AA	AB	AA	AB	AA	AB	AB	AB	AA	AA
AA	AA	AA	AB	AA	AA	AA	AA	AA	AA	AB	AB	AA	AA	AA	AA	AA
AB	AA	AA	AA	AB	AA	AB	AB	AB	AB	AA	AB	AA	AA	AB	AA	AB
AB	AB	AB	AB	AB	AA	AB	AB	AB	AB	AB	AB	AB	AB	AB	AB	AB
AB	AB	AB	AB	AB	AB	AB	AB	AB	AB	AB	AB	AB	AB	AB	AB	AB
AB	AA	AA	AB	AA	AB	AA	AA	AB	AB	-	AA	AA	-	AB	AB	AA
AA	AA	-	-	AA	AB	AA	-	AB	AA	-	AA	-	-	AA	-	-
AB	AB	AB	AB	AB	AB	AB	AB	AB	AB	AB	AB	AB	AB	AB	AB	AB
AB	AA	AA	AA	AA	AA	AB	AB	AA	AB	AA	AA	AA	AA	AB	AA	AA
AA	AB	AB	AA	-	AA	AA	AA	AB	AB	AA	AA	AB	AA	AA	-	AA
AA	AA	AA	AA	AA	AB	AA	-	AB	AB	-	AA	AA	AB	AB	AB	-
AB	AA	-	AA	AA	-	AB	-	AB	AB	-	AB	-	AB	AB	-	AB
AA	AA	-	AA	AA	AA	AA	-	AA	AA	AA	AA	AA	AA	AA	AA	-
AB	AA	AA	AA	AA	AB	AB	AA	AA	AB	AA	AB	AA	AB	AB	AB	AB
AA	AB	AB	AA	AA	AB	AA	AA	AB	AA	AA	AA	AB	-	AA	AA	AA
AB	AB	AB	AB	AB	AB	AB	AB	AB	AB	AB	AB	AB	AB	AB	AB	AB
AB	-	AB	-	AB	AB	AA	AB	AB	AA	AB	AB	AB	AB	AA	-	-
AA	AB	AB	AB	AA	AB	AA	AA	AB	AA	AB	AA	AB	AA	AA	AA	AA
AA	AB	AB	AB	AB	AB	AA	AB	AB	AA	AB	AB	AB	AA	AA	AA	AA
AA	AB	AA	AA	AB	AB	AA	AB	AB	AA	AA	AB	AA	AB	AA	AB	AB

snp_B5436	snp_B5417	snp_B5411	snp_B5399	snp_B5394	snp_B5392	snp_B5374	snp_B5334	snp_B5305	snp_B5274	snp_B5273	snp_B5264	snp_B5250	snp_B5217	snp_B5192	snp_B5164	snp_B5151	snp_B5106	snp_B5084	snp_B5024	snp_B5021	snp_B4983	snp_B4965	snp_B4937	snp_B4907	snp_B4901	snp_B4860	snp_B4850	snp_B4797	snp_B4759	snp_B4725	snp_B4645	snp_B4637	snp_B4635	snp_B4593	snp_B4586	snp_B4572	snp_B4460	snp_B4398	snp_B4390	snp_B4388	snp_B4352	snp_B4339	snp_B4328	snp_B4309	snp_B4280	snp_B4237	snp_B4225	snp_B4212	snp_B4196	snp_B4188	snp_B4089	snp_B4023	snp_B3990	snp_B3987	snp_B3957	snp_B3956	snp_B3950	snp_B3921	snp_B3914	snp_B3906	snp_B3899	snp_B3894	snp_B3879	snp_B3828	snp_B3826	snp_B3794	snp_B3772	snp_B3742	snp_B3735	snp_B3722	snp_B3695	snp_B3690	snp_B3679	snp_B3672	snp_B3620	snp_B3579
12	12	12	12	12	12	12	12	12	12	12	12	12	12	12	12	12	12	12	12	12	12	12	12	12	12	12	12	12	12	12	12	12	12	12	12	12	12	12	12	12	12	12	12	12	12	12	12	12	12	12	12	12	12	12	12	12	12	12	12	12	12	12	12	12	12	12	12	12	12	12	12	12	12	12	12	12
--	--	--	--	--	--	--	--	--	--	LG7	--	--	--	--	--	--	--	--	--	--	--	--	--	--	--	--	LG7	--	--	--	LG7	--	--	--	--	--	--	--	LG7	--	--	--	--	--	--	--	--	--	--	LG7	LG7	--	--	--	--	--	--	--	LG7	--	--	--	--	--	--	--	--	--	--	LG7	--	--	--	--	--	--
[illegible]	[illegible]	[illegible]	[illegible]	[illegible]	[illegible]	[illegible]	[illegible]	[illegible]	[illegible]	[illegible]	[illegible]	[illegible]	[illegible]	[illegible]	[illegible]	[illegible]	[illegible]	[illegible]	[illegible]	[illegible]	[illegible]	[illegible]	[illegible]	[illegible]	[illegible]	[illegible]	[illegible]	[illegible]	[illegible]	[illegible]	[illegible]	[illegible]	[illegible]	[illegible]	[illegible]	[illegible]	[illegible]	[illegible]	[illegible]	[illegible]	[illegible]	[illegible]	[illegible]	[illegible]	[illegible]	[illegible]	[illegible]	[illegible]	[illegible]	[illegible]	[illegible]	[illegible]	[illegible]	[illegible]	[illegible]	[illegible]	[illegible]	[illegible]	[illegible]	[illegible]	[illegible]	[illegible]	[illegible]	[illegible]	[illegible]	[illegible]	[illegible]	[illegible]	[illegible]	[illegible]	[illegible]	[illegible]	[illegible]	[illegible]	[illegible]	[illegible]

snp_B3577	snp_B3553	snp_B3540	snp_B3475	snp_B3392	snp_B3385	snp_B3352	snp_B3295	snp_B3291	snp_B3278	snp_B3255	snp_B3245	snp_B3228	snp_B3130	snp_B3120	snp_B3117	snp_B3106	snp_B3069	snp_B3038	snp_B3021	snp_B2946	snp_B2945	snp_B2944	snp_B2892	snp_B2859	snp_B2856	snp_B2821	snp_B2799	snp_B2795	snp_B2783	snp_B2779	snp_B2774	snp_B2750	snp_B2628	snp_B2581	snp_B2568	snp_B2481	snp_B2451	snp_B2448	snp_B2439	snp_B2378	snp_B2360	snp_B2344	snp_B2309	snp_B2268	snp_B2198	snp_B2191	snp_B2170	snp_B2157	snp_B2129	snp_B2127	snp_B2126	snp_B2122	snp_B2088	snp_B2064	snp_B2051	snp_B2012	snp_B1978	snp_B1976	snp_B1876	snp_B1851	snp_B1825	snp_B1805	snp_B1792	snp_B1783	snp_B1757	snp_B1734	snp_B1708	snp_B1663	snp_B1661	snp_B1649	snp_B1644	snp_B1631	snp_B1585	snp_B1575	snp_B1565	snp_B1389
12	12	12	12	12	12	12	12	12	12	12	12	12	12	12	12	12	12	12	12	12	12	12	12	12	12	12	12	12	12	12	12	12	12	12	12	12	12	12	12	12	12	12	12	12	12	12	12	12	12	12	12	12	12	12	12	12	12	12	12	12	12	12	12	12	12	12	12	12	12	12	12	12	12	12	12	12
--	LG7	--	LG7	--	--	LG7	--	--	--	LG7	--	--	--	--	--	--	LG7	--	--	--	--	--	--	--	LG7	--	--	--	--	--	--	--	--	--	--	--	--	--	--	--	--	--	--	--	--	--	--	--	--	--	--	--	--	--	--	--	--	--	--	--	--	--	--	--	--	--	--	--	--	--	--	--	LG7	--	LG7	--
[illegible]																																																																												

snp_B1350	snp_B1348	snp_B1303	snp_B1261	snp_B1237	snp_B1126	snp_B1124	snp_B1100	snp_B1041	snp_B1014	snp_B1011	snp_B0996	snp_B0957	snp_B0950	snp_B0948	snp_B0943	snp_B0942	snp_B0877	snp_B0868	snp_B0811	snp_B0794	snp_B0778	snp_B0711	snp_B0689	snp_B0638	snp_B0538	snp_B0434	snp_B0419	snp_B0364	snp_B0354	snp_B0340	snp_B0327	snp_B0325	snp_B0317	snp_B0291	snp_B0281	snp_B0271	snp_B0239	snp_B0225	snp_B0212	snp_B0199	snp_B0176	snp_B0127	snp_B0113	snp_B0098	snp_B0078	snp_B0075
12	12	12	12	12	12	12	12	12	12	12	12	12	12	12	12	12	12	12	12	12	12	12	12	12	12	12	12	12	12	12	12	12	12	12	12	12	12	12	12	12	12	12	12	12	12	12
--	LG7	--	--	--	--	--	--	--	--	LG7	--	--	--	--	--	--	--	--	--	LG7	--	--	--	--	--	--	--	--	--	--	--	--	--	--	--	--	--	--	--	LG7	--	--	--	--	--	--
AB	AA	AA	AB	AA	AA	AB	AA	AA	AA	AA	AA	AB	AA	AA	AA	AA	AB	AB	AB	AA	AA	AB	AA	AA	AA	AA	AA	AA	AA	AA	AA	AB	AA	AB	AA	AA	AA	AA	AB	AA	AB	AB	AB	AB	AB	AB
AA	AB	AA	AA	AB	AB	AB	AA	AB	AA	AA	AA	AA	AB	AA	AA	AB	AB	AA	AA	AA	AB	AB	AB	AA	AB	AA	AA	AA	AB	AB	AA	AB	AB	AB	AB	AB	AA	AB	AA	AB	AA	AB	AB	AB	AB	AB
AB	AB	AB	AB	AA	AA	AB	AA	AA	AA	AA	AA	AB	AA	AB	AA	AA	AB	AA	AB	AB	AB	AB	AA	AB	AA	AA	AA	AB	AB	AA	AA	AA	AA	AB	AA	AB	AA	AB	AA	AA	AB	AB	AA	AB	AB	AA
AA	AB	AA	AA	AA	AA	AB	AB	AB	AB	AB	AA	AA	AB	AB	-	AB	-	AA	AB	-	AA	AA	AB	AB	AA	AB	AA	AB	AA	AA	AB	AB	-	AA	AA	AB	AA	AA	AA	AB	AA	AA	AA	AA	AA	AA
AA	AB	AB	AA	AB	AB	AB	AB	AB	AA	AB	AA	AA	AA	AB	AB	AA	AA	AB	AA	AB	AB	AB	AB	AA	AA	AB	AA	AA	AA	AB	AB	AB	AA	AA	AB	AB	AB	AB	AB	AB	AA	AA	AA	AB	AA	AB
AB	-	AA	-	AB	AB	AB	AB	AB	AA	AB	-	AA	AA	-	AB	-	-	AB	-	-	AB	AB	-	-	-	-	AB	-	-	AA	-	AA	-	AA	-	-	AA	AA	AB	AB	AA	AA	-	-	-	-
AA	AB	AA	AB	AB	AA	AB	AB	AB	AB	AB	AB	AB	AB	AB	AB	AB	AB	AB	AA	AB	AA	AB	AA	AA	AB	AB	AA	AB	AB	AB	AB	AB	AB	AB	AB	AB	AB	AB	AB	AB	AB	AB	AB	AA	AB	AB
AB	AA	AB	AB	AA	AA	AA	AA	AA	AA	AA	AB	AB	AA	AB	AA	AA	AB	AB	AB	AA	AB	AB	AA	AB	AA	AB	AB	AA	AA	AB	AA	AA	AA	AB	AB	AA	AB	AB	AB	AA	AB	AB	AB	AA	AB	AB
AB	AB	AA	AA	AB	AB	AB	AA	AB	AB	AB	AA	AA	AB	AB	AA	AB	AB	AB	AB	AB	AB	AB	AB	AB	AB	AB	AB	AA	AB	AA	AB	AA	AB	AB	AA	AB	AB	AB	AA	AB	AB	AA	AA	AB	AB	AA
