# Supplementary material for: A Chromosome-Level Genome Assembly of the European Beech (Fagus sylvatica) Reveals Anomalies for Organelle DNA Integration, Repeat Content and Distribution of SNPs
Source: Front Genet. 2022 Feb 8;12:691058. doi: 10.3389/fgene.2021.691058 (PMC8862710; doi:10.3389/fgene.2021.691058)
Supplement: Supplementary file 1 [file DataSheet1.PDF]

Supplementary File 1. Details of annotated repeat elements in *Fagus sylvatica*.

Repeat elements	# of elements	size	percentage of genome
Retroelements	87628	81859802 bp	15.15 %
LINEs:	36016	19755558 bp	3.66 %
RTE/Bov-B	5707	963701 bp	0.18 %
L1/CIN4	30309	18791857 bp	3.48 %
LTR elements:	51612	62104244 bp	11.49 %
Ty1/Copia	26740	25500497 bp	4.72 %
Gypsy/DIRS1	22010	34391985 bp	6.36 %
DNA transposons	27884	14339425 bp	2.65 %
hobo-Activator	14367	4870881 bp	0.90 %
Tourist/Harbinger	4205	1873197 bp	0.35 %
Rolling-circles	7484	3397980 bp	0.63 %

Supplementary File 2. Number of PLAZA genes shared between seleected highly resolved plant genomes.

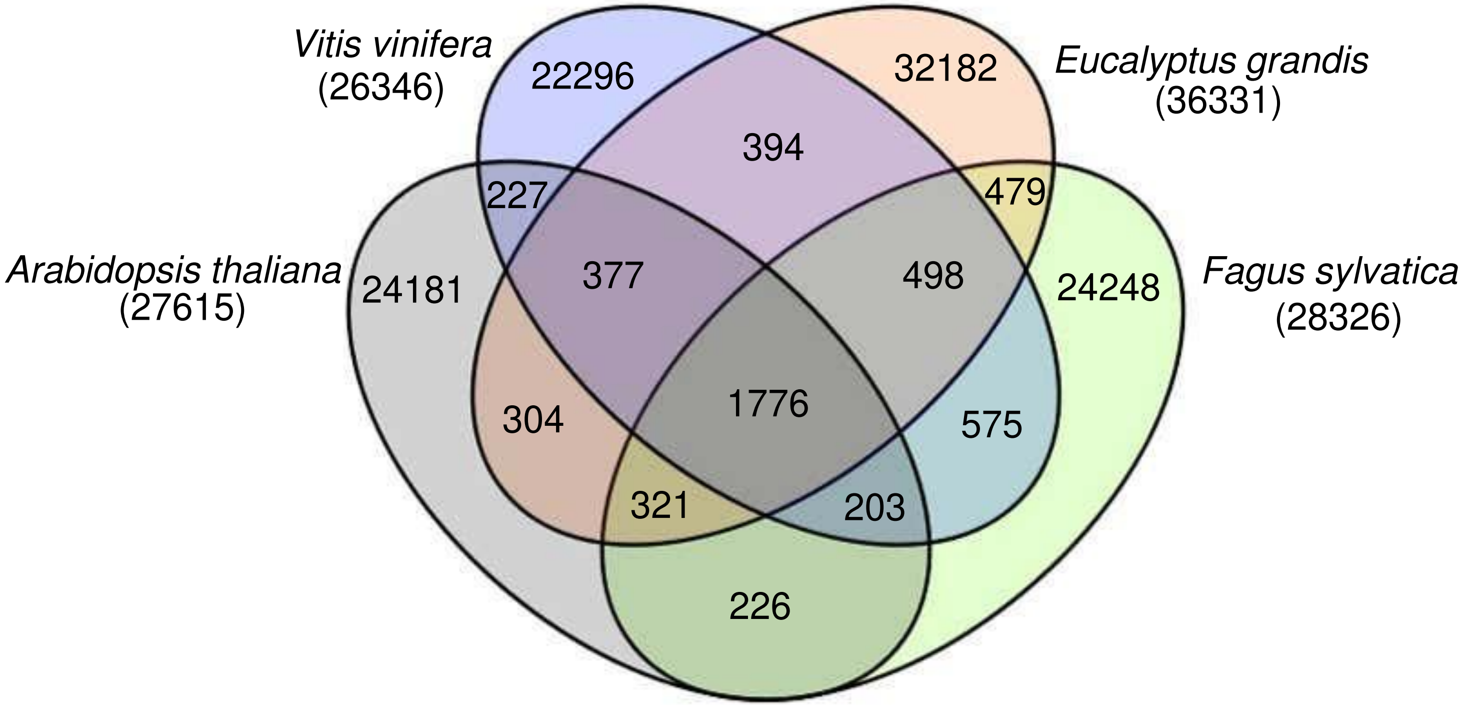

Supplementary File 3. Annotation of centromere-associated features along the 12 chromosomes.

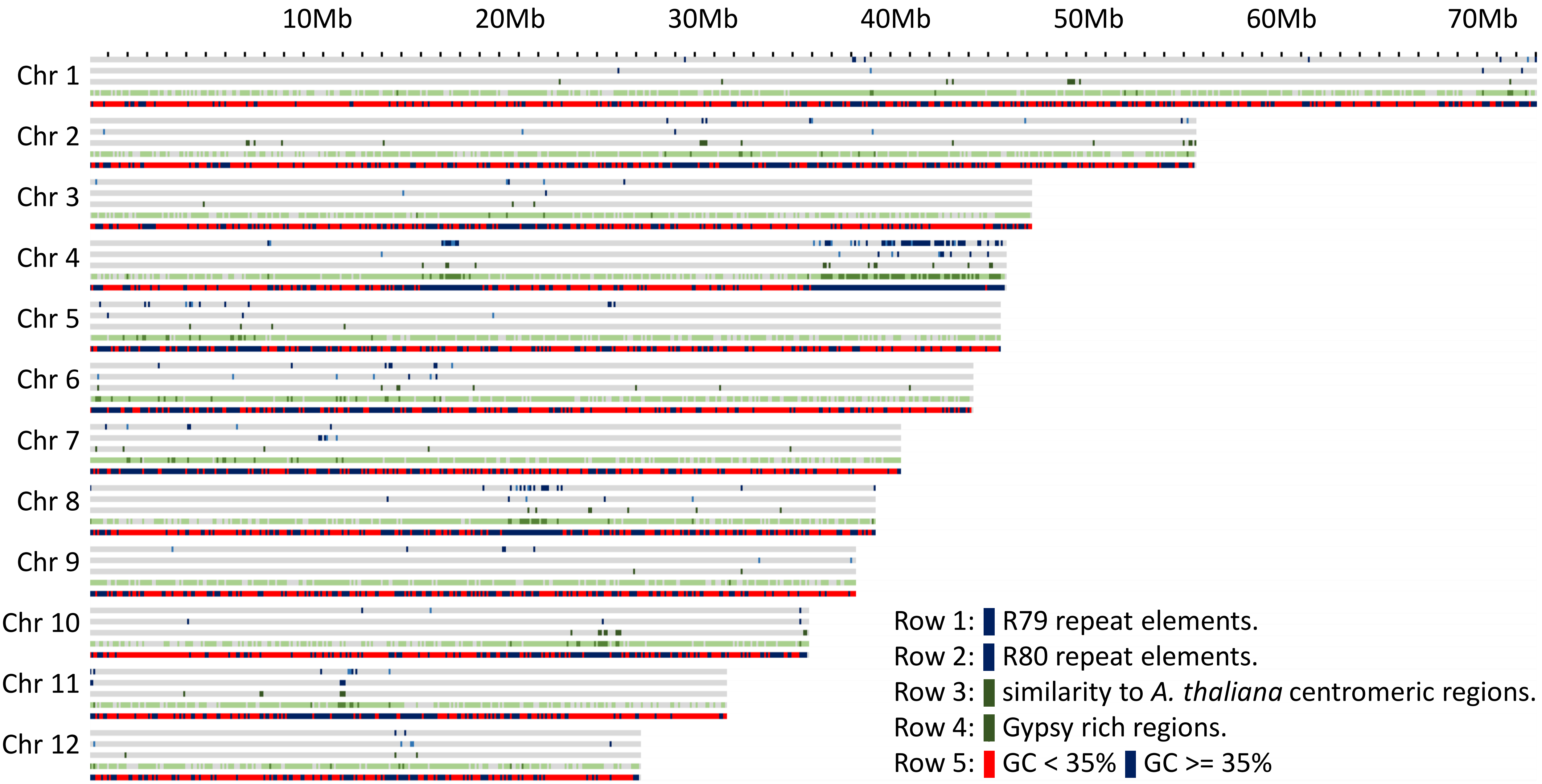

Supplementary File 4. Details of the insertion of organelle genome fragments into the nuclear genome.

Chloroplast Insertions

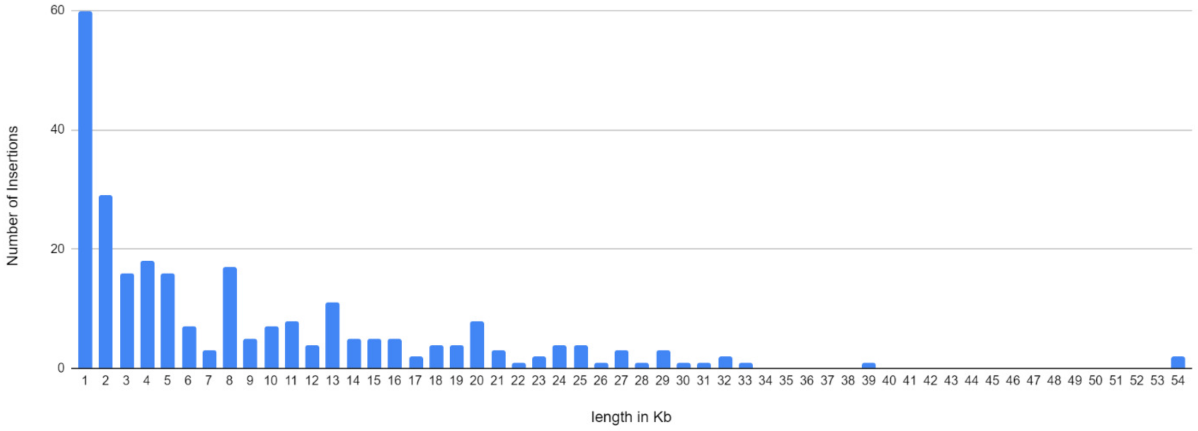

Column chart showing number of insertions for different fragment sizes of chloroplast DNA. The horizontal axis shows the sizes of the fragments (in kb). The vertical axis shows the numbers of insertions for the specified fragment sizes. Inserted fragments less than 1 kb in length are not included in this plot.

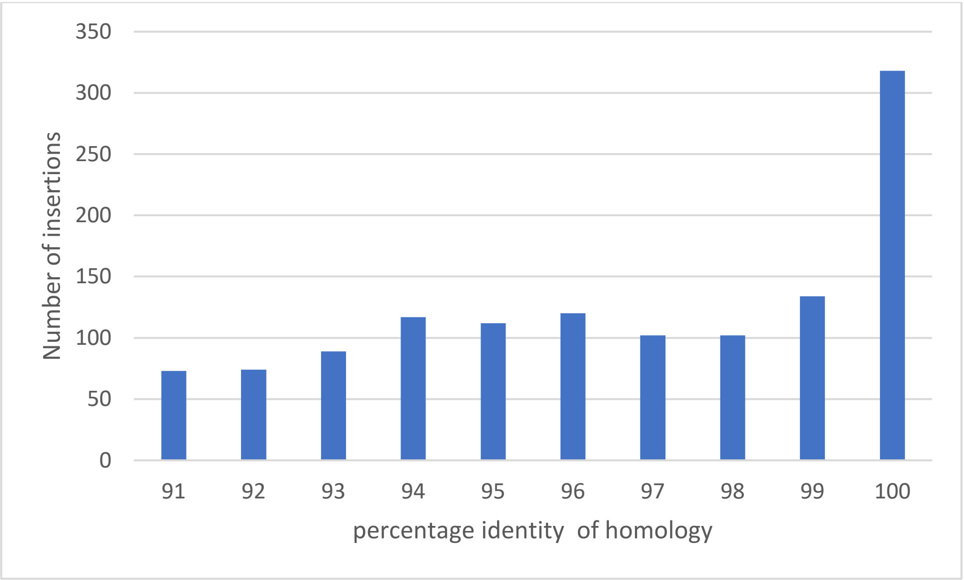

Column chart showing number of insertions at different percentage identity level of the chloroplast genome fragments from 90% to 100% identity. Inserted fragments less than 100 bp in length are excluded for this analysis.

Mitochondrial insertions

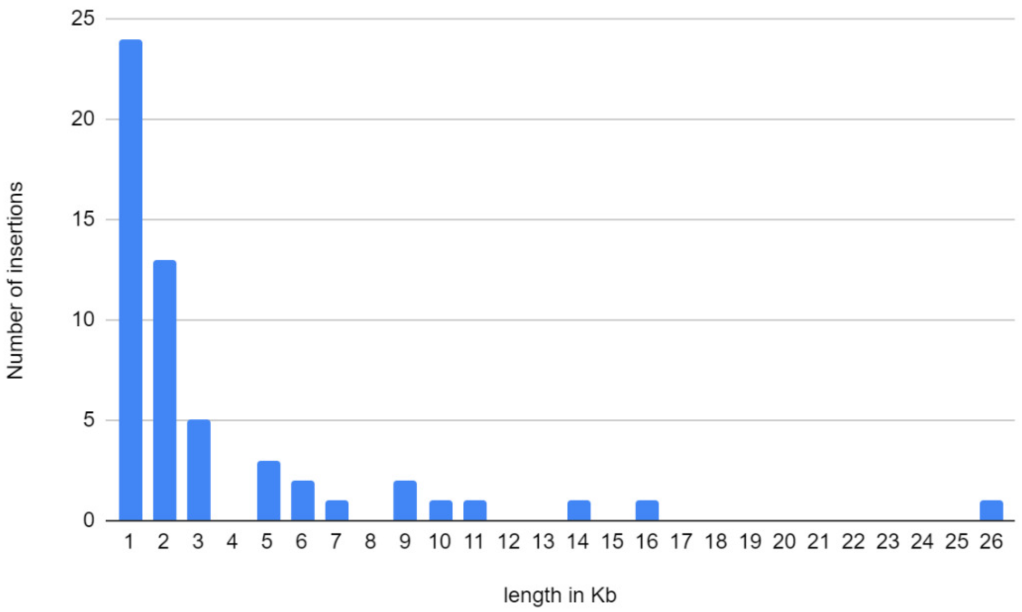

Column chart showing number of insertions for different fragment sizes of mitochondrion DNA. The horizontal axis shows the sizes of the fragments (in kb). The vertical axis shows the numbers of insertions for the specified fragment sizes. Inserted fragments less than 1 kb in length are not included in this plot.

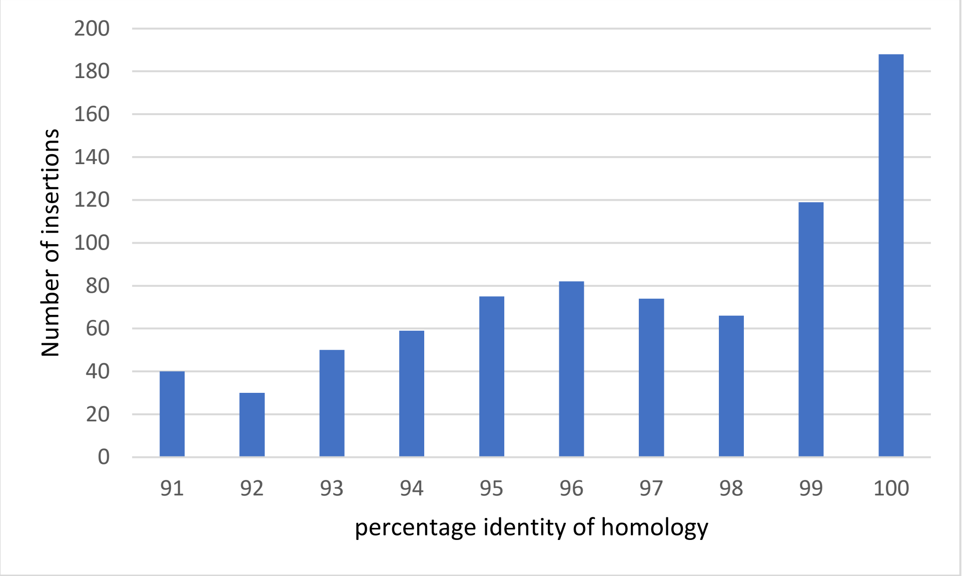

Column chart showing number of insertions at different percentage identity level of the mitochondrion genome fragments from 90% to 100% identity. Inserted fragments less than 100 bp in length are excluded for this analysis.

Supplementary File 5 Multiplexed SNP assay

SNP number	SNP_ID (a)	Locus_Name_pos_SNP (b)	Seq SNP
1	10	00066_edited.merged.removebadseq.renamed-600	TCTACAAAACTAATGAAAGTTCAGCAGCTCTAATGACTGAGGATTCTGGTATTAGGAAGATTCTTCCTAAATCYAAGATATGTGACTTTGAGTTTGACCC[C/T]TGCGGGTACTCCATGAATTCTATCGAAGGYGATGCAATTTCTACAATCCATGTTACACCAGAAGATGGTTTCAGTTATGCRAGCTTTGAAGCAGCTGGCT
2	33	00133_edited_HIrecoded.merged.removebadseq.renamed-637	TTTTCCACTTCAAATGAATTCATTTGGTAACTGCACCATTTGCTGGGAGGTGAAACAGACATCTGTTGTTGGACTTCCCTWACGTGTTAGTGAATTTTGG[A/T]TGAAGTTGAAATGTGTCTACTGCTAAAGGAGGATTCCTTCTAGAGCCACGGAGATGCCTGAGTAGAATCCTGTCTTCTATKGCCATACTTGATGTTCCTT
3	46	00142_edited_HIrecoded.merged.removebadseq.renamed-375	ATATTGTTAATMAGAAWTTTTTTTTTYYCCTCCATCCCYRGCATAGGTAAATGYGTATGAGATGTATTAATCATTCYCCCTCAGAATTGATTTTCTGTGG[C/T]GGCATTTTCAGATGCATGCCTTGTTTTAACTTCWCTGGCTWGYACAGAATGAAGAGTACTCTGAGAAAACCATTTCCCCTTTRAGATAATGCGAGTTTCT
4	50	00142_edited_HIrecoded.merged.removebadseq.renamed-458	GAATTGATTTTCTGTGGYGGCATTTTCAGATGCATGCCTTGTTTTAACTTCWCTGGCTWGYACAGAATGAAGAGTACTCTGAGAAAACCATTTCCCCTTT[A/G]AGATAATGCGAGTTTCTAGTTACTTTGGTTGCCAATTGCTCTT
5	61	091_2_edited_HIori.merged.removebadseq.renamed-231	ACATTTTGCCMCTGCAGCCAGTTGGCCGCYTGGTCTTGAATAAGAACATTGATAACTTCTTTGCAGAAAATGAACARCTTGCATTTTGCCCTGCCATTGT[G/T]GTTCCTGGTGTCTACTACTCAGATGATAAGTTGCTCCAGACTCGTATCTTTTCCTATTCTGATACTCAGAGGCACCGTCTCGGACCAAACTATCTGCAGC
6	64	091_2_edited_HIori.merged.removebadseq.renamed-479	GGTTTCATGAATTTCATGCACAGGGATGAGGAGGTACTTAAAGCTGGCTACTGCATCTTTTAGTTAGTCRGACTTGTATGCTACCTTAGTGCYTTAGGCC[C/T]ACAAATGGTGTAAGATGCTAAGGCRGTATTGCAATTTTATGCTTGTCTAATTYGTGTGAATTTATTCTGATAAGATATTATGATCATAATCTTTAGGTCA
7	81	102_edited_HIrecoded.merged.removebadseq.renamed-745	ATCAGAAGAGTACTGAGCTCCTGATCAGGAAGYTGCCCTTCCAGAGGCTTGTCCGTGAAATTGCCCAGGACTTCAAGGTRTTGTTTTTATTTATTTAGTT[G/T]GGTATTGTTTGGGTTTTGAGTTTGTTTTGTTTTGTTGCTAATGGGTTTGTTTTTATGTTTCAGACTGATCTGCGTTTCCAGAGTCACGCTGTGTTGGCTC
8	87	10_edited_HIrecoded.merged.removebadseq.renamed-381	AGTGTTCTGATGCAAATTGAAGTTAGATTGACTTTCAATGGCTTCTTCAGACTTCCAGTAGTCTGTACCTGTTTCAAAATACTGGTTTGGTCTGTGATTT[C/T]GGTAATTCTACAGGATTGCCATGTAMTTAAATATCGTTATCAAAATGTAAATAAACTGCGTTTTWTTTTATTAAAAATAAAAAAAATAARRGTTTTCCTC
9	101	10_edited_HIrecoded.merged.removebadseq.renamed-978	TTGACAAGAATGCCAAGATAGGAAGAAATGTGGTCATYGCAAATGGTGATGTAAGTTTGGCTCTCTGTGCTCTTTAAATTTTAAATTGCTCTGCACTTCA[A/C]GCTTATCCAAGTTATTGAAGAATRGTGTTTCACTGTTCATTGACACTGTRCTTAMGGAAATTCTTTGACTTCTCAGGGTGTTGAGGAAGCAGATAGGCCA
10	109	110_3_edited.merged.removebadseq.renamed-173	GGTGGCCGAAGAACCAACATCTTCGATCCATTTTCTCTCGACATGTGGGATCCCTTTGATGGTGTTTTCACCTCTGCACTTGCCAATGTACCTGCCTCTG[A/C]ACGTGAAACGTCTGCGTTTGCCAACACAAGGATTGATTGGAGGGAGACCCCAGAAGCTCATGTTTTCAAAGCTGACCTTCCGGGGCTGAAGAAAGAGGAG
11	140	130_edited.merged.removebadseq.renamed-363	GGCTGAAGCTGCTGGGTCTGGAGTTTCTCCATCCCTCAAGAACTTCTTGCTCAGCATTGTGGCTGGTGGTGTTGTGYTTACTRTRATTGTTGGTGCTGTG[A/G]TAGGAGTCTCCAACTTTGATCCAGTCAAGCGGACCTGAGAGACCAATTCTGAGAAGAGAATGCTGTAATTTTATAAATTCACTYTCATGTATCTGTGTCA
12	154	134_2_edited_HIrecoded2.merged.removebadseq.renamed-50	GAGGAAACTGTGGATGTGGTACTGGCTGCAAGTGCGGCAGCGGCTGTGG[A/C]GGGTAATCTTCTAACTTCTTCTGTATTTCATCTTKSTGTTTTCCTAACTATATGCTTAGCATATCCGGCGAGGCTTATTGGTATCACGCGTCGCGAGCGT
13	180	145_2_edited_HIrecoded.merged.removebadseq.renamed-722	AATGAAACGCAAACAGTGTTGTCTTTTYTAATGACAATTAAATGTAATTTAACTTTGTGTTTGAAAGTTTTACCCTATTTACATTATGCAAAAAGCTAAA[A/C]AACATCTTAGCTTTCTAGCCAYATTATCTTGTTCCTCTGCGAATGAGCTTTRGCTCAAATGGGTACTTCCTCCTYCCATAAGAATGKGTRGAGTATTAGG
14	194	148_1_edited_HIrecoded.merged.removebadseq.renamed-378	TGGGACGCCGAGTTYGTCAAAGTTGACCAGGCCACCCTCTTTGATCTCATTCTGGTATAATTACCTTTTTACTCTCTTATTATCGTTTTCTTTGGGCTAG[G/T]KCTTCCAGATTTGGATCTGGATGTTAGTTAGGGTTTCGCTAKYCTGAATTTACGATTCTAGGGTTYCGAAATKTTGAGGATTTTCGATCGAAATTTGCTT
15	221	150_2_edited_HIrecoded.merged.removebadseq.renamed-381	TGTTTATACCATGAATTGTCGTTCTTAATTATATTCCTTTTATTATAAATTTAACCTTTTATRGTTGCCTTCAGCATTCTTTCTTGTTTCTGATTTGACA[A/G]TTGTGAATTTTAACAGGTTTACAAGTCAGGGTCCTTCGGGAGGTCACTGGATATCACCAAATTTACTAGCTACCATGAGTTGCGTGGTGAGTTGGCTCGT
16	226	150_2_edited_HIrecoded.merged.removebadseq.renamed-693	GTTTCCATCATTGTTTGTTGTTTCCTTTTCTTTTATAATTATAACTGCRTGATAGGACYGTTAATTTTTTAAAATTRTCTTTTGGATYGGAAGGCATTTT[A/G]AGTATTCATTTGCATCTCAGAGCCTAAGACTCAATTTAAATCATTTGGACCATCTGATATCAGAATTTTTCTGGCATGTTTCATATGAAAAAAATTAATA
17	231	150_2_edited_HIrecoded.merged.removebadseq.renamed-956	CATTTGTATGATGCTGTAAMTGGTGGTCATGGAGGTCTATATTCTTTATACRAATRTCTTATTTGATTTGGAGATTCTATCCTGATGTCCACACTTAATT[A/G]GYCAAGCTTGATAAGATAGTGATTTTTCCACAACAGGGAATTTGTGAACAGTGTGTGGTGTATCAAAATACTATCACCACAAGAAGTGCAGCAAATGGGC
18	236	154_1_edited.merged.removebadseq.renamed-34	TCCTCATCACCCCTTTGAAAAAAGCCCCAATAT[C/T]ACCACAAATATTTCACACCTACACCTCCACAAACACATCCAAGTAGCCCACTCAACATGCCAAGGCACACTATACCCAGAACTATGCGTCTCAACACTCT
19	260	155_2_edited.merged.removebadseq.renamed-444	ATGCCCAAAAGGTCGTTTTTGCCCCGGGTGATTTTGTCGAAATTACCATTTTCGACCTTGCATCTCTCTGAKATGAAGGAAATTTTCCACGCGCAGGAGA[A/G]ATCTAGTATGGAGCRCGTATTGRTGAACGCGCTGGCRGAGTGTGAGAGGGCTCCGAGCCTGGRTGAGACCAAGCRGTGCGTTGGGTCGGTGGAGGACATG
20	267	155_2_edited.merged.removebadseq.renamed-574	CGCTGGCRGAGTGTGAGAGGGCTCCGAGCCTGGRTGAGACCAAGCRGTGCGTTGGGTCGGTGGAGGACATGATAGACTTTGCYGTCTCGGTCTTGGGCCA[C/T]AATGTRGTTGTGAGGAGCACTGAAAATGTGWGTGGGTCAAAGCAAAACGTGATGATTGGATCAGTCAAASGSATCAACGGTGGAGAAGTGACGAAATCGG
21	283	155_3_edited.merged.removebadseq.renamed-169	TGTCTAGTTATCTGGATCACATTTTGGTACTGGTGATGACTGCATCKCTGTTGGACAAGGAAATTCTCAGGTCACTATTTCAGGTGTCAACTGTGGACCT[C/G]GTCATGGCATCAGGTACTTTTGAAATCTCCAATGCTTGTAACCTCTATGTTTACTTGGGCACATACATCATTTATGTGAAGCTACTTAATGTAATATATT
22	291	17_edited.merged.removebadseq.renamed-1081	ACCATCCACCGGCTTCCATGGCTATGTATGGGGTGACTAAAACAGCCCTTCTTGGTCTTACCAAGGTATGACATGGTTCCCATATCTATGTTTTATGTTA[G/T]TTGTGTATTCAAACAAATCATGGGGTATTCTTCTTTCCTTGTTCTGATTCTTTTTCAATCATCATTTAGGCCCTTGCAGCTGAGATGGCTCCAGATACAC
23	296	19_edited_HIrecoded.merged.removebadseq.renamed-215	CCRACCACCGGTGGAGTCAAGAAGCCCCATCGTTACCGYCCTGGTACTGTTGCCCTCCGGTGAGTCAGCTTATTTATCTCTGTTTTGCYTTTTAATGAAA[C/T]TTGCTTTATTGGTGATTTGCCTTATACGTGTTTGTCAAATTGTTACAGTGAAATCCGTAAGTATCAGAAGAGTACTGAGCTCCTGATCAGGAAGYTGCCC
24	334	23_1_edited_HIori.merged.removebadseq.renamed-741	KATTTAAATAGCATATGTGCAYGAATATGCTTGRTTTTTSRTGATTATCCTTACATTCTTATGGAAACATTTTGGCMAAAACCGTCTTGTTTTTGGATGA[A/G]TAAGTAATTTATTCAAACCAGAAGAGACTTGCAAGCAGGTTACMAKAAGAAACWGCAAGCCTTRACACTCAATACAGCTCCAMAAACTTCAAACAAAAAT
25	340	23_1_edited_HIori.merged.removebadseq.renamed-852	ATTCAAACCAGAAGAGACTTGCAAGCAGGTTACMAKAAGAAACWGCAAGCCTTRACACTCAATACAGCTCCAMAAACTTCAAACAAAAATCACTAATATA[A/C]ATTAGACTCCACAAAGCAAGGACCATTTTGTTATGCTAATGATTACATATATGCTCATTTTGATGTGAACTCTTAGGGGATTGAGATTTTCATGATGTTT
26	375	30_2_edited_HIrecoded.merged.removebadseq.renamed-435	GAGTATTATTTGACTCCCAAACTAAACCATGTGGGCTACATAATAATATTGTTGTTCYTTTTTTATGTTTTTKGGTTAAATNATGATCTCATGGGCTAGA[A/C]ATGGGTGTTGGCTGCAGGTGTGGAGCATGTTGGGGGAGACATGTTTGTTAGTGTTCCTAAAGGAGATGCCATTTTCATGAAGGTGGGTTTGWACTTATCT
27	384	30_2_edited_HIrecoded.merged.removebadseq.renamed-936	TAYTTTTTGTGGAAAGACACTTTAACCTATTAACTAGCACATGAATCATAAGGTGAAAATGAAATTTAAAGAAAAATTGATTAGTCAAAGACATTTGATC[C/T]TTGWTTATGCTAYTGACATGTGTGTGCTCCTATGTACAGTGGATATGCCATGATTGGAGTGATGAGCACTGCTCAAAATTTTTGAAGAACTGCTATGATG
28	387	30_2_edited_HIrecoded.merged.removebadseq.renamed-1173	GTATTCTTCCTGTAGCCCCGGACAGTAGCCTTGCTGCTAAGGGAGTCATCCATATTGATGTTATCATGTTGGCTCATAATCCGGGTGGGAAAGAGAGGAC[C/T]GAGAAGGAGTTCGAGGMCTTGGCYAAAGGGGCAGGATTTCAAGGGTTCAAAGTAGTGTGTTCTGCTTTCAACACTTACATCATGGAATTCCTTAAAAAGM
29	395	39_edited.merged.removebadseq.renamed-256	GTCCTGCAACTCATCGGTCCTATAGGTGTATTGTCAGGTATGGATATCGCGATGTTCATCAGGATATCCATTCCTTTGAAACTGAGCTAGTTGAAAGACT[A/C]GCTGATTTCATTCGCTATGATTGGCRKAGAACAAATGGTACTAATTCTTGCAATGAGGATGATGCATCAAGATCTAATGAATCAACTAGTGAGTGTAGAT
30	412	50_edited_HIrecoded.merged.removebadseq.renamed-320	AGATGACGTGGARGCTGGTTCTGACGTGTCATTATGGAGTTATTCAATTTGATTGTTATTACTATTAATTATAACAATCATGTYGTAATAYATGTTAGAA[A/C]GTTTGGTTTAGCATTATAGCTGCTTGCATTTTAGACAGTCTGATCATTGTTACTTGTTAGGGAAAGAATTGTTACTTTCCGTGCTTATTGTTTTTGATGA
31	435	52_1_edited.merged.removebadseq.renamed-235	CAAACCGCYGTACTCATYGAAACCCTCACTGCTCTCGGCGCTGAGGTTCGTTGGTGTTCATGCAACATCTTCTCAACCCAAGACCACGCCGCCGCCGCCA[C/T]CGCTCGCGACWCYKCTGCTGTGTTCGCGTGGAAGGGAGAGACWCTBCAGGAGTACTGGTGGTGTACCGAGCGTGCACTTGATTGGGGCCCAGGTGGTGGA
32	440	52_1_edited.merged.removebadseq.renamed-350	CTGCTGTGTTCGCGTGGAAGGGAGAGACWCTBCAGGAGTACTGGTGGTGTACCGAGCGTGCACTTGATTGGGGCCCAGGTGGTGGACCCGATCTCATCGT[C/T]GATGAYGGTGGTGATGCYACTTTGTTGATCCATGAGGGTGTGAAAGCTGAGGAGATCTATGAGAAGACTGGTACCGTTCCAGACCCTTCATCYACAGACA
33	444	52_2_edited.merged.removebadseq.renamed-65	ATGTYATGATTGCCGGAAAGGTTGCCGTTGTCGCTGGATATGGTGATGTGGGAAAGGGTTGTGC[A/T]GCTGCCTTGAAACAAGCTGGAGCCCGTGTCATCGTSACTGAGATTGATCCAATTTGTGCTCTTCAGGCCCTTATGGAAGGCCTCCAAGTTCTAACCCTYG
34	470	58_edited_HIori.merged.removebadseq.renamed-736	AGAACTTTTTTCAATGACCTCAATCTTTGTATACTTTCATGCTGCTTAACTTTTTTTGGTGATGCAGGGGAGAGATTGGGAAATGGATTGCTTTYGTTGC[A/T]GTTATATTGCGCCTTTTTTTCCCACGTCATTTCCCAGGTATAATATAAGTTTCACTCACTATTGATTGATCGTGTTTATGCRTTAATGCTTCTAAGTAAT
35	477	60_edited_HIrecoded.merged.removebadseq.renamed-249	YTGCTAGTGCCCAGGGAAGCCTTGTTGATGAGATTAGGGAATACCTGAAGAAGGGTTCCTCTGCAAGTCAGCTGAAACCGTTTTTGGATTCACTCTCTGG[G/T]ACTTCCCAAGAAKTCATGGATGCCCTRTTYRAAGCTCTCTTTGGGGGTCTTGGAAAGGGGTTTGCTAAAGAGGTGACCAAGAAGAAGAACTTCCTTGCAG
36	502	68_edited.merged.removebadseq.renamed-321	GTTGATGTTTCAGTGGTTGACCTCACTGTGAGGCTGGAGAAGAAGGCTACCTATGAWCAAATTAAAGCTGCTATCAAGTAAGGGCAGTGAATMATTTTTT[G/T]TTATTTGCTTTTAGTGTCTGTTGTATGTATGGTAGGTTGTATTGCTATGTGGGTGATGAACGTTTAGTTGCTTTACCWTCTGACAATTGTGTGGTGAAAG
37	504	70_edited.merged.removebadseq.renamed-119	AGCTCCCAAAGGACACTTCACGCTCATTCCAAGCTCCAACTGGCTGGTCCGGGCGTTTCTGGGCCCGAACCGGGTGCAACTTCGACGGATCCGGAGCCGG[A/G]TCATGTCTCACTGGCGACTGTGGTTCAGGCCAAGTAGAATGCAACGGGTTCGGAGCCGCCCCACCCGCAACTCTCGCGGAGTTCACACTCGGYTCTGGCG
38	511	73_edited_HIori.merged.removebadseq.renamed-166	TGCAGAAGATGAGCTCATGAGAATCCTYSTTCTTGTCCGAAACTTCTTACCTGGATACCATCAAGCTATTAGTGGGGAATGGAATGTTGCAGCTATTGCT[C/T]ACAGGGCTTATGATCTTGAGGGAAAGACAGTTGGAACKRTTGGTGCTGGACGAATTGGCAAACTTTTACTTCAACGTTTGAAACCTTTTAACTGTAATCT
39	515	73_edited_HIori.merged.removebadseq.renamed-615	GATCTCAATTTTGTTAGATACTATGTTGTGATACAATTTGAATTTGTTTGCTGTAGAATGTCATATTTCTATGTTACTTATARGCTATCATTATGTTGGT[G/T]TATGCACTGAAATTCAAACTTATGAGAATTCTGATCTTGTGATTCANNGGGTTATTTGACAAANNNNNNNNNNNNCACATCTCTTGTTAGGCTATATTATT
40	535	88-1_edited.merged.removebadseq.renamed-727	TGGGACCATTCTCAGAGCAAACTCCCACATACCTGACTGGTGAATTCCCTGGTGACTATGGCTGGGACACTGCTGGTTTATCAGCAGACCCTGAGACCTT[C/T]GCAAAGAACCGTGAGCTCGAGGTGATCCACAGCCGATGGGCCATGCTTGGAGCTCTGGGCTGTGTCTTCCCTGAASTCCTTTCAAAGAATGGTGTCAAGT

SNP_ID (a) following Ouayjan et al. (2018). SNPs discarded from the analyses are highlighted in yellow; Locus_Name_pos_SNP (b) corresponds to the locus name given by Lalagüe et al. (2014); seq SNP corresponds to sequences of the SNP flanking regions. The targeted SNP is indicated in brackets [/].
